# Childhood socioeconomic disadvantage and adult multimorbidity: A systematic review and meta-analysis

**DOI:** 10.1177/26335565261447702

**Published:** 2026-04-29

**Authors:** Philip Broadbent, Alistair Carr, Erik Igelström, Valerie Wells, Anna Pearce, Daniel Kopasker, Srinivasa Vittal Katikireddi

**Affiliations:** 1 School of Health and Wellbeing, 347493 University of Glasgow, Glasgow, UK

**Keywords:** multimorbidity, socioeconomic circumstances, childhood poverty, health inequalities

## Abstract

**Background:**

Childhood socioeconomic disadvantage is linked to individual chronic diseases in adulthood, but its relationship with multimorbidity remains unclear. Understanding this is crucial for informing prevention strategies and the economic case for investment. This review and meta-analysis evaluated the association between childhood disadvantage and adult multimorbidity.

**Methods:**

Following pre-registration (PROSPERO: CRD42024588657), we searched MEDLINE, SocIndex, ASSIA, and ProQuest Public Health to March 2025 for studies assessing childhood socioeconomic circumstances (SECs) and adult multimorbidity (≥2 chronic conditions). Risk of bias was assessed using ROBINS-E and evidence certainty using GRADE. Random-effects meta-analysis and synthesis without meta-analysis (SWiM) were conducted. Subgroup analyses explored heterogeneity by region, design, and exposure type.

**Results:**

From 5,617 records, 10 studies met inclusion criteria. Most were cross-sectional, using retrospective reports of exposure and self-reported outcomes. Exposures included perceived childhood economic adversity (n=6), parental education (n=4), parental occupation (n=1), and composite measures (n=3). Meta-analyses found no clear associations for perceived adversity (OR 1.08, 95% CI 0.87–1.23; I^2^ = 94.4%) or parental education (father’s (Odds ratio (OR) 0.95, 95% CI 0.66–1.37; I^2^ = 66.8%); mother’s (OR 1.07, 95% CI 0.70–1.61; I^2^ = 36.9%)). Relative Index of Inequality estimates generally indicated higher mortality risk with greater childhood disadvantage, though effect sizes varied widely and some studies suggested the reverse.. All studies were high/very high risk of bias with very low certainty.

**Conclusions:**

Evidence for an association between childhood socioeconomic disadvantage and adult multimorbidity is limited and uncertain. Findings suggest possible harmful effects but remain constrained by methodological weaknesses and heterogeneity. High-quality longitudinal studies with standardised multimorbidity definitions are needed.

## Introduction

Multimorbidity, the co-occurrence of two or more chronic conditions within an individual, affects approximately one in three adults in community settings and more than half of those aged 65 years and older.^1–7^ It is associated with reduced quality of life, accelerated functional decline, and substantially increased healthcare costs driven by complex care needs and polypharmacy.^8–18^ These consequences are exacerbated by healthcare systems designed around single diseases rather than the integrated management required for people with multiple long-term conditions.^19^ Childhood socioeconomic disadvantage (including economic adversity, parental circumstances (including education, occupation, and employment status), and material deprivation) is a well-established determinant of health across the life course, with adverse early-life conditions consistently associated with elevated risk of physical and mental health problems that accumulate and persist into adulthood.^20–22^

The burden of multimorbidity is unevenly distributed across society. In Scotland, onset occurs 10–15 years earlier among people living in the most deprived tenth of areas compared with the least deprived, and is particularly associated with comorbidity involving mental health disorders.^23^ Findings from the MRC 1946 British birth cohort similarly show that both childhood and adult socioeconomic disadvantage independently predict multimorbidity, with the most disadvantaged accumulating 1.2 to 1.4 times more conditions than the most advantaged.^24^ These patterns indicate that social determinants operating across the life course shape the accumulation of chronic disease, though the nature of that accumulation varies considerably across global contexts, as populations shift from infectious to chronic non-communicable disease burdens at different rates and stages (the epidemiological transition).

To our knowledge, no systematic review has yet synthesised findings on childhood socioeconomic circumstances and adult multimorbidity. This gap matters because multimorbidity represents systemic vulnerability, requiring an understanding of early life and upstream determinants.^25^ Clarifying whether these associations reflect sensitive-period effects, where exposures during key developmental stages have heightened influence, or cumulative exposure across multiple life stages has important implications for prevention strategies across the life course.^26^

This systematic review synthesises evidence on the association between childhood socioeconomic disadvantage and multimorbidity in adulthood. Its objectives are to: (1) quantify associations between childhood socioeconomic circumstances and adult multimorbidity; and (2) examine whether findings vary according to the definitions, measurements, and settings used across studies.

## Methods

### Study design and registration

This systematic review was registered prospectively on PROSPERO (registration number: CRD42024588657) and is reported according to the PRISMA 2020 guidelines.

The review followed a structured approach using the PECO framework: ‘Population’ included studies of adults (≥18 years) with data on childhood socioeconomic circumstances; ‘Exposure’ encompassed various measures of childhood socioeconomic disadvantage (e.g., parental occupation, education, income, perceived adversity); ‘Comparison’ involved different levels of childhood socioeconomic circumstances, as well as presence versus absence of particular socioeconomic disadvantages where relevant; ‘Outcomes’ focused on adult multimorbidity defined as the co-occurrence of two or more chronic conditions.

### Search strategy and study selection

We systematically searched MEDLINE, SocIndex, ASSIA, and ProQuest Public Health from inception to March 2025, using combinations of terms related to childhood socioeconomic conditions, child poverty, and adult multimorbidity. There were no language restrictions. The search strategy was developed with an information specialist and reviewed by experts in the field before implementation. Search terms were adapted for each database while maintaining consistency in core concepts. Backward citation searching was carried out. The full search strategy for each database is provided in Appendix A.

Titles and abstracts were screened independently by two reviewers (PB and one of EI or AC), with full-text review of potentially relevant studies conducted by the same reviewers. Discrepancies were resolved through discussion, with a third reviewer (EI or AC) consulted when consensus could not be reached. References of included studies and relevant review articles were hand-searched to identify additional eligible studies.

To avoid duplication from multiple studies using the same datasets, we applied a structured framework to prioritise included data. We first identified studies that best aligned with our PECO criteria and had extractable effect estimates. Among these, we then prioritised studies based on methodological quality. Full details of this decision process are provided in Appendix B.

### Inclusion criteria

We included longitudinal studies (and cross-sectional studies with retrospective reporting) examining the association between childhood socioeconomic conditions and adult multimorbidity. Adult multimorbidity was operationalised as the presence of two or more chronic conditions. Consistent with contemporary definitions in the literature, this included both physical health conditions (e.g. cardiovascular disease, diabetes, respiratory conditions) and mental health conditions (e.g. depression, anxiety disorders).^2,6,23^ This is distinct from ‘comorbidity’, which typically refers to additional conditions occurring alongside a primary index disease; multimorbidity, by contrast, treats all conditions equally without designating a primary condition. Further details on the definition and measurement of multimorbidity are outlined in Appendix C. Eligible exposures included perceived childhood economic adversity, parental education, parental occupation, parental income, and composite indicators of childhood socioeconomic status (SES). The outcome was defined as adult multimorbidity, operationalised as the presence of two or more chronic physical or mental health conditions. Studies had to report either quantitative effect estimates (e.g., odds ratios, relative risks) or crude proportions of multimorbidity by exposure group from which unadjusted quantitative estimates could be calculated.

### Exclusion criteria

Studies were excluded if they: (1) focused solely on single disease outcomes rather than multimorbidity; (2) examined only adult socioeconomic position without childhood measures; (3) were not peer reviewed, or were case reports, editorials, commentaries, or reviews; (4) did not provide sufficient data to extract or calculate effect estimates; (5) used exclusively clinical or convenience samples that were not representative of broader populations; or (6) did not allow multimorbidity to be studied as an outcome (e.g., studies that only reported single disease trajectories or condition counts without data on participants with ≥2 conditions).

### Data extraction

Data extraction was performed independently by two reviewers using a piloted standardised form developed specifically for this review (Appendix D). Extracted data included: study characteristics (author, year, country, study design, sample size, age range); population characteristics (demographics, recruitment method, response rates); exposure definitions and measurement methods; outcome definitions and assessment approaches; effect estimates with confidence intervals and adjustment variables.

For studies reporting results across multiple exposure categories or subgroups, we extracted data for all relevant comparisons. When studies reported both crude and adjusted estimates, we prioritised adjusted estimates that most closely matched our target confounding set (age, sex, race/ethnicity) while avoiding adjustment for potential mediators such as adult socioeconomic status, health behaviours, or BMI. This confounder set was determined a priori using a directed acyclic graph (DAG; see Appendix E). Where studies provided multiple adjustment models, we selected the estimate closest to our specified confounding set, with any over- or under-adjustment reflected in the results and risk of bias assessment. Details of adjustment sets for each study and our selection rationale are provided in Appendix F. Disagreements between reviewers were resolved through discussion, with a third reviewer consulted when necessary.

### Risk of bias assessment

Risk of bias was assessed using the ROBINS-E (Risk Of Bias In Non-randomised Studies - of Exposures) tool, developed by the Cochrane Methods Group and specifically designed for observational studies examining exposure effects.^27^ The tool evaluates seven domains: bias due to confounding, bias in selection of participants, bias in exposure classification, bias due to deviations from intended exposures (whether participants’ observed exposure differed from the intended measurement, for example due to changes in socioeconomic circumstances during the exposure period), bias due to missing data, bias in outcome measurement, and bias in selection of the reported results.

Each domain was rated as ‘low risk’, ‘some concerns’, ‘high risk’ or ‘very high risk’ of bias. Overall risk of bias was determined by considering judgments across all seven domains, with the default judgment matching the domain with the greatest risk of bias, though assessors could override this when the totality of concerns across multiple domains suggested a higher overall risk. Assessment was conducted independently by two reviewers, with disagreements resolved through discussion. No studies were excluded on the basis of risk of bias, but this information was incorporated into the synthesis and GRADE assessment.

### Data synthesis and analysis

#### Meta-analysis

We conducted random-effects meta-analyses using the restricted maximum likelihood (REML) estimator for both model fitting and estimation of between-study variance, for studies that examined the same SECs measure and provided compatible effect measures. Heterogeneity was assessed using the I^2^ statistic, with values of 25%, 50%, and 75% representing low, moderate, and high heterogeneity, respectively.

Owing to heterogeneity in the composite socioeconomic measures, studies using these were included in narrative synthesis only.

#### Relative Index of Inequality (RII) calculation

Where studies reported the number of cases and non-cases of multimorbidity across ordered exposure categories, we calculated the Relative Index of Inequality (RII), a regression-based measure that summarises the socioeconomic gradient in risk across the full distribution of disadvantage.^28^ Depending on data availability, RII was estimated using binomial regression when raw counts were available, weighted linear regression on log Odds ratios when multiple adjusted Odds ratios with standard errors were reported. The RII is interpreted as the odds ratio of multimorbidity comparing the notionally most disadvantaged to the notionally most advantaged individuals.

#### Synthesis without meta-analysis

Where meta-analysis was not feasible, narrative synthesis was guided by SWiM (Synthesis Without Meta-analysis) recommendations.^29,30^

#### Effect direction analysis

For each study, we classified the direction of effect as “harm” (disadvantage associated with higher likelihood of multimorbidity), “benefit” (disadvantage associated with lower likelihood), or “equivocal” (effect size suggests minimal clinical importance). We also conducted a binomial sign test to explore whether the proportion of studies reporting a harmful effect exceeded what would be expected by chance under a null hypothesis of no directional trend. Results of the test are reported as two-sided p-values, without applying a fixed significance threshold, and are interpreted alongside the precision of effect estimates and the overall strength and consistency of the evidence.

#### Secondary and sensitivity analyses

We conducted pre-specified subgroup analyses to explore heterogeneity by geographic region, study design (cross-sectional vs. longitudinal), and exposure type. Additionally, we performed leave-one-out sensitivity analyses to assess the influence of individual studies on pooled estimates.

### Statistical software

All analyses were conducted in R (version 4.4.1) using the metafor package for meta-analysis and the dplyr package for data manipulation. Forest plots were created using ggplot2, and effect direction plots were generated using custom R code. All analysis code is available in Appendix N and on GitHub (https://github.com/philip-broadbent/Multimorbidity).

### GRADE assessment

We used the Grading of Recommendations Assessment, Development and Evaluation (GRADE) approach to assess the certainty of evidence for each exposure-outcome relationship. When using ROBINS-E for risk of bias assessment, evidence from observational studies began as high certainty and was evaluated across five domains: risk of bias, inconsistency, indirectness, imprecision, and publication bias. Due to risk of bias concerns, evidence was typically downgraded by at least two levels. Evidence certainty could be upgraded for large effect sizes, dose-response relationships, or when plausible confounding would strengthen the observed effects.^31^ Following GRADE guidance, we calculated absolute risk differences to support interpretability of findings and inform decision-making. Details of this conversion process are provided in Appendix M.

### Ethical considerations

Ethical approval was not required as the review used only publicly available data.

## Results

### Study selection and characteristics

**Figure 1.**
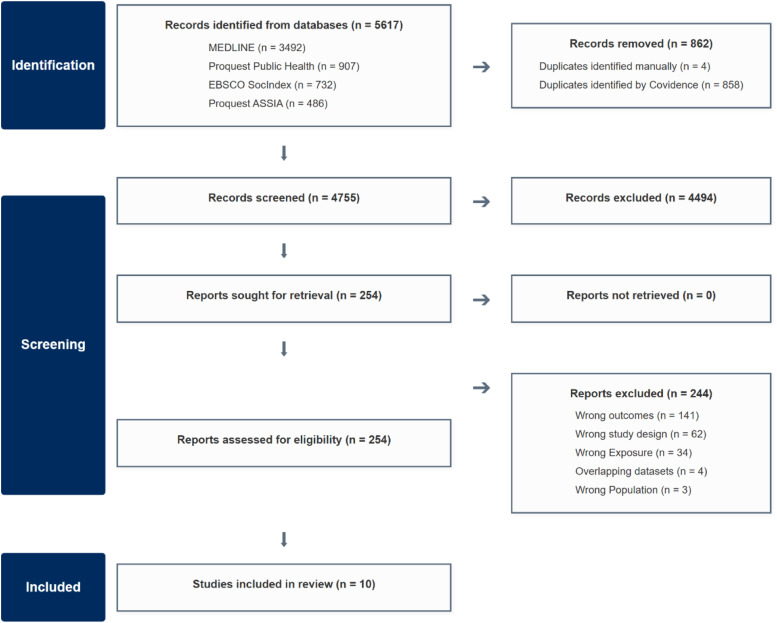
PRISMA (Preferred Reporting Items for Systematic Reviews and Meta-Analyses) 2020 flow diagram illustrating the study selection process for systematic review of associations between childhood socioeconomic disadvantage and adult multimorbidity.

From 5,617 records identified through database searches, 254 full-text articles were assessed for eligibility. Fourteen studies met all primary inclusion criteria. Four pairs of studies used overlapping datasets from the same underlying cohorts (SHARE, NCS-R, HRS, and CHARLS). To avoid duplicating participant data, we applied a prioritisation framework (detailed in Appendix B) and excluded 4 of these overlapping studies. This resulted in 10 unique studies being included in the final review. The study selection process is detailed in Figure 1.

### Study characteristics

The characteristics of included studies are summarised in Table 1. Studies covered a broad geographic range: Latin America (Colombia, Brazil), Asia (India, China), Africa (Botswana), Europe (multiple countries), and North America (USA). Sample sizes ranged from 1,178 (Keetile et al.) to over 100,000 (Schramm et al.). Female representation ranged from approximately 48% to 69%, with most studies reporting gender-balanced samples.

**Table 1.** Characteristics of included studies summarising study design, population, exposure measures, outcome definitions, effect estimates, and risk of bias.

Author (year)	Country	Study design	Sample size	Years of data collection	Age at outcome measurement	% Female	Exposure	Multimorbidity (MM) definition	MM measurement	Number of data points	Type: Effect estimate (95% confidence interval)	Risk of bias
Ballasteros 2021	Colombia	Cross-sectional	17,571	2015	60 years of age or older (mean age 70.8 years, SD=8.2)	57.3	Retrospective self report (2 category, perceived childhood economic adversity)	2+ Chronic conditions.	Predominantly self-report with one validated scale (Depression assessed using Short Form of Geriatric Depression Scale)	1	Poor vs. Not poor childhood economic situation *Odds ratio* (*OR):* 1.34 (1.23, 1.47)	Very High
Dekhtyar et al. (2019)	Sweden	Cross-sectional	2589	2001-2013	Age at baseline: ≥60 years Follow-up occurred over 9 years, so multimorbidity assessed at ages 60-69+ depending on baseline age	61.1	Retrospective self report (2 category, father’s occupation (manual vs. Non-manual))	Not defined, studied rate of chronic disease accumulation	Clinical examinations, medical histories, laboratory data, medication use, and register linkages with physician verification	1	Manual vs. Non-manual father’s education *OR:* 1.1 (0.94,1.30)	Very High
Haas (2008)	Multiple European countries *	Cross-sectional	Varies by country*	2004 (SHARE and ELSA), 2009 (TILDA)	≥50 years (mean age varied by country: 62.6-65.6 years at baseline)	Varies by country*	Composite^a^	2+ Chronic conditions	Self-reported physician-diagnosed chronic conditions	1	*One unit increase in childhood disadvantageOR:* 1.08 (1.04, 1.10)	High
Henchoz et al. (2019)	Switzerland	Cross-sectional	4731	2004, 2009, 2014 (baseline); 2006, 2011, 2016 (follow-up for multimorbidity assessment)	67-72 years (multimorbidity assessed at 2-year follow-up; baseline age 65-70 years)	58	Retrospective self report ( 2 category, perceived childhood economic adversity	2+ Chronic conditions	Self-reported physician-diagnosed chronic conditions requiring treatment or causing symptoms in previous 12 months	1	Poor vs. Not poor childhood economic environment *OR:*1.23 (1.02, 1.48)	Very High
Keetile et al. (2023)	Botswana	Cross-sectional	1,178 adults (≥15 years)	March 2016	≥15 years (mean not specified, but 69.1% aged 15-44 years)	69.10%	Composite^b^	2+ Chronic conditions	Self-reported physician-diagnosed chronic conditions	2	Low vs high childhood SES *OR* = 1.78 (1.11 to 2.68)	Very High
											Middle vs high childhood SES OR = 1.32 (0.29 to 1.79)	
Pati & Sinha (2023)	India & Brazil	Cross-sectional	India: 51,481; Brazil: 8,730	India: 2017-2018; Brazil: 2015-2016	≥50 years (India: mean not specified; Brazil: mean not specified)	India: 53.49% Brazil: 53.83%	Retrospective self report (2 category, perceived childhood economic adversity)	2+ Chronic conditions	Self-reported physician-diagnosed chronic conditions	2	India: Poor vs. pretty well off childhood economic status *APR:* 0.76 (0.65, 0.88)	High
											Brazil: Poor vs. pretty well off childhood economic status *APR:* 1.08 (0.97, 1.19)	
Pavela 2016	USA	Longitudinal	10,584	1992-2008 (waves 1-9 of the Health and Retirement Study)	Mean age 54.9 years at baseline (1992)	55%	Retrospective self report (2 category Perceived childhood economic adversity. 2 category parental education. 2 category parental employment status.)	NA - number of chronic conditions	Self-reported physician-diagnosed chronic conditions	4	Low vs. High childhood SES *OR:* 1.05 (0.95,1.16)	High
					Multimorbidity assessed across multiple waves from 1992-2008						Mother <High school vs. >High school education *OR:* 1.19 (1.00, 1.41)	
				Biennial follow up surveys every 2 years	Age range at baseline: 23-82 years						Father <High school vs. >High school education *OR:* 1.10 (0.93,1.30)	
											Father ever unemployed 1.11 (0.99, 1.24)	
Putnam et al. (2013)	USA	Cross-sectional	5692	Feb 2001 – Apr 2003 (NCS-R Part 2).	Aged ≥18 at survey; lifetime diagnoses used to assess multimorbidity up to interview.	53%	*Composite^c^	Presence of disorders in two or more DSM-IV diagnostic domains	Structured WHO-CIDI interview yielding lifetime DSM-IV diagnoses	1	Childhood economic adversity vs. No childhood economic adversity; OR = 2.10 (95% CI: 1.80–2.45)*OR:* 2.10 (95% CI: 1.80, 2.45)	Very high
Schramm (2021)	Denmark	Longitudinal	102818	Baseline data from 2010	Age groups:	48.3	Administrative records (3 category parental education at age 5)	Register-based Co-occurance of two or more medical conditions	National register data on 47 chronic conditions (diagnoses & prescriptions)	1	‘Short’ vs. ‘Long’ Parental education *OR*: 1.68 (1.59, 1.78)	Very high risk of bias
				8-year follow-up (2010-2018)	40-44 (18.2%),							
				Multimorbidity data collected in 2018	45-49 (20.9%),							
					50-54 (24.0%),							
					55-59 (21.2%),							
					60-63 (15.8%)							
Zhao (2023)	China	Cross sectional	7,578	Multimorbidity data collected in 2015 (CHARLS wave 3)	Mean age: 68.25 ± 6.70 years	49.68	Self-reportretrospective	The coexistence of ≥2 of 14 specified chronic diseases	Self-reported physician-diagnosed chronic conditions	2	Financial situation worse vs. Better than average *OR:* 1.32 (1.08, 1.61)	High
				Early-life factors collected in 2014 (CHARLS Life History Survey)	Range: ≥60 years old		(3-category financial situation, 3 category parental education)				Father’s education Illiterate vs. High school or above *OR: 0.75 (0.5, 1.11)*	

Only two of the ten studies (Pavela et al. and Schramm et al.)^32,33^ used longitudinal designs, both conducted in high-income countries (USA and Denmark). The predominance of cross-sectional studies increases susceptibility to recall bias.

Perceived childhood economic adversity was the most commonly assessed exposure, examined in five studies. Other exposures included parental education (four studies), parental employment status (one study), parental occupation (one study), and composite childhood socioeconomic measures (three studies). Composite measures varied substantially in their construction, combining indicators such as parental education and occupation, food insecurity, perceived childhood health, and broader adversity indices.

Most studies defined multimorbidity as the presence of 2 or more chronic physical conditions, often based on self-report. Notable exceptions included Putnam et al., who defined multimorbidity as disorders across 2 or more DSM-IV psychiatric domains; Dekhtyar et al., who focused on the rate of chronic disease accumulation rather than a binary multimorbidity outcome; and Pavela et al., who used the number of conditions as a continuous variable rather than applying a specific threshold.

Several studies provided multiple estimates based on different exposure levels or different exposure types within the same dataset, contributing to the 16 total exposure-outcome estimates included in the review.

### Risk of bias assessment

**Figure 2.**
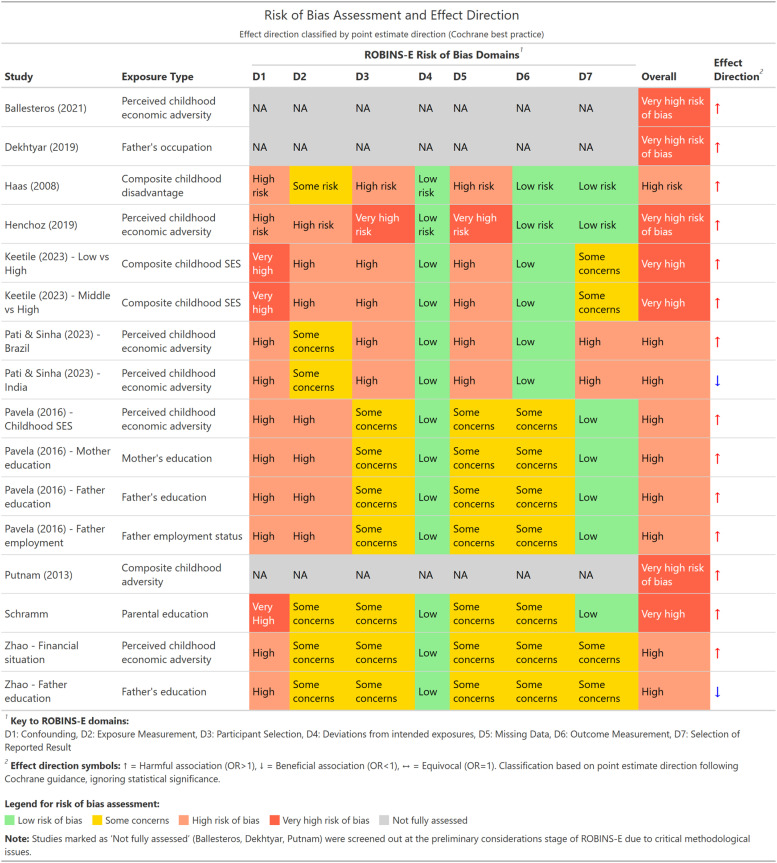
Risk of bias assessment and direction of effect for associations between childhood socioeconomic disadvantage and adult multimorbidity across included studies showing ROBINS-E domain ratings and effect direction (harmful, beneficial, equivocal) for each exposure–outcome association).

As shown in Figure 2, among the studies that underwent full domain assessment, the most common issues were inadequate confounding control, participant selection bias, and missing data concerns. Although most studies adjusted for critical confounders such as age, sex, and ethnicity, many failed to account for ideal confounders, particularly earlier socioeconomic circumstances, and some may have overadjusted by including mediators such as adult education or income. Selection bias was common, often arising from attrition and the lack of weighting to account for survey design or loss to follow-up. Most analyses relied on complete-case approaches, excluding participants with missing data. Deviations from intended exposures and outcome measurement were consistently rated as low risk across studies.

### Meta-analysis

#### Effect direction analysis

As demonstrated in Figure 2, across the 16 exposure–outcome combinations, effects were predominantly harmful (14 combinations), with only two beneficial associations observed. Using Cochrane best practice for effect direction classification (based on point estimate direction regardless of statistical significance), harmful associations (OR >1) were reported for perceived childhood economic adversity in most studies, parental education in most cases, and all composite measures. Only two exposure–outcome combinations demonstrated beneficial associations: Pati et al. (India cohort examining perceived childhood economic adversity and adult multimorbidity, APR = 0.76) and Zhao et al. (father’s education and adult multimorbidity in China, OR = 0.75). The predominance of harmful effects suggests a consistent directional trend toward increased multimorbidity risk associated with childhood socioeconomic disadvantage. However, the high or very high risk of bias ratings across all studies, reflecting various methodological challenges including limited confounding control, retrospective exposure measurement, and selection issues, limit confidence in the precision and magnitude of these associations.

#### Perceived childhood economic circumstances

**Figure 3.**
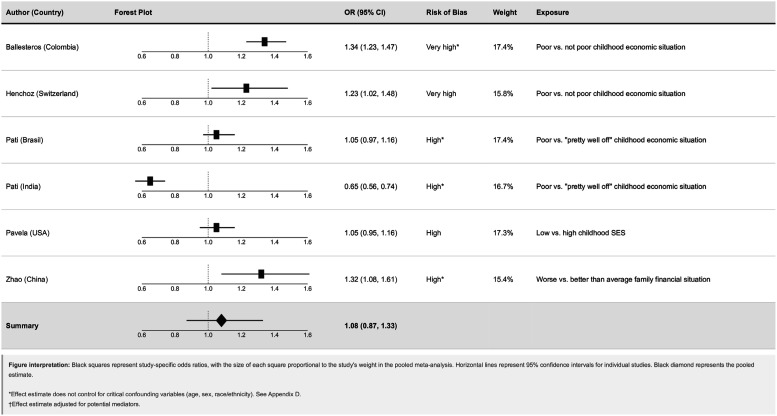
Forest plot of odds ratios for the association between perceived childhood socioeconomic adversity and adult multimorbidity, showing individual study estimates and pooled random-effects meta-analysis.

Six studies examining perceived childhood economic adversity were included in the meta-analysis (Figure 3). The pooled Odds ratio comparing adults with the most disadvantaged versus the most advantaged childhood economic circumstances was 1.08 (95% CI: 0.87, 1.23), providing no evidence of an association. Heterogeneity was substantial (I^2^ = 94.4%), indicating considerable variability in study results. Individual estimates differed in both magnitude and direction, with several confidence intervals crossing the null. Leave-one-out sensitivity analysis (see appendix J) demonstrated that exclusion of individual studies had modest effects on the pooled estimate, which ranged from 1.03 to 1.18 across all iterations. The largest change occurred when Pati et al. (India) was excluded, raising the pooled OR from 1.08 to 1.18 (95% CI: 1.05–1.32) and reducing heterogeneity from 94.7% to 77.2%. This suggests that the India cohort from Pati et al. may be acting as an outlier, contributing to both downward pressure on the pooled estimate and inflated heterogeneity. Exclusion of this study yielded a more precise estimate with a narrower confidence interval that more strongly suggested an increased Odds of multimorbidity associated with poor childhood economic circumstances.

#### Parental education

Three studies examined parental education, with separate analyses conducted for father’s and mother’s education, and one study using a combined parental education measure.

The pooled Odds ratio for multimorbidity comparing lower versus higher father’s education was 0.95 (95% CI: 0.66, 1.37), with moderate heterogeneity (I^2^ = 66.8%), indicating no clear association.

**Figure 4.**
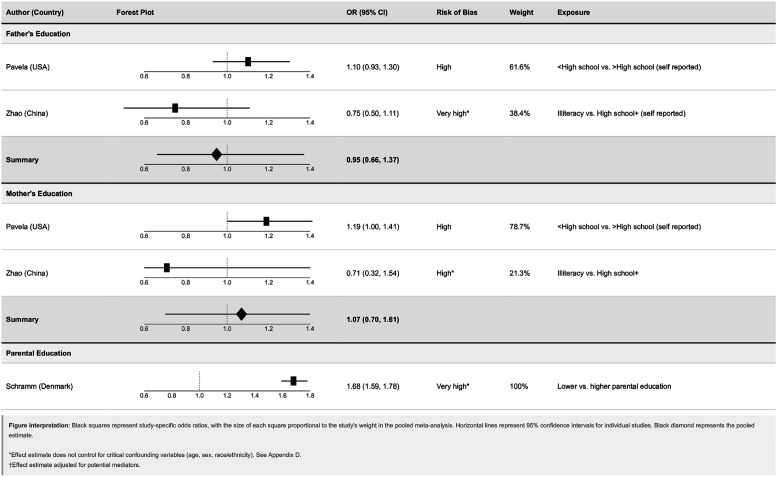
Forest plots of odds ratios for parental education and adult multimorbidity.

**Figure 5.**
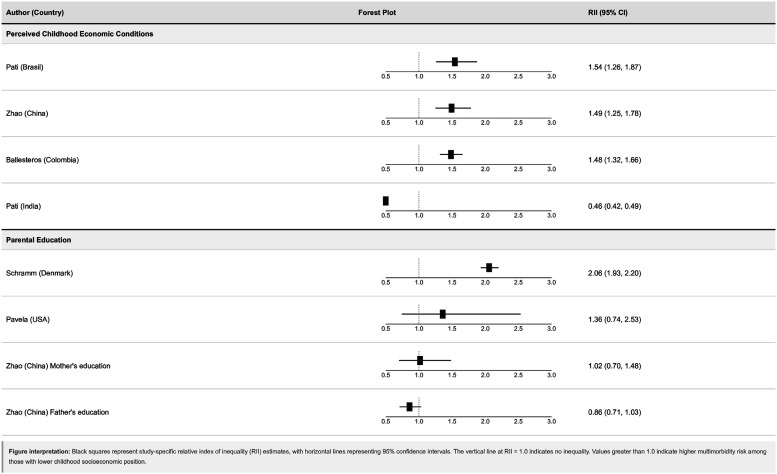
Forest plot of Relative Index of Inequality (RII) estimates for associations between childhood socioeconomic disadvantage and adult multimorbidity.

The pooled Odds ratio for mother’s education was 1.07 (95% CI: 0.70, 1.61), with lower heterogeneity (I^2^ = 36.9%). Pavela et al. reported a harmful association (OR: 1.19, 95% CI: 1.00, 1.41), while Zhao et al. reported a very imprecise result (OR: 0.71, 95% CI: 0.32, 1.54), leading to a pooled estimate that was imprecise. Therefore, there is no consistent evidence of association between parental education and multimorbidity (Figures 4 and 5).

The study by Schramm et al. was not meta-analysed with others due to conceptual differences in exposure measurement (combined parental education versus separate mother’s/father’s education). They reported the strongest individual association, comparing those with the lowest versus highest combined parental education (OR: 1.68, 95% CI: 1.59, 1.78).

#### Relative Index of Inequality analysis

Analysis of the Relative Index of Inequality (RII) provided insights into socioeconomic gradients across the full range of childhood disadvantage. The primary analysis included eight effect estimates from studies, with most demonstrating RII values greater than 1, indicating higher risk of adult multimorbidity among those most disadvantaged in childhood.

*Perceived childhood economic circumstances:* Three studies contributed RII estimates, all showing harmful associations with relatively precise estimates. RII values were 1.48 (95% CI: 1.32, 1.66) for Ballasteros et al., 1.49 (95% CI: 1.25–1.78) for Zhao et al., and 1.54 (95% CI: 1.26, 1.87) for Pati et al. (Brasil). A notable exception was Pati et al. (India), which reported a strongly protective association (RII = 0.46, 95% CI: 0.42, 0.49).

*Parental education:* Four RII estimates showed varied magnitudes but consistent direction. Schramm et al. reported the strongest association (RII = 2.06, 95% CI: 1.93, 2.20), while Pavela et al. showed a more modest effect (RII = 1.36, 95% CI: 0.74, 2.53) with wider confidence intervals. The Zhao studies examining father’s and mother’s education separately showed weaker but imprecise associations (RII = 0.86 and 1.02 respectively).

*Sensitivity analysis*: To assess whether inclusion of studies with insufficient groups for reliable gradient estimation would alter the overall pattern, we included six additional studies using less robust methods (2-group studies with single comparative effect estimate form which RII was extrapolated). These corroborated the primary findings, with Putnam et al. demonstrating a strong gradient (RII = 4.41, 95% CI: 3.23, 6.00) and the remaining studies showing consistent positive associations, with reported RII values ranging from 1.10 to 1.49.

Overall, RII estimates demonstrate consistent evidence for socioeconomic gradients despite varying effect magnitudes, with increased multimorbidity risk among those experiencing greater childhood disadvantage.

### Narrative synthesis of studies not included in meta-analysis

Three studies using composite exposure measures or otherwise incompatible outcome definitions could not be included in meta-analysis and were synthesised narratively.

#### Putnam et al. (2013) — United States

This cross-sectional study of 5,692 adults examined childhood economic adversity (hunger, lack of necessities, or government assistance) and mental health multimorbidity (disorders spanning ≥2 DSM-IV categories). A reconstructed unadjusted Odds ratio of 2.10 (95% CI: 1.80,2.45) was calculated from the original data. The study was rated as very high risk of bias primarily due to uncontrolled confounding, no adjustment for critical confounders including age, gender, race/ethnicity, or other childhood adversities. Additional concerns included potential selection bias from missing data exclusions, possible recall bias in retrospective exposure measurement, and some concerns regarding outcome measurement and result selection. While showing a substantial effect size, the unadjusted estimate and methodological limitations substantially reduce confidence in the findings.

#### Keetile et al. (2023) — Botswana

This cross-sectional study of 1,178 adults examined childhood socioeconomic status (derived from parental education, occupation, childhood stress, diet, perceived health, and major ailments) and multimorbidity. The study reported an adjusted Odds ratio of 1.78 (95% CI: 1.11, 2.68) for low versus high childhood SES. The study was rated as very high risk of bias primarily due to uncontrolled confounding. Additional high-risk domains included exposure measurement, participant selection, and missing data (unclear handling of missing data with likely complete-case analysis). While the effect estimate suggests a meaningful association, methodological limitations, particularly the very high risk of confounding bias, reduce confidence in the findings.

#### Haas (2008) — Multiple European countries

This cross-sectional study used data from multiple European countries, with sample sizes varying by country. A composite childhood socioeconomic measure combining parental education, occupation, and household economic resources was examined in relation to having two or more chronic conditions. The study reported a null association (OR: 0.98, 95% CI: 0.95, 1.01). While it had a relatively large sample size and precise estimate, it was rated as high risk of bias and provided limited detail about exposure construction and country-specific variations, making interpretation challenging.

### Synthesis of narrative findings

The three studies not included in meta-analysis demonstrated the heterogeneity that characterises this research area. Putnam et al. reported the strongest association (OR: 2.10, 95% CI: 1.80, 2.45) between childhood economic adversity and psychiatric multimorbidity, though this unadjusted estimate from a study with very high risk of bias examining mental rather than physical health conditions limits direct comparability with other studies. Keetile et al. provided evidence for a harmful association between low childhood SES and multimorbidity (OR: 1.78, 95% CI: 1.11, 2.68), although methodological limitations including uncontrolled confounding reduce confidence in these results. In contrast, Haas et al. reported a precise null association (OR: 0.98, 95% CI: 0.95, 1.01) across multiple European countries. While the strongest effects were observed in studies with greater methodological limitations, the pattern of results suggests that associations may exist but vary considerably across contexts and study designs.

### GRADE assessment

Evidence certainty was universally rated as very low across all exposure domains (Table 2). For perceived childhood economic circumstances, the pooled Odds ratio of 1.08 (95% CI: 0.87, 1.33) was downgraded for very serious risk of bias (all studies high/very high risk), very serious inconsistency (I^2^ = 94.4% with conflicting effect directions across settings), and extremely serious imprecision (confidence interval spanning from moderate protection to moderate harm).

**Table 2.** Summary of certainty of evidence (GRADE) for associations between childhood socioeconomic disadvantage and adult multimorbidity.

No. of studies	Certainty assessment						Effect			Certainty importance
	Study design	Risk of bias	Inconsistency	Indirectness	Imprecision	Other considerations	Pooled odds ratio (95% CI)	Risk ratio (95% CI)	Absolute effect per 1000 population (95% CI)	
Perceived socioeconomic circumstances in childhood										
5	non-randomised studies	very serious^a^	very serious^b^	Serious	extremely serious^c^	dose response gradient	1.08 (0.87 to 1.33)	1.05 (0.92 to 1.18)	19 (-30 to 68)	⨁◯◯◯
										Very low^a,b,c^
Father’s Education										
2	non-randomised studies	very serious^d^	serious^e^	serious^f^	extremely serious^g^	all plausible residual confounding would reduce the demonstrated effect dose response gradient	0.95 (0.66 to 1.37)	0.97 (0.76 to 1.20)	-11 (-91 to 76)	⨁◯◯◯
										Very low^d,e,f,g^
Mother’s education										
2	non-randomised studies	very serious^d^	not serious	serious^f^	extremely serious^h^	all plausible residual confounding would reduce the demonstrated effect dose response gradient	1.07 (0.70 to 1.61)	1.04 (0.79 to 1.31)	15 (-80 to 118)	⨁◯◯◯
										Very low^d,f,h^

**Explanations**

a. The studies by Ballasteros, Henchoz, Pati, and Zhao were rated as having very high (Ballasteros, Henchoz) or high (Pati, Zhao) risk of bias for this outcome, based on the ROBINS-E tool. The most common sources of bias included inadequate control for confounding, issues with participant selection, and missing data.

b. I^2^ can be unstable when few studies are included. Nonetheless, statistical heterogeneity was very high (I^2^ = 94.4%) for this pooled effect, indicating substantial variability in effect estimates across studies. Visual inspection confirms this concern: point estimates vary in direction and magnitude. Pati (India) suggests a strong protective association (OR 0.65), whereas Ballesteros (Colombia) and Zhao (China) report higher Odds of multimorbidity associated with perceived childhood economic hardship (ORs 1.34 and 1.32, respectively). Other studies, such as Henchoz (Switzerland) and Pavela (USA), suggest no important effect (ORs near 1). These results suggest that the true effect may differ markedly across settings. Therefore, we rated down the certainty of the evidence by two levels for very serious inconsistency.

c. The pooled Odds ratio was 1.08 (95% CI 0.87–1.23), with the point estimate indicating a trivial effect. However, the confidence interval spans from a moderate protective effect to a potentially large harmful effect. This interval crosses three predefined thresholds (trivial, moderate, and large), indicating substantial uncertainty about the true direction and magnitude of effect. Therefore, we rated down the certainty of evidence by three levels for extremely serious imprecision.

d. The certainty was downgraded for risk of bias because two of the contributing studies (Zhao and Pavela) were rated as being at very high and high risk of bias, respectively. The primary concern was inadequate control for confounding.

e. The I^2^ value for this pooled estimate was 66.8%, suggesting moderate to substantial statistical heterogeneity. While I^2^ can be inflated or imprecise with few studies, the magnitude of heterogeneity here aligns with visual and clinical assessment. The two included studies showed divergent point estimates: Zhao (China) reported an OR of 0.75, indicating a possible protective effect, whereas Pavela (USA) reported an OR of 1.10, suggesting a small harmful effect. These estimates point in different directions and imply differing possible underlying effects across populations. As this inconsistency could not be explained by a small number of pre-specified subgroup hypotheses, we rated down the certainty of the evidence by one level for serious inconsistency.

f. The certainty was downgraded for indirectness because both of the contributing studies drew on data for older adults. (Zhao et al.) used the China Health and Retirement Longitudinal Study (CHARLS), which focuses exclusively on adults aged 60 and over (mean age of study population 68.25 years), and Pavela et al. use data from the Health and retirement study (mean age 54.9 years). This limits generalisability to an overall adult population which is the stated population of interest of the present systematic review.

g. The pooled Odds ratio was 0.95 (95% CI 0.66–1.37). The point estimate lies within the trivial range; however, the 95% confidence interval spans from a potentially large protective effect (<0.80) to a large harmful effect (>1.25). This confidence interval crosses multiple predefined thresholds for effect size (trivial, moderate, and large), reflecting considerable uncertainty about the true direction and magnitude of the effect. As a result, we rated down the certainty of the evidence by three levels for extremely serious imprecision.

h. The pooled Odds ratio was 1.07 (95% CI 0.70–1.61). While the point estimate suggests a small harmful effect, the confidence interval ranges from a large protective effect to a large harmful effect, crossing multiple GRADE-defined thresholds (trivial, moderate, and large). This wide interval reflects substantial uncertainty in the estimated effect size. Therefore, we rated down the certainty of the evidence by three levels for extremely serious imprecision.

For parental education, both father’s and mother’s education showed very low certainty evidence. Father’s education (OR: 0.95, 95% CI: 0.66–1.37) was downgraded for very serious risk of bias, serious inconsistency (I^2^ = 66.8% with opposing effect directions), serious indirectness (studies limited to older adult populations), and extremely serious imprecision. Mother’s education (OR: 1.07, 95% CI: 0.70–1.61) received similar downgrades for very serious risk of bias, serious indirectness, and extremely serious imprecision, while inconsistency was rated as not serious.

### Absolute risk differences

Absolute risk differences were small and uncertain across all exposures: 7 additional cases per 1000 for perceived economic circumstances (95% CI: -42 to 57), 24 fewer cases per 1000 for father’s education (95% CI: -91 to 76), and 22 additional cases per 1000 for mother’s education (95% CI: -79 to 115). The wide confidence intervals crossing multiple effect size thresholds (trivial, moderate, large) reflect substantial uncertainty about both the direction and magnitude of any potential associations. Overall, the certainty of evidence was rated very low across all exposure domains.

### Subgroup analyses

Subgroup analyses were conducted to explore potential sources of heterogeneity (see Appendix K).

#### Geographic region

For perceived childhood economic circumstances, pooled estimates showed no clear associations across regions. Latin America showed the highest point estimate (OR 1.19, 95% CI: 0.94–1.50; I^2^ = 92.8%), with substantial heterogeneity between the Colombian and Brazilian studies. Asia showed a null association (OR 0.93, 95% CI: 0.46–1.84; I^2^ = 96.9%), reflecting high heterogeneity between the protective association in India and the harmful association in China. Single studies from Europe (Switzerland) and North America (USA) both reported null associations.

#### Study design

Cross-sectional studies of perceived childhood economic circumstances showed no clear association (OR 1.03, 95% CI: 0.79–1.34; I^2^ = 94.8%), with substantial heterogeneity across studies. The single longitudinal study (Pavela) also showed a null association (OR 1.05, 95% CI: 0.95–1.16).

#### Multimorbidity definition

Studies using the standard definition of ≥2 chronic conditions showed no clear association (OR 1.03, 95% CI: 0.79–1.34; I^2^ = 94.8%), and the single study using a continuous measure of chronic conditions (Pavela) reported a similar null finding.

#### Exposure type comparison

When comparing all exposure types, no exposure category showed a consistent association with multimorbidity. Perceived childhood economic circumstances were examined in the largest number of studies, but the pooled estimate indicated no clear association with adult multimorbidity (six studies; pooled OR = 1.03, 95% CI 0.84–1.28; I^2^ = 93.5%), suggesting substantial heterogeneity and an overall null effect. Father’s and mother’s education both showed null associations with moderate-to-high heterogeneity. The Danish study of combined parental education (Schramm) remained the strongest individual association observed across all analyses (OR 1.68, 95% CI: 1.59–1.78), but could not be pooled due to differences in exposure definition.

The subgroup analyses yielded consistently null findings across all subgroups, with substantial heterogeneity persisting within most categories. This suggests that the observed heterogeneity in the overall meta-analysis may be driven more by methodological differences across studies than by true subgroup effects.

## Discussion

This review provides the first comprehensive synthesis of evidence on childhood socioeconomic circumstances and adult multimorbidity, revealing a critically limited evidence base characterised by few studies with high risk of bias and substantial heterogeneity. Only ten studies met inclusion criteria from over 5,600 records, with all rated as high or very high risk of bias, most commonly due to inadequate confounding control and reliance on retrospective self-report. While most studies adjusted for basic demographic factors such as age, sex, and ethnicity, few accounted for key antecedent factors like parental health or prior socioeconomic conditions which may have influenced both exposure and outcome. The evidence was further constrained by predominantly cross-sectional designs, which limited the ability to establish temporal order between childhood exposures and adult outcomes, alongside reliance on older cohorts, substantial variation in multimorbidity definitions, and universally very low certainty ratings using GRADE methodology.

Within these methodological constraints, the review found evidence potentially consistent for harmful associations between childhood socioeconomic disadvantage and adult multimorbidity, though with substantial uncertainty. Pooled estimates consistently indicated increased harm across exposure domains but confidence intervals were wide and often encompassed both protective and harmful effects. While the vast majority of studies indicated harm, heterogeneity reflected variation in the magnitude of effects across contexts and populations. Leave-one-out sensitivity analysis revealed that individual studies influenced pooled estimates. Excluding the Pati (India) study, which showed a protective association contrary to other studies, strengthened the evidence for harm, with the pooled estimate for perceived economic circumstances showing improved precision with confidence intervals that no longer encompassed the null, and heterogeneity decreasing markedly. This suggests the association may be stronger and more consistent than initial pooled estimates indicated, though the influence of individual studies highlights the fragility of conclusions. RII analyses provided support for socioeconomic gradients in multimorbidity risk, with most estimates exceeding 1.0 and ranging from modest (RII 1.10–1.54) to substantial effects (RII 2.06–4.41). However, consistency and precision varied considerably, with findings particularly heterogeneous for parental education measures. While these gradient analyses suggested possible dose-response relationships, the evidence remains inconclusive given the universal high risk of bias, reliance on cross-sectional designs, and substantial between-study heterogeneity in exposure measurement and population characteristics.

Our findings present a more nuanced picture than might be expected given the well-established social gradient in health outcomes. While a large body of literature has consistently demonstrated associations between socioeconomic position and various health outcomes, including mortality, single disease risk, and disability, our review suggests that the relationship with multimorbidity may be more complex and context-dependent. This inconsistency likely reflects multiple factors.

Critically, the very limited evidence base identified in this review may itself create misleading conclusions about this relationship. The apparent absence of consistent associations may reflect fundamental gaps in research rather than genuine absence of effects, highlighting how insufficient evidence can obscure understanding of potentially important associations.

Secondly, multimorbidity itself is an evolving and heterogeneous construct, encompassing diverse combinations of conditions that differ in severity, impact, and social patterning; such combinations are not necessarily equivalent or commutative. This complexity can obscure underlying gradients. Although the epidemiological transition model has been critiqued for its imprecision, it remains a useful lens for understanding how patterns of disease vary across settings.^34–36^ The studies included in this review span contexts at different stages of this epidemiological transition, from high-income countries like Denmark and the USA, where chronic disease dominates, to countries such as India, Colombia, and Botswana, where infectious and non-communicable disease burdens may coexist. In some lower- and middle-income country settings, certain chronic conditions may paradoxically be associated with socioeconomic advantage due to differential access to healthcare for diagnosis, lifestyle factors associated with economic development, or survival bias where only the more advantaged populations live long enough to develop chronic conditions. These differences likely shape both the composition of multimorbidity and the strength of association with childhood disadvantage, contributing to the observed heterogeneity. Additionally, the influence of childhood socioeconomic disadvantage on health may vary across social, cultural, and healthcare contexts, further complicating comparisons.

Methodological limitations, including retrospective exposure measurement and inconsistent multimorbidity definitions, also constrain both internal validity and cross-study comparability. Moreover, different definitions of multimorbidity may themselves contribute to the heterogeneity in observed effect directions. Depending on which conditions are included—and how these conditions are socially patterned—studies may capture different underlying mechanisms. For instance, a definition weighted towards mental health and musculoskeletal conditions may show stronger socioeconomic associations than one focused on conditions with more stochastic or genetically driven components. This definitional variability can mask consistent underlying pathways or exaggerate contextual differences.

Life course processes may also attenuate or modify the effects of early-life disadvantage, with adult socioeconomic position, health behaviours, and healthcare access shaping multimorbidity risk over time. For example, individuals from disadvantaged childhoods who achieve higher education or income in adulthood may experience reduced risk compared to those who remain disadvantaged throughout life. In addition, competing risks must be considered. Individuals from more disadvantaged backgrounds may face higher mortality from single conditions before reaching the age at which multimorbidity typically emerges, introducing selective survival bias that could lead to underestimation of associations. The inconsistency between our pooled estimates and the predominance of positive Relative Index of Inequality (RII) values further suggests that while socioeconomic gradients in multimorbidity may exist, their magnitude is highly variable across populations and settings, potentially reflecting differences in how socioeconomic circumstances are categorised and measured across studies as well as genuine population differences in the strength of socioeconomic health gradients.

### Strengths and limitations

This review has several strengths. It provides a comprehensive synthesis of the available evidence, incorporating multiple exposure types and studies from diverse international contexts. A structured risk of bias assessment (ROBINS-E) was applied systematically, and the certainty of evidence was transparently assessed using GRADE. The inclusion of both effect direction synthesis and, where feasible, meta-analysed Relative Index of Inequality (RII) values enabled a more nuanced interpretation of the evidence than meta-analysis alone would allow.

However, the review also has important limitations. The narrowness of both the exposure and outcome definitions meant that some relevant studies such as some, using broader childhood adversity constructs (e.g. Haapanen)^37^ or alternative multimorbidity definitions (e.g. Khanolkar),^24^ were excluded, resulting in the loss of potentially informative data. Our strict operationalisation of multimorbidity (≥2 chronic conditions) provided analytical precision but excluded studies with valuable information on disease trajectories and clustering patterns. Notably, both excluded studies reported that early-life or childhood socioeconomic disadvantage was associated with faster accumulation of chronic conditions across adulthood, consistent with the direction of associations observed in this review. The number of eligible studies was small, and the meta-analyses were based on limited data of uncertain reliability. The small number of studies contributing to each meta-analysis limits the robustness of pooled estimates. With as few as two datapoints in some analyses, statistical heterogeneity is likely to be inflated or unreliable, and precision estimates must be interpreted with caution. This reinforces the tentative nature of any pooled findings. Many included studies were cross-sectional, increasing susceptibility to recall and survival bias. Substantial heterogeneity in exposure definitions, particularly for composite measures, precluded meta-analysis in some domains. The majority of included studies were at high or very high risk of bias, largely due to poor confounding control and the use of selective or retrospective samples. Publication and reporting bias may also have influenced the findings, potentially leading to underrepresentation of null or negative results. Finally, exposure data are, by necessity, retrospective and reflect past socioeconomic conditions that may not capture the mechanisms shaping multimorbidity in today’s populations.

To mitigate these limitations, we stratified analyses by exposure domain, applied a structured framework to prioritise overlapping data sources, and conducted sensitivity analyses where possible. These measures aimed to maximise transparency and robustness within the constraints of the available evidence.

### Contribution, implications, and future directions

This review offers the first systematic synthesis of evidence on the relationship between childhood socioeconomic circumstances and adult multimorbidity. In doing so, it highlights key methodological challenges specific to this field, particularly in the retrospective measurement and conceptualisation of childhood exposures and the evolving, heterogeneous definitions of multimorbidity. It also demonstrates substantial contextual variability in observed associations. By applying GRADE methodology and compiling Relative Index of Inequality (RII) estimates, the review provides a transparent appraisal of the evidence base and a standardised means of comparing effect estimates across settings. These contributions lay the groundwork for more rigorous, comparative, and context-sensitive research into the life course origins of multimorbidity. Although plausible pathways have been proposed linking childhood socioeconomic disadvantage to adult multimorbidity, current evidence remains inconsistent and uncertain. This should not be interpreted as evidence of no effect but highlights the need for more rigorous longitudinal research with appropriate adjustment for confounding. Given the apparent context-specific nature of associations and likely life course modification pathways, policies should adopt a life course perspective and be tailored to local contexts. Improving the quality of evidence in this area should remain a priority.

Given the ethical and practical constraints of interventional studies in this domain, future research should focus on applying robust causal inference methods to longitudinal and routinely collected observational data. Advanced analytical approaches can help strengthen causal interpretation in specific circumstances. Future studies should prioritise longitudinal designs with robust confounder control, including parental health and other early-life factors, and ensure clear temporal ordering of exposures and outcomes. Advanced causal inference methods, such as marginal structural models or instrumental variable approaches, may also help to address time-varying and unmeasured confounding. However, given the current uncertainty around the exposure–outcome association, efforts should first focus on improving study quality and causal identification before examining mechanistic pathways. Progress also requires greater standardisation in how both childhood socio-economic circumstances and multimorbidity are measured and improved representation of diverse global populations.

## Conclusion

This review finds suggestive but highly uncertain evidence for harmful associations between childhood socioeconomic disadvantage and adult multimorbidity, with evidence marked by substantial heterogeneity. While socioeconomic gradients appear to exist in most contexts, current data are insufficient to draw firm conclusions about their magnitude or consistency. Future research should prioritise robust causal methods, longitudinal designs, and standardised measures to better understand these life course relationships.

## Supplemental material

Supplemental material - Childhood socioeconomic disadvantage and adult multimorbidity: A systematic review and meta-analysis Supplemental material for Childhood socioeconomic disadvantage and adult multimorbidity: A systematic review and meta-analysis by Philip Broadbent, Alistair Carr, Erik Igelström, Valerie Wells, Anna Pearce, Daniel Kopasker, and Srinivasa Vittal Katikireddi in Journal of Multimorbidity and Comorbidity.

## Data Availability

The datasets analysed in this study were derived from previously published studies. All data used in the review are available within the published articles included in the systematic review. Analysis code is provided in Appendix N.*
