# Supplementary material for: Childhood socioeconomic disadvantage and adult multimorbidity: A systematic review and meta-analysis
Source: J Multimorb Comorb. 2026 Apr 29;16:26335565261447702. doi: 10.1177/26335565261447702 (PMC13145020; doi:10.1177/26335565261447702)
Supplement: Supplemental material - Childhood socioeconomic disadvantage and adult multimorbidity: A systematic review and meta-analysis [file sj-pdf-1-cob-10.1177_26335565261447702.pdf]

Appendices

Contents

Appendices 1

Appendix A – Search strategy 2

Appendix B 9

Appendix C: Framework for prioritising studies with shared or overlapping data sources 3

Appendix D – Data extraction form 12

- Study Details 12
- Exposure Details 12
- Outcome Details 12
- ROBINS-E Outcomes 12

Appendix E: Risk of Bias and Causal Framework 14

- i. Target trial emulation for the impact of socioeconomic circumstances in childhood on multimorbidity in adulthood 14

Appendix F: Study-Specific methodological notes and effect estimate selection 26

Appendix G - Clarifications and amendments to systematic review protocol 34

Appendix H: Estimating the Relative Index of Inequality (RII) 35

Appendix I: RII estimates and analysis 39

Appendix J: Leave-one-out sensitivity analysis 45

Appendix K: Subgroup analysis 47

Appendix L: Defining effect size thresholds for certainty judgements using the GRADE Evidence to Decision Framework 56

Appendix M – Absolute Risk Difference Calculations for GRADE 58

Appendix N - R code used to generate plots 59

- Forest plot code 59
- Leave one out plot 63
- RII Code 66
- Effect direction and Risk of Bias plot 70
- Subgroup analysis 73

Appendix O – Full text screening outcomes and exclusion reasons 78

- References 94

Appendix A – Search strategy

The following strategy was conducted in *Ovid MEDLINE* (1946 to March 2025). Equivalent searches were adapted for Embase, Scopus, Web of Science, and PsycINFO.

Table A1: Search strategy

Concept	Search Terms (Ovid MEDLINE Syntax)
**1. Population**	child*.tw,kw. OR infant*.tw,kw. OR young*.tw,kw. OR early-life.tw,kw.
**2. Exposure: Socioeconomic disadvantage**	- exp Socioeconomic Factors/ - (SES or SEP or sociodemographic* or socio-demographic* or income or wealth* or poverty or educational level or level of education or educational attainment or well educated or better educated or unemploy* or home owner* or tenure or affluen* or well off or better off or worse off).tw,kw. - (socioeconomic* or inequalit* or disadvantage* or SEP or SES or inequity or disparit* or equit* or inequit* or equality).ab,ti. - (poverty or deprivation or adversity or underprivileged or poor or hardship or free school meals or disadvantaged).ab,ti.
**3. Outcome: Multimorbidity and chronic illness**	- exp Multimorbidity/ - multi-morbid*.ab,ti. OR co-morbid*.ab,ti. OR multimorbid*.ab,ti. OR comorbid*.ab,ti. - (multiple adj2 (chronic or long-term or long term) adj2 (condition* or disease* or disorder* or illness*)).ab,ti. - (chronic adj2 (illness or disease* or condition*)).ab,ti. - polypatholog*.ab,ti. - (health adj1 (ill or poor)).ab,ti. - exp Health Status/
**4. Combined search**	1 AND 2 AND 3

Appendix B: Framework for prioritising studies with shared or overlapping data sources

To ensure we avoided including duplicate data, we applied a set of criteria aimed at identifying the study that both best aligned with our PECOS framework and presented the lowest overall risk of bias. The following principles guided our decision-making:

Primary criteria (treated equally):

- ROBINS-E risk of bias judgement
- Effect estimate amenable to meta-analysis (convertible to some sort of quantitative comparative effect estimate e.g. hazard ratio and provides precision estimates)
- *Sample size:* Preference was given to studies with larger analytic samples.
- *Study design:* Longitudinal analyses were favoured over cross-sectional ones.
- *Relevance to review question:*
    - Did the study population match ours in terms of inclusion criteria?
    - Were the exposure(s) and outcome(s) comparable to our definitions?
- *Handling of confounding*: Studies that employed stronger approaches to controlling for confounding (either through design or analysis) were prioritised.

Secondary criteria (used when primary criteria could not separate studies):

- *Recency of data:* Preference was given to studies using more up-to-date data.
- *Duration of follow-up:* For longitudinal studies, those with longer follow-up periods were prioritised.

We applied this decision-making framework to address overlapping data sources in the following cases: (1) Haas and Jungo, both of which used data from the Survey of Health, Ageing and Retirement in Europe (SHARE), with overlapping time periods and countries (noting that Haas additionally included ELSA and TILDA, while Jungo relied solely on SHARE); and (2) Goosby (2013) and Putnam (2013), both of which drew on the National Comorbidity Survey Replication (NCS-R) dataset. (3) Pavela and Kwon used overlapping data from the health and retirement study (HRS). (4) Both Jin et al. and Zhao et al. use China Health and Retirement Longitudinal Study (CHARLS), specifically the 2014 Life History module and 2015 Wave 3 data

Haas and Jungo

Criterion	Haas	Jungo	Decision
**Sample size**	36,459	31,432	Haas
**Study design**	Longitudinal retrospective cohort	Longitudinal retrospective cohort	Equal
**Population match (PECOS)**	Adults across Europe; no upper age restriction	Focuses on older adults (65+), limiting generalisability to broader adult population	Haas
**Exposure**	Childhood socioeconomic conditions (e.g. parental education, financial hardship)	Childhood socioeconomic adversity	Equal
**Outcome**	Multimorbidity defined as 2+ chronic conditions (matches review definition)	Multimorbidity included, but polypharmacy and other outcomes complicate relevance	Haas
**Confounding approach**	Adjusts for age, sex, and birth cohort, does not control for adult SES	Adjusts for adult SES, which lies on the causal pathway (risk of overadjustment bias)	Haas
**Effect estimate usability**	ORs with 95% CI presented in meta-analysable format	Adjusted RRs stratified by subgroup; less extractable for meta-analysis	Haas
**Peer-reviewed**	Yes	Yes	Equal
**Recency of data**	SHARE Waves up to ~2012/2014	SHARE Waves up to ~2017 (Wave 7)	Jungo
**Follow-up duration**	Life-course via SHARE retrospective data	Life-course via SHARE retrospective data	Equal
**Risk of Bias**	High Risk	Very High Risk	Haas

Haas and Jungo both use data from the Survey of Health, Ageing and Retirement in Europe (SHARE) to examine associations between childhood socioeconomic conditions and adult health outcomes, covering overlapping time periods and European populations. However, Haas was prioritised for inclusion in the review following comparison based on relevance to the PECOS framework and methodological suitability. Both studies employed retrospective cohort designs using life-course data and had similar sample sizes (36,459 vs. 31,432). SHARE includes individuals aged 50 and above. Jungo explicitly restricts the analytic sample to those aged 65 and older, whereas Haas includes a broader segment of the SHARE cohort starting at age 50. Both studies assessed similar childhood exposures, but Haas more directly aligned with the review outcome definition, explicitly

operationalising multimorbidity as the presence of two or more chronic conditions. Jungo included multimorbidity among several outcomes but incorporated polypharmacy and stratified analyses that complicated extractability. Haas also presented effect estimates as odds ratios with 95% confidence intervals suitable for meta-analysis, while Jungo reported adjusted risk ratios stratified by subgroups. Haas adjusted for key demographic confounders (age, sex, birth cohort) without controlling for adult socioeconomic status, avoiding potential overadjustment along the causal pathway, whereas Jungo included adult SES as a covariate. While Jungo did use slightly more recent SHARE data (up to Wave 7), on balance we felt that Haas had greater alignment with our inclusion criteria and analytic requirements, and lower overall risk of bias.

Goosby & Putnam

**Criterion**	**Putnam**	**Goosby**	**Decision**
**Sample size**	~9,282 (NCS-R Part II sample)	~6,000 (subset of NCS-R Part II focusing on adults aged 25–64)	Putnam
**Study design**	Cross-sectional	Cross-sectional	Equal
**Population match (PECOS)**	Adults 18+ from national U.S. sample	Adults aged 25–64, excludes younger and older adults	Putnam
**Exposure**	Childhood economic adversity (composite of public assistance, food/housing hardship)	Childhood SES and adversity (includes family structure, violence, financial strain, etc.)	Equal
**Outcome**	Counts of DSM-IV disorders across four domains (mood, anxiety, impulse, substance); 2+ used as MM proxy	Only depression and chronic pain, analysed separately; not clearly defined as multimorbidity	Putnam
**Confounding approach**	Unadjusted logistic regression (crude ORs)	Appropriate adjustment	Putnam
**Effect estimate usability**	Replicable from public NCS-R data; crude ORs reconstructable and meta-analysable	Adjusted RRRs; stratified models	Putnam
**Peer-reviewed**	Yes	Yes	Equal
**Recency of data**	NCS-R collected 2001–2003	Same dataset	Equal
**Follow-up duration**	None (cross-sectional)	None (cross-sectional)	Equal
**Risk of bias**	Very High Risk	Some concerns	Goosby

Putnam (2013) and Goosby (2013) used data from the National Comorbidity Survey Replication (NCS-R), specifically from Part II of the dataset. Putnam's analysis included the full NCS-R Part II sample (~9,282 adults aged 18 and older), whereas Goosby restricted the sample to adults aged 25–64 (~6,000), thereby excluding younger and older adults. Both studies employed a cross-sectional design, and exposures were broadly comparable: both measured aspects of childhood socioeconomic adversity, though operationalised slightly differently. However, Putnam was prioritised due to a closer alignment with our outcome definition. Putnam analysed multimorbidity as a count of DSM-IV psychiatric disorders (across four domains: mood, anxiety, impulse, and substance disorders), with 2+ disorders used as a proxy for multimorbidity. In contrast, the relevant outcome modelled in the Goosby paper was depression and chronic pain in combination. While Goosby's models included appropriate adjustment for key confounders, we extracted unadjusted odds ratios using data from the Putnam study, which may be more susceptible to bias but were readily extractable and replicable from public NCS-R data. Goosby was judged to have some concerns due to appropriate confounder control but less clear outcome relevance, while Putnam was rated as very high risk due to lack of adjustment. On balance, Putnam's broader population and suitability for meta-analysis led us to prioritise the effect estimate from this study over Goosby for inclusion, despite its higher risk of bias.

Pavela and Kwon

**Criterion**	**Kwon et al.**	**Pavela et al.**	**Preferred**
**Sample size**	9,056 adults aged 51–64 (50,726 observations)	10,584 adults (Waves 1–9, 1992–2008)	Equal
**Study design**	Longitudinal (1998–2010), growth curve modelling	Longitudinal (1992–2008), pooled logistic regression	Equal
**Population match (PECOS)**	Middle-aged adults (51–64), U.S. population	Adults aged 51+, U.S. population	Pavela
**Exposure**	Childhood economic hardship (financial strain, parental education, parental work)	Composite childhood SES (parental education, family received help, perceived financial status)	Equal
**Outcome**	Multiple chronic conditions (counts, including 2+ as threshold)	Number of chronic conditions (2+ used as a categorical outcome in logistic model)	Pavela
**Confounding approach**	Controls for childhood and adult characteristics (but includes adult SES potential overadjustment)	Models vary; Odds ratios pertaining to MM control for race, nativity, sex, and adult SES.	Equal
**Effect estimate**	Provides regression coefficients from growth curve models (not	Provides odds ratios and 95% CIs for multimorbidity categories —	Pavela

**usability**	odds ratios)	directly usable in meta-analysis	
**Peer-reviewed**	Yes	Yes	Equal
**Recency of data**	HRS 1998–2010	HRS 1992–2008	Kwon
**Follow-up duration**	~12 years	~16 years	Pavela
**Risk of Bias**	High	High	Equal

Summary:

Kwon et al. and Pavela et al. draw on data from the Health and Retirement Study (HRS) and use overlapping waves. We included Pavela et al. in our review due to several factors that made it more closely aligned with our PECOS criteria and more suitable for meta-analysis. While both studies are longitudinal and peer-reviewed, Pavela's sample included a broader age range (adults aged 51+), compared to Kwon's focus on adults aged 51–64. Both studies assessed early-life socioeconomic conditions using comparable constructs, and both defined multimorbidity as two or more chronic conditions. However, Pavela clearly reported odds ratios and 95% confidence intervals for categories of multimorbidity, which aligned directly with our analytic approach. In contrast, Kwon presented regression coefficients from growth curve models, which were not readily convertible for use in our meta-analysis. Both Kwon and Pavela adjusted for adult SES, raising concerns about overadjustment since adult SES may lie on the causal pathway. Both studies were judged to be at high risk of bias, due primarily to potential overadjustment via inclusion of adult SES. Despite Kwon using more recent data (through 2010), Pavela's study offered a longer follow-up period and clearer outcome presentation. On balance, Pavela was the stronger fit for inclusion, with both studies being rated at high risk of bias.

Zhao and Jin

Criterion	Jin	Zhao	Decision
**Data source**	CHARLS 2014 Life History + 2015 Wave 3	CHARLS 2014 Life History + 2015 Wave 3	Likely overlap
**Sample size**	5,735 adults aged ≥60	7,578 adults aged ≥60	Zhao
**Population**	Adults 60+	Adults 60+	Equal
**Exposure**	Index comprising of Highest education level of mother/father, self-perceived houshold financial situation up to 17, and not enough food to eat up to17. Also self-perceived financial situation and highest parental education level	Biological father's education level (Illiteracy/Below high school diploma, high school diploma or above), Biological mother's education level (Illiteracy/Below high school diploma, high school diploma or above), family financial	Zhao

	analysed on as single factors.	situation (Worse off than an average family/ Same as an average family/ Better off than an average family)	
**Outcome**	Multimorbidity (2+ chronic conditions)	Multimorbidity (2+ chronic conditions)	Equal
**Confounding**	Age, sex, adult SES, lifestyle	Age, gender, SES, behaviours, BMI	Zhao
**Effect estimate usability**	ORs reported, meta-analysable	ORs reported, meta-analysable	Equal
**Peer-reviewed**	Yes	Yes	Equal
**Risk of bias**	Very High	High	Zhao
**Conclusion**	Both use same CHARLS wave and module; analysis questions differ slightly but sample overlap is almost certain.		Zhao

Summary:

Both Jin et al. and Zhao et al. use China Health and Retirement Longitudinal Study (CHARLS), specifically the 2014 Life History module and 2015 Wave 3 data. Despite differences in analytic focus, the similarity in data sources, populations (adults aged 60 and above), and exposure-outcome pairings suggests near-complete sample overlap. Zhao has a slightly larger analytic sample (7,578 vs. 5,735). While both studies assessed childhood socioeconomic adversity and reported multimorbidity outcomes as having two or more chronic conditions, Zhao's operationalisation of exposure was more closely aligned with our systematic review intention (parental education, perceived economic conditions in childhood), whereas Jin used a composite measure including elements that we had elected to exclude (e.g. food insecurity). Both Zhao and Jin omitted control for ethnicity and both adjusted for adult SES. Zhao was assessed to be at high risk of bias due to omission of key confounders (e.g. ethnicity), but Jin was rated very high risk owing to use of less well-specified exposure constructs and limited confounding control. Both studies presented odds ratios with confidence intervals in formats suitable for meta-analysis. However, given Zhao's exposure specification, larger sample size, and comparable methodological transparency, it was selected for inclusion over Jin

Appendix C

In this review, included studies were required to operationalise multimorbidity, or to report an outcome reflecting the presence of two or more chronic conditions from a predefined list of recognised long-term disorders. This criterion was based on the definitions proposed by Barnett et al. (2012)[1] and Ho et al. (2022)[2], which conceptualise multimorbidity as the coexistence of two or more long-term conditions that are chronic, permanent, or relapsing in nature and require ongoing treatment, care, or surveillance. Conditions were considered relevant if included in either source.

Studies that only reported a simple count of conditions (for example, the number of diagnoses per participant) without a categorical outcome variable indicating multimorbidity or from which multimorbidity could be derived were excluded.

Table B1. Conditions included in Multimorbidity definition

Condition	Barnett et al. (2012)	Ho et al. (2022)
Hypertension (treated/untreated)	Y	Y
Depression	Y	Y
Painful condition / chronic primary pain	Y	Y
Asthma (currently treated)	Y	Y
Coronary heart disease	Y	Y
Treated dyspepsia / peptic ulcer	Y	Y
Diabetes	Y	Y
Thyroid disorder	Y	Y
Rheumatoid arthritis / inflammatory polyarthropathies / connective tissue disorders / osteoarthritis	Y	Y
Hearing loss / impairment (uncorrectable)	Y	Y
Chronic obstructive pulmonary disease	Y	Y
Anxiety and other neurotic, stress-related and somatoform disorders	Y	Y
Irritable bowel syndrome	Y	N
Cancer (any type, including solid organ, haematological, metastatic, melanoma, or benign cerebral tumours)	Y	Y
Alcohol problems / misuse	Y	Y

Other psychoactive substance misuse	Y	Y
Treated constipation	Y	N
Stroke and transient ischaemic attack	Y	Y
Chronic kidney / end-stage kidney disease	Y	Y
Diverticular disease of intestine	Y	N
Atrial fibrillation / arrhythmia	Y	Y
Peripheral vascular disease	Y	Y
Heart failure	Y	Y
Prostate disorders	Y	N
Glaucoma	Y	N
Epilepsy (treated)	Y	Y
Dementia	Y	Y
Schizophrenia / bipolar disorder	Y	Y
Psoriasis / eczema	Y	N
Inflammatory bowel disease	Y	Y
Migraine	Y	N
Blindness / low vision (uncorrectable)	Y	Y
Chronic sinusitis	Y	N
Learning disability	Y	N
Anorexia or bulimia / eating disorder	Y	Y
Bronchiectasis	Y	Y
Parkinson’s disease	Y	Y
Multiple sclerosis	Y	Y
Viral hepatitis	Y	N
Chronic liver disease	Y	Y
Heart valve disorders	N	Y

Venous thromboembolic disease	N	Y
Aneurysm	N	Y
Addison’s disease	N	Y
Cystic fibrosis	N	Y
Paralysis	N	Y
Peripheral neuropathy	N	Y
Autism spectrum disorder	N	Y
Post-traumatic stress disorder	N	Y
Long-term musculoskeletal problems due to injury	N	Y
Osteoporosis	N	Y
Gout	N	Y
Chronic pancreatic disease	N	Y
Endometriosis	N	Y
Chronic urinary tract infection	N	Y
Anaemia (including pernicious and sickle-cell anaemia)	N	Y
Ménière’s disease	N	Y
HIV / AIDS	N	Y
Chronic Lyme disease	N	Y
Tuberculosis	N	Y
Post-acute COVID-19	N	Y
Congenital disease and chromosomal abnormalities	N	Y

- Conditions were included if listed in either *Barnett et al.* (2012) or *Ho et al.* (2022).
- “Y” indicates inclusion in the condition list of the respective study.
- Studies defining multimorbidity as ≥ 2 of these conditions were eligible for inclusion, even if not explicitly labelled as measuring “multimorbidity.”

Appendix D – Data extraction form

Study Details

First Author & Year	
Participant Cohort	
Cohort Country	
Years of Recruitment	
Study Type (cross-sectional, longitudinal)	
Follow-up years (if applicable)	
Sample Size	
Female Gender (%)	
Age (Range, Mean, SD)	
Comments	

Exposure Details

First Author & Year	
Childhood SEC Exposure	
Exposure Definition	
Measurement Tool for Exposure	
Childhood Upper Age Limit	
Critical Period or Timing of Exposure	
List of Socioeconomic Adversities	
Prevalence of SE Adversity	

Outcome Details

First Author & Year	
Was multimorbidity included as an outcome by the study authors? (Yes/No)	
If yes, what was the multimorbidity definition used?	
If no, what were the outcomes relevant to our research question?	
Multimorbidity threshold (used for our study question)	
Outcome Tool	
Number and Types of Long-term Conditions	
Multimorbidity Prevalence	
Effect Estimate Type (OR, RR, IRR)	
Effect Estimates for Multimorbidity (Categories, Effect Size, 95% CI, Adjustment Variables)	
Adjustment Variables	
Use of Patient and Public Involvement and Engagement	
Comments	

ROBINS-E Outcomes

First Author & Year	

Domain 1: Confounding	
Domain 2: Exposure Measurement	
Domain 3: Participant Selection	
Domain 4: Post-Exposure Interventions	
Domain 5: Missing Data	
Domain 6: Outcome Measurement	
Domain 7: Selection of Reported Result	
Overall Risk of Bias	
Comments	

Appendix E: Risk of Bias and Causal Framework

This appendix provides the rationale and structure for how risk of bias was assessed and interpreted using the ROBINS-E framework. It includes:

i. Target Trial Emulation Framework

To support causal interpretation and consistent application of the ROBINS-E tool, a target trial emulation (TTE) framework was used to define the ideal study structure for estimating the effect of childhood socioeconomic disadvantage on adult multimorbidity.

ii. Logic Model and Directed Acyclic Graph (DAG)

To guide risk of bias judgements and identify key confounders and mediators, a logic model and a Directed Acyclic Graph (DAG) were developed based on the literature and causal theory.

iii. Confounder Framework for ROBINS-E

Confounding criteria were defined a priori to guide risk of bias assessments:

i. Target trial emulation for the impact of socioeconomic circumstances in childhood on multimorbidity in adulthood

1. The target trial framework

A target trial is a hypothetical randomised controlled trial (RCT) designed to answer a causal question, which can then be emulated using observational data. Here, the key components of a target trial to explore the causal impact of socioeconomic circumstances (SECs) in childhood on multimorbidity in adulthood are defined.

2. Key components of the target trial

Eligibility criteria

Population:

Individuals born in high-income and low- to middle-income countries, with longitudinal data available from childhood into adulthood.

Inclusion criteria:

Individuals with documented measures of SECs in childhood (e.g., household income, parental education, parental occupation, deprivation indices, housing conditions).

Individuals with medical records or survey data capturing chronic health conditions in adulthood.

Follow-up available into adulthood (>16 years)

Exclusion criteria:5

Individuals with chronic illnesses diagnosed in childhood (to avoid reverse causation).

Studies with insufficient longitudinal follow-up.

Treatment (exposure) strategies

Intervention group:

Individuals exposed to socioeconomic disadvantage in childhood, measured using predefined criteria (e.g., parental income below national poverty thresholds, low parental education, social housing tenure, receipt of welfare benefits).

Control group:

Individuals not exposed to childhood socioeconomic disadvantage (higher parental education, stable financial background, access to high-quality housing and nutrition).

Alternative Strategies:

Stratification by timing of exposure (early childhood, middle childhood, adolescence).

Distinguishing between persistent vs. transient socioeconomic disadvantage.

Assignment (Randomisation)

Since randomisation is not possible in observational studies, statistical adjustment methods are used to control for confounders, emulating a randomised assignment:

Propensity score matching (PSM)

Inverse probability weighting (IPW)

G-computation or marginal structural models (MSMs)

Baseline confounders (e.g., parental health, genetic predispositions) will be adjusted for, and sensitivity analyses will examine potential unmeasured confounders.

Follow-Up (Time Zero)

Time Zero (Baseline): Defined as the age at which childhood SECs are measured (e.g., age 5 or 10).

End of Follow-Up: First diagnosis of multimorbidity (2 or more long term conditions) in adulthood (age 40, 50, or beyond) or last available data point in longitudinal studies.

Outcome of Interest

Primary Outcome:

Multimorbidity in adulthood (defined as the co-occurrence of at least two chronic conditions).

Secondary Outcomes:

Complex multimorbidity (three or more conditions affecting three or more body systems).

Age of onset of multimorbidity.

Mental-physical multimorbidity (coexistence of mental and physical health conditions).

Effect estimates will be expressed as risk ratios (RR), odds ratios (OR), hazard ratios (HR), or incidence rate ratios (IRR).

Causal Contrast & Estimand

Causal Question:

What would be the risk of multimorbidity in adulthood if childhood SECs were different (counterfactual framework)?

Estimand:

Intention-to-treat (ITT) approach: Compares all individuals classified as exposed or unexposed to childhood socioeconomic disadvantage, regardless of later changes in SECs.

Per-protocol analysis: Examines the effect of sustained socioeconomic disadvantage across childhood.

3. Emulation of the Target Trial Using Observational Data

Step 1: Identifying the Cohort

Use longitudinal cohort studies, administrative datasets, or survey-linked medical records to extract eligible individuals.

Ensure harmonisation of childhood SEC measures across studies.

Step 2: Exposure Classification

1. Income-based disadvantage

Threshold: Household income below 60% of the median equivalised disposable income (standard UK/European measure of relative poverty).

Example: In the UK, this equates to an annual household income below approximately £18,000 for a single adult with two children after housing costs.

Alternative measures:

Absolute poverty: Fixed threshold based on minimum necessary resources.

Material deprivation: Inability to afford basic needs (2 out of 6 of, heating, adequate nutrition, shelter, clothing, hygiene, lighting).

Reference:

Department for Work and Pensions (DWP). Households Below Average Income (HBAI), UK Government, 2023.

Eurostat. People at Risk of Poverty or Social Exclusion, European Commission, 2023.

2. Parental occupation

Threshold: Parental occupation classified as working-class or routine/manual occupations according to the National Statistics Socio-Economic Classification (NS-SEC).

Categories:

Higher managerial & professional occupations (Not disadvantaged)

Intermediate occupations (Not disadvantaged)

Lower supervisory & technical occupations (Potentially disadvantaged)

Semi-routine and routine occupations (Disadvantaged group)

Long-term unemployed (Most disadvantaged group)

Alternative classification:

Manual vs. non-manual employment

International Labour Organisation (ILO) classification

Reference:

Office for National Statistics (ONS). NS-SEC Classification Guidelines, UK Government, 2022.

Erikson, R., & Goldthorpe, J. H. (1992). The Constant Flux: A Study of Class Mobility in Industrial Societies. Oxford University Press.

3. Parental employment status

Threshold: At least one parent unemployed or economically inactive during childhood (excluding retirement or disability).

Categories:

Both parents employed (Not disadvantaged)

One parent employed, one unemployed (Moderate disadvantage)

Both parents unemployed (Severe disadvantage)

Single-parent household with unemployment (Severe disadvantage)

Reference:

OECD. *How's Life? Measuring Well-being*, Organisation for Economic Co-operation and Development, 2021.

Bradshaw, J., & Mayhew, E. (2021). *The Well-being of Children in the UK*. Policy Press.

4. Parental education (highest attainment)

Threshold: Highest parental education below upper secondary level (International Standard Classification of Education (ISCED) Level 3).

Categories:

Tertiary education (University degree or higher) – Not disadvantaged

Upper secondary education (A-Levels, High School) – Potentially disadvantaged

Lower secondary education or less (GCSEs, no formal qualifications) – Disadvantaged

Alternative measures:

Years of schooling completed

Parental literacy level

Reference:

UNESCO. International Standard Classification of Education (ISCED) 2011.

Department for Education (DfE). Education Statistics for the UK, UK Government, 2022.

---

5. Housing conditions

Threshold: Overcrowding, poor housing quality, or lack of essential facilities during childhood.

Indicators:

Overcrowding: More than one person per room (excluding bathrooms/kitchens).

Damp/mould issues: Persistent damp/mould as reported in housing surveys.

Heating issues: Inability to afford heating during cold months.

Housing disrepair: Structural issues (leaky roof, broken windows).

Alternative classifications:

Housing deprivation index

Energy poverty (unable to maintain adequate warmth)

Reference:

UK Housing Survey, Ministry of Housing, Communities & Local Government, 2023.

Marmot, M. (2010). Fair Society, Healthy Lives: The Marmot Review.

6. Housing tenure

Threshold: Non-homeownership or reliance on social housing.

Categories:

Owned/mortgaged home (Not disadvantaged)

Private rental (Potential disadvantage)

Social housing (Disadvantaged group)

Temporary housing/homelessness (Severe disadvantage)

Reference:

Social Housing Statistics, UK Government, 2022.

Fitzpatrick, S., et al. (2021). The Homelessness Monitor: England 2021. Crisis UK.

7. Receipt of social welfare benefits

Threshold: Household receiving means-tested government support (e.g., UK Universal Credit, Free School Meals).

Indicators:

Receipt of Free School Meals

Receipt of Housing Benefit or Council Tax Reduction

Receipt of Jobseeker’s Allowance (JSA), Income Support, or Universal Credit

Receipt of child tax credits due to low income

Alternative approaches:

Chronic vs. temporary reliance on benefits

Interaction with other SEC indicators (e.g., low income + welfare receipt = severe disadvantage)

Reference:

DWP. Family Resources Survey, UK Government, 2023.

Hills, J. (2015). Good Times, Bad Times: The Welfare Myth of Them and Us. Policy Press.

Summary of thresholds

**Domain**	**Threshold for Disadvantage**
**Income**	<60% of median equivalised disposable income
**Parental occupation**	Routine/manual occupations (NS-SEC classes 5-8) or long-term unemployed
**Parental employment**	One or both parents unemployed
**Parental education**	Lower secondary education or less (ISCED Level 2)
**Housing conditions**	Overcrowding, damp, poor heating, disrepair
**Housing tenure**	Social housing, temporary housing, or homelessness
**Social welfare benefits**	Receipt of means-tested benefits (e.g., Free School Meals, Housing Benefit)

Step 3: Confounding Control

1. Critical confounders (minimum requirement for avoiding high risk of bias)

These confounders must be controlled for to ensure the study does not have a high risk of bias under ROBINS-E. Failure to adjust for these will likely result in high risk of bias.

A. Time-invariant factors (baseline confounders)

Ethnicity/race – Strongly associated with both SECs and long-term health disparities.

Sex/gender – Influences biological, social, and economic determinants of health.

2. Ideal confounders

These are desirable to adjust for, but their absence does not automatically result in high risk of bias. Their inclusion strengthens causal inference.

A. Additional time-invariant factors

Parental health status – Genetic predisposition, shared environment, and early-life health risks.

Birth weight and gestational factors – Early-life disadvantage markers linked to later health.

B. Broader measures of childhood SECs

If SECs are measured using one indicator, ideally, studies should control for other measures:

Parental education

Parental occupation

Parental income

Housing tenure (social housing vs. private ownership)

Receipt of social welfare benefits

C. Longitudinal childhood SECs

If SECs are measured at one time point, ideally, they should be controlled for at an earlier stage (e.g., SECs at birth if measuring at age 10).

D. Health behaviours (mediators?)

Smoking status in adulthood

Diet and physical activity

Alcohol consumption

Step 4: Outcome Measurement

Identify multimorbidity diagnoses from medical records, national health surveys, or self-reported health assessments.

Standardise multimorbidity definitions across datasets.

Step 5: Statistical Analysis

Compare the risk of multimorbidity between exposed and unexposed groups using:

Inverse Probability of Treatment lighting (IPTW)

G-formula estimation

Targeted maximum likelihood estimation (TMLE)

Conduct sensitivity analyses for unmeasured confounding.

4. Interpretation and Limitations

Strengths of Target Trial Emulation

Minimises common biases (immortal time bias, selection bias).

Provides a structured approach to causal inference.

Ensures alignment with the ROBINS-E framework for assessing risk of bias.

Potential Challenges

Residual confounding: Some childhood factors (e.g., parental health behaviours) may be unmeasured.

Data availability: Longitudinal datasets with both childhood SECs and adult multimorbidity outcomes may be limited.

Generalisability: Differences across countries or health systems may influence results.

5. Conclusion

By emulating a target trial, I structure my observational study to approximate a randomised experiment, reducing bias and strengthening causal inference. This approach will guide the ROBINS-E assessment in the systematic review by identifying critical and ideal confounders.

ii Logic model and directed acyclic graph

Appendix Figure 1: Logic model representing pathways between childhood socioeconomic circumstances and adult multimorbidity

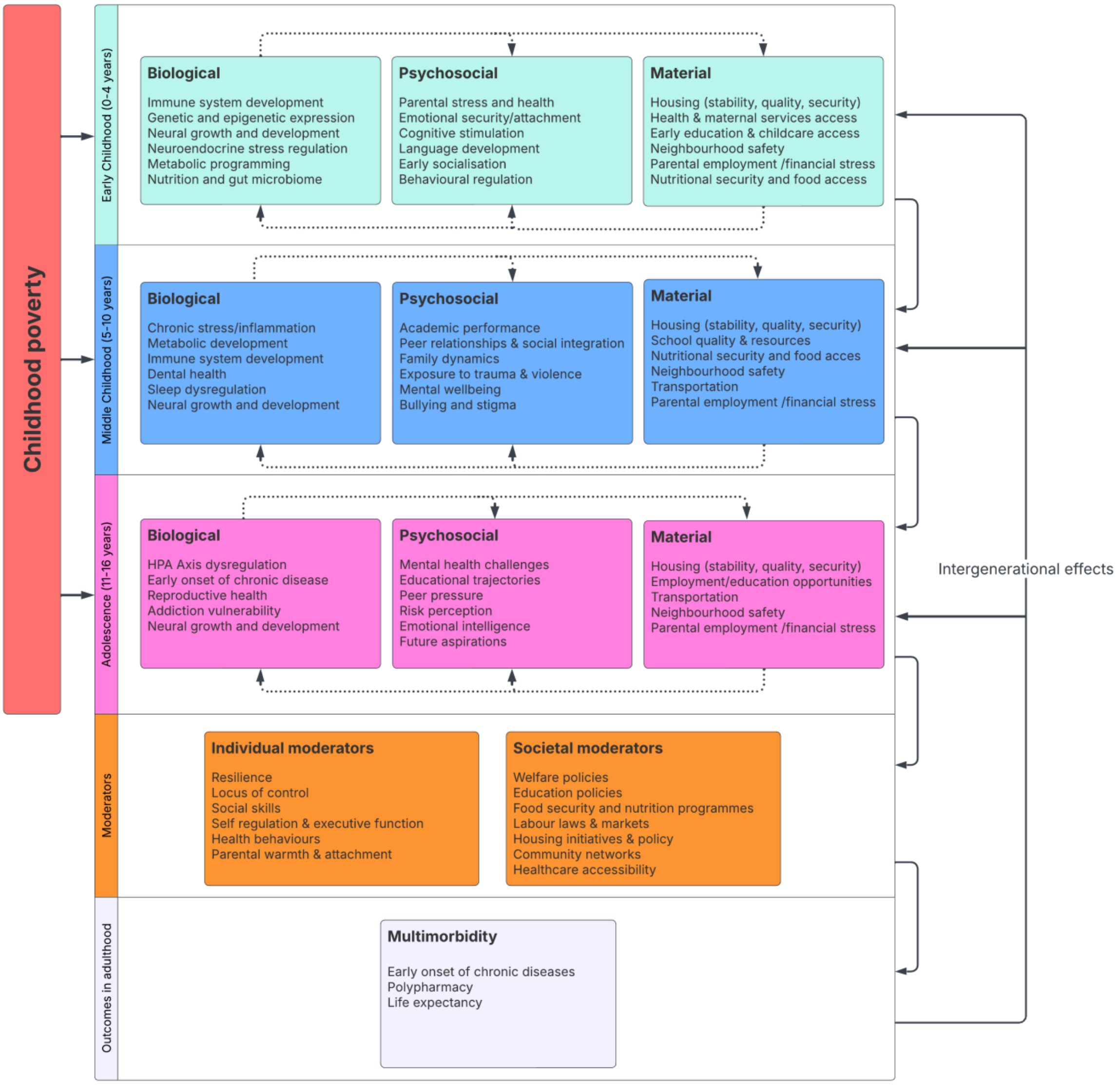

development of multimorbidity in adulthood, structured across three developmental periods: early childhood (0–4 years), middle childhood (5–10 years), and adolescence (11–18 years). The model also recognises the role of moderating influences and highlights the intergenerational transmission of disadvantage.

Early Childhood (0–4 years)

In this formative stage, poverty exerts biological effects via immune system development, neuroendocrine stress responses, and nutritional programming. Psychosocially, early poverty affects parental stress, emotional security, and behavioural regulation, while material deprivation compromises housing, maternal health access, and food security. These foundational disruptions can set early physiological and behavioural trajectories with long-term consequences.

Middle Childhood (5–10 years)

In middle childhood, continued exposure to disadvantage compounds biological risk (e.g., chronic inflammation, sleep dysregulation), while also disrupting psychosocial development through reduced academic performance, social integration, and increased exposure to trauma and stigma. Material disadvantage continues to manifest through school resource inequities, neighbourhood safety, and parental financial stress.

Adolescence (11–18 years)

Adolescence is a sensitive period in which early life disadvantage may consolidate risk. Biologically, stress regulation and early onset of chronic illness become more salient. Psychosocially, risk perception, peer dynamics, and future aspirations are shaped by prior disadvantage. Material constraints—such as limited access to education or employment—compound the effects and can shape long-term life trajectories.

Moderators and Intergenerational Effects

The model also identifies individual (e.g., resilience, executive function) and societal (e.g., welfare policy, community support) moderators that can buffer or exacerbate effects of poverty. These operate across developmental stages and may interact with structural determinants to alter trajectories. Finally, the model acknowledges intergenerational effects, where disadvantage and health outcomes are transmitted across generations, reinforcing cycles of inequality.

Outcomes in Adulthood

The culmination of these interacting pathways is increased risk of multimorbidity in adulthood, including early onset of chronic disease, polypharmacy, and reduced life expectancy. The model provides a conceptual foundation for identifying critical periods, mechanisms, and intervention points across the life course.

iii) Directed acyclic graph

Appendix figure 2: Directed acyclic graph reflecting the causal relationship between childhood socioeconomic circumstances and adult multimorbidity.

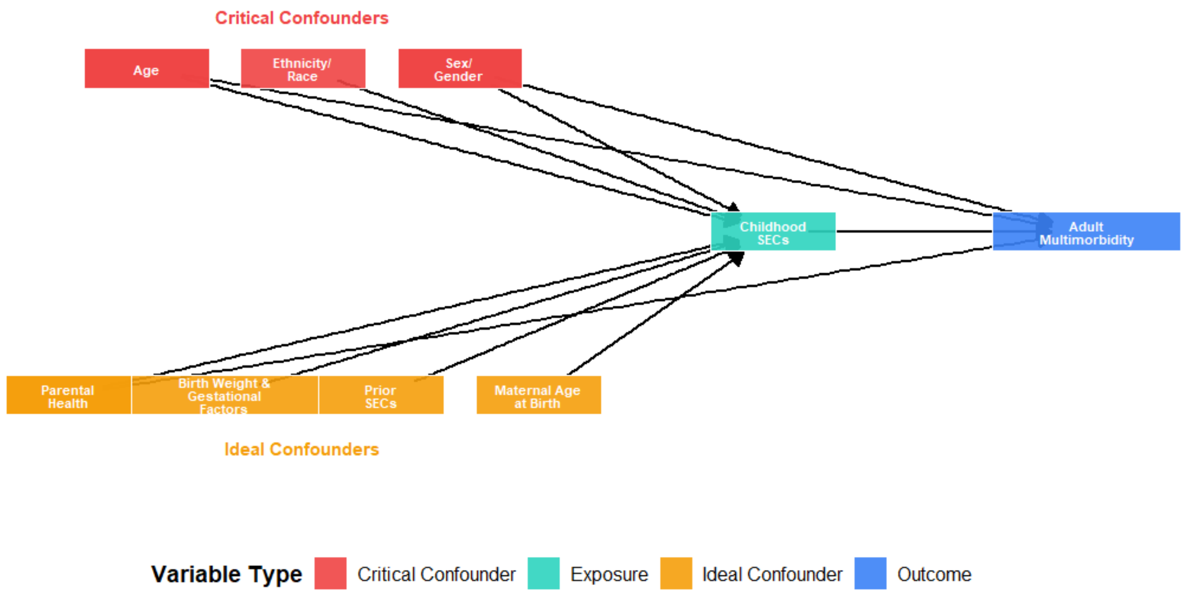

C. Confounder Framework for ROBINS-E

The following were defined a priori to guide risk of bias assessments:

Critical confounders (required to avoid high RoB in confounding domain):

- Gender/Sex
- Race/Ethnicity
- Age (at which outcome is measured)

Ideal confounders (required for low RoB in confounding domain):

- Some measure of prior socioeconomic conditions (e.g. if exposure is age 10, ideally adjusted for SECs at age 0–5)
- Maternal age at birth
- Parental health status

Appendix F: Study-Specific methodological notes and effect estimate selection

Overview

This appendix provides documentation of methodological details for each included study, including exposure and outcome definitions, study design characteristics, and rationale for effect estimate selection and transformation for meta-analysis.

Selection Criteria for Effect Estimates

Ideal Adjustment Set Based on directed acyclic graph (DAG) analysis and theoretical considerations, our ideal adjustment set includes:

Critical confounders (must have, otherwise high risk of bias):

- Gender/sex
- Race/ethnicity
- Age at outcome measurement

Ideal confounders (needed for low risk of bias):

- Prior socioeconomic circumstances before exposure
- Maternal age at birth
- Parental health status

We aimed to avoid models that adjusted for potential mediators on the causal pathway between childhood socioeconomic status and adult multimorbidity, including:

- Adult socioeconomic status (education, income, occupation)
- Adult health behaviours (smoking, physical activity, diet)
- Adult BMI/obesity status

1. Ballesteros et al. (2021)

Study Design: Cross-sectional analysis from the Chilean National Health Survey

Exposure Definition: Childhood perceived economic hardship using a single question with three levels: poor, fair, and good economic conditions.

Outcome Definition: Multimorbidity defined via self-report of 2+ physician-diagnosed chronic conditions.

Available Effect Estimates:

- Crude model: Poor vs. Good: OR = 1.34 (95% CI: 1.23-1.47); Fair vs. Good: OR = 1.16 (95% CI: 1.08-1.24)
- Adjusted model: None available

Selected Estimate: OR = 1.34 (95% CI: 1.23-1.47) for poor vs. good childhood economic conditions

Selection Rationale: Only unadjusted estimates were available. While suboptimal due to potential confounding, this was the only available estimate for synthesis.

2. Dekhtyar et al. (2019)

Study Design: Prospective cohort using Swedish national registers

Exposure Definition: Father's occupation at age 10 classified into five socioeconomic levels, collapsed to manual vs. nonmanual for analysis.

Outcome Definition: Multimorbidity defined using register-based diagnoses of 2+ conditions from health records.

Covariates: Original study adjusted for sex, birth year, region, and adult SES

Available Effect Estimates:

- Unadjusted model: $\beta \times$ time = -0.034 (95% CI: -0.071, 0.003)
- Fully adjusted model: $\beta \times$ time = 0.012 (95% CI: -0.027, 0.051)

Selected Estimate: OR = 1.10 (95% CI: 0.94-1.30) for manual vs. nonmanual parental occupation

Selection Rationale: Original study reported disease accumulation rates, not multimorbidity odds ratios. We reconstructed a 2×2 contingency table from raw counts in Table 1 to calculate OR for having 2+ conditions.

Transformation Applied: Calculated from reconstructed contingency table:

- Manual occupation: 612 with 2+ conditions, 405 with 0-1 conditions
- Nonmanual occupation: 909 with 2+ conditions, 663 with 0-1 conditions
- OR = (612 × 663) / (405 × 909) = 1.10

3. Haas (2008)

Study Design: Longitudinal analysis from the US Health and Retirement Study (HRS), including ~6,000 participants born between 1931 and 1941, followed through to 2002

Exposure Definition: Father's educational attainment, categorised as: less than high school, high school graduate, some college, or college and above (reference). Data collected retrospectively.

Outcome Definition: Multimorbidity defined as the presence of two or more chronic conditions, including heart disease, diabetes, cancer, stroke, and others, based on self-report.

Available Effect Estimates:

- Model 2 (+ childhood factors): OR = 0.93 (95% CI: 0.91-0.96)
- Model 3 (+ adult SES): OR = 0.98 (95% CI: 0.94-1.00)
- Model 4 (+ health behaviours): OR = 0.98 (95% CI: 0.95-1.01)
- Model 5 (+ random slopes): OR = 0.98 (95% CI: 0.95-1.01)

Selected Estimate: OR = 1.08 (95% CI: 1.04-1.10) for one-unit increase in childhood disadvantage

Selection Rationale: Selected Model 2 as it adjusts for age, sex, and birth cohort without over-adjustment for adult SES (potential mediator). Model adjusted for age, sex, and race. Estimate used excludes adjustment for adult SES (education and income) to avoid controlling for mediators.

Transformation Applied: Original OR was for increasing advantage (protective effect). We inverted to reflect change per unit increase in disadvantage: OR = 1/0.93 = 1.08 (1.04, 1.10)

Risk of Bias: Moderate - adjusted for key confounders but missing some ideal confounders

4. Henchoz et al. (2019)

Study Design: Cross-sectional analysis from the CoLaus study in Lausanne, Switzerland; only individuals aged 40+ included

Exposure Definition: Used a 10-point Likert scale to retrospectively assess perceived childhood financial situation at age 15. Participants were categorised into tertiles: low (1–3), middle (4–7), and high (8–10).

Outcome Definition: Multimorbidity defined as 2+ chronic conditions reported via health questionnaire.

Available Effect Estimates:

- Model 1 (sex + cohort): OR = 1.23 (95% CI: 1.02-1.48)
- Model 2 (mutually adjusted): OR = 0.98 (95% CI: 0.79-1.22)
- Model 3 (fully adjusted): OR = 0.94 (95% CI: 0.74-1.19)

Selected Estimate: OR = 1.23 (95% CI: 1.02-1.48) for poor vs. Not poor childhood economic environment

Selection Rationale: Selected Model 1 as it adjusts for sex and birth cohort (cohort effectively controls for age in this 65-70 year old sample across three birth cohorts 1934-1948). Model 2 mutually adjusts for all childhood adversity factors simultaneously, which over-adjusts by

conditioning on potential mediators and co-exposures. Model 3 additionally over-adjusts by including adult SES and health behaviours.

Risk of Bias: Moderate - adjusted for key demographic factors but missing some confounders

5. Keetile et al. (2023)

Study Design: Cross-sectional analysis from Botswana STEPwise survey

Exposure Definition: Parental socioeconomic status coded from retrospective questions on family wealth and education.

Outcome Definition: 2+ non-communicable diseases (NCDs) identified by self-report and clinician diagnosis.

Available Effect Estimates:

- Adjusted model: Low vs. High childhood SES: OR = 1.78 (95% CI: 1.11-2.68); Middle vs. High: OR = 1.32 (95% CI: 0.29-1.79)

Selected Estimate: OR = 1.78 (95% CI: 1.11-2.68) for low vs. high childhood SES

Selection Rationale: Single fully adjusted model available. Adjustment set included age, gender, education, residence, marital status, employment, and current wealth - some potential over-adjustment for adult SES factors.

6. Pati et al. (2023)

Study design: Cross-sectional analysis using two nationally representative datasets: Longitudinal Ageing Study in India (LASI) wave 1 (2017-2018) and Brazilian Longitudinal Study of Aging (ELSI-Brazil) (2015-2016), analysed separately.

Study population:

- India (LASI): n=51,481 adults aged ≥50 years from 29 states and 6 union territories
- Brazil (ELSI-Brazil): n=8,730 adults aged ≥50 years from 70 municipalities across 5 geographic regions

Exposure definition: Self-reported childhood economic status assessed retrospectively for period from birth to age 16, categorised as: pretty well off, average, poor, or varied a lot. Participants were asked about their family's economic status during childhood using the question format provided in the original surveys.

Outcome definition: Multimorbidity defined as presence of 2+ chronic conditions from a standardised list of 11 self-reported physician-diagnosed conditions uniform across both datasets: hypertension, diabetes, stroke, cancer, chronic lung diseases, chronic heart diseases, bone/joint

diseases, neurological or psychiatric problems, high cholesterol, chronic renal failure, and chronic oral conditions.

Outcome prevalence:

- India: 25.53% (95% CI not specified in paper)
- Brazil: 55.24% (95% CI not specified in paper)

Statistical analysis: Generalised linear model with log link to estimate adjusted prevalence ratios (APR). Models adjusted for age (50-59, 60-69, ≥70 years) and gender (male/female). All analyses incorporated survey weights to account for complex sampling design.

Available effect estimates (original - well off vs. poor):

- India: Pretty well off vs. poor: APR = 1.32 (95% CI: 1.14-1.53)
- Brazil: Pretty well off vs. poor: APR = 0.93 (95% CI: 0.84-1.03)

Effect estimate inversion for systematic review:

- India: Poor vs. pretty well off: APR = 0.76 (95% CI: 0.65-0.88) [calculated as 1/1.32 with CI bounds inverted]
- Brazil: Poor vs. pretty well off: APR = 1.08 (95% CI: 0.97-1.19) [calculated as 1/0.93 with CI bounds inverted]

Selected estimate: Inverted effect estimates (poor childhood economic status vs. pretty well off) used separately for each country in meta-analysis.

Selection rationale:

1. Models adjusted only for age and gender, providing minimal but essential demographic adjustment whilst avoiding over-adjustment for potential mediators
2. Effect estimates were mathematically inverted to align with systematic review's focus on childhood socioeconomic disadvantage as the exposure of interest
3. Country-specific effects analysed separately due to significant heterogeneity between India and Brazil (opposite directions of association)
4. Cross-sectional design limits causal inference but provides large, nationally representative samples from two major LMICs

Study limitations noted: Self-reported childhood economic status subject to recall bias; cross-sectional design prevents causal inference; chronic conditions based on self-report of physician diagnosis may be subject to healthcare access bias.

7. Pavela (2016)

Study Design: Cross-sectional analysis using HRS (Health and Retirement Study) data

Exposure Definition: Multiple socioeconomic characteristics including mother's education, father's education, father ever unemployed.

Outcome Definition: Self-reported multimorbidity using a check-list of conditions.

Available Effect Estimates:

- Model 3 (childhood factors): Separate ORs for 1, 2, 3, 4, 5+ conditions
- Model 4 (+ demographics): Separate ORs for 1, 2, 3, 4, 5+ conditions
- Model 5 (+ adult SES): Separate ORs for 1, 2, 3, 4, 5+ conditions
- Model 6 (fully adjusted): Separate ORs for 1, 2, 3, 4, 5+ conditions

Selected Estimate: OR = 1.05 (95% CI: 0.95-1.16) for low vs. high childhood SES [pooled from Model 4]

Selection Rationale: Selected Model 4 estimates as they adjusted for essential demographics without over-adjusting for adult SES. We meta-analyzed ORs for 2, 3, 4, and 5+ conditions to derive a single multimorbidity estimate.

Transformation Applied: Conducted random-effects meta-analysis pooling ORs across multimorbidity categories (2+, 3+, 4+, 5+ conditions) using multilevel modeling to account for shared reference group.

8. Putnam et al. (2013)

Study Design: Cross-sectional analysis using NCS-R dataset

Exposure Definition: Childhood poverty defined as a binary composite exposure. Participants were categorised as exposed to economic adversity if they endorsed any of the following items: CH19 (receipt of government assistance in childhood), CH30_1C (going without necessities due to caregiver spending), or CH30_1D (going hungry or not receiving regular meals). This definition was confirmed by direct correspondence with the original authors.

Outcome Definition: Multimorbidity operationalised as the presence of disorders in two or more of the following four diagnostic domains: mood, anxiety, impulse-control, and substance use disorders, as defined by DSM-IV criteria.

Available Effect Estimates:

- Individual models: Separate ORs for 2, 3, 4 disorder categories
- Joint models: Combined adversity effects

Selected Estimate: OR = 2.10 (95% CI: 1.80-2.45) for childhood economic adversity

Selection Rationale: We reconstructed the analysis using NCS-R data to derive a single multimorbidity estimate, following the authors' methodology for defining economic adversity.

Transformation Applied: We accessed the ICPSR 20240 version of the dataset and reproduced frequency tables to confirm similarity with author-reported distributions. Created 2×2 contingency table from raw data:

- Economic adversity present: 290 with multimorbidity, 628 without
- Economic adversity absent: 1,000 with multimorbidity, 4,547 without
- OR = 2.10, 95% CI = [1.80, 2.45] confirmed through logistic regression

9. Schramm et al.

Study Design: Cross-sectional analysis using GEDA 2012 data with post-stratification weights applied

Exposure Definition: Constructed a composite variable using highest education level of either parent, based on ISCED classification. Where only one parent's education was available, that parent's value was used.

Outcome Definition: Defined as two or more chronic conditions among 13 conditions asked via self-report in GEDA 2012.

Available Effect Estimates:

- Unadjusted: Medium vs. Long parental education: OR = 1.26 (95% CI: 1.19-1.34); Short vs. Long: OR = 1.68 (95% CI: 1.59-1.78)
- Adjusted for individual education: Medium vs. Long: OR = 1.36 (95% CI: 1.07-1.73); Short vs. Long: OR = 1.48 (95% CI: 1.15-1.89) [for "Many conditions" class only]

Selected Estimate: OR = 1.68 (95% CI: 1.59-1.78) for short vs. long parental education

Selection Rationale: Selected unadjusted estimates to avoid over-adjustment for individual education (potential mediator). Authors provided supplementary data allowing calculation of conventional multimorbidity ORs.

Transformation Applied: Calculated from supplementary data:

- Long education: 1,807 with 2+ conditions, 14,431 with <2 conditions
- Short education: 8,293 with 2+ conditions, 39,430 with <2 conditions

10. Zhao et al.

Study Design: Cross-sectional analysis using CHARLS data with varying exposure definitions across papers

Exposure Definition: Multiple papers use mother's/father's education and combined parental education, with family financial situation as primary exposure.

Outcome Definition: 2+ chronic diseases based on CHARLS data.

Available Effect Estimates:

- Model 1 (early-life factors): Worse off vs. Better off family: OR = 1.13 (95% CI: 0.95-1.35)
- Model 2 (fully adjusted): Worse off vs. Better off family: OR = 1.32 (95% CI: 1.08-1.61)

Selected Estimate: OR = 1.13 (95% CI: 0.95-1.35) for worse off vs. better off family financial situation

Selection Rationale: Selected Model 1 to avoid over-adjustment for adult factors in Model 2. Model 1 focused on early-life factors without including adult SES mediators.

Transformation Applied: Inverted original ORs as study used most disadvantaged as reference:

- Original: Better off vs. Worse off: OR = 0.88 (95% CI: 0.74-1.05)
- Inverted: Worse off vs. Better off: OR = 1/0.88 = 1.13

Risk of Bias: Moderate - adjusted for multiple early-life factors

Summary of Selection Principles

1. Prioritised minimal sufficient adjustment: Selected models with essential demographic adjustment (age, sex, race/ethnicity) without over-adjustment for potential mediators
2. Over-adjustment: Aimed to avoid selecting models adjusting for adult SES, health behaviours, or BMI that may lie on the causal pathway
3. Standardised comparisons: Consistently used socioeconomically advantaged groups as reference categories through inversion where necessary
4. Transformations: Documented all calculations and data manipulations used to derive final effect estimates

This systematic approach ensures consistency across studies while minimizing bias from both under- and over-adjustment in the meta-analysis.

Appendix G - Clarifications and amendments to systematic review protocol

During the course of screening, several refinements were made to ensure consistent application of the inclusion criteria and appropriate interpretation of exposures and outcomes. These clarifications did not alter the core eligibility framework set out in the registered PROSPERO protocol but served to operationalise key decisions transparently:

1. **Exclusion of education as a sole childhood SEC measure**
   Educational attainment (the participant's own) was excluded as a standalone measure of childhood socioeconomic circumstances (SEC). This decision is grounded in concerns about temporal validity (as education spans into adulthood), conceptual clarity(education is an outcome as well as a potential mediator), and overlap with existing literature

2. **Requirement for isolated childhood SEC effect estimates**
   Studies were excluded where childhood SEC was included only as part of a composite exposure or covariate and could not be disaggregated to assess its independent association with the outcome. This ensured only those estimates directly attributable to childhood SEC were included.

3. **Exclusion of count-based multimorbidity measures without MM threshold**
   Where the outcome was a disease count (e.g., 0, 1, 2, … conditions), studies were excluded if it was not possible to determine whether the regression estimates reflected a threshold of 2+ conditions (i.e., multimorbidity). Inclusion of these would risk bias, as the outcome may primarily reflect differences below the MM threshold, misrepresenting associations of interest.

4. **Interpretation of Risk of Bias Tool (ROBINS-E)**
   In line with the ROBINS-E guidance, the embedded pre-screening tool was used to determine whether a full risk-of-bias assessment was warranted for each result. Where no attempt was made to control for confounding or where exposure/outcome measurement was clearly inappropriate, results were classified as having 'very high risk of bias' and not assessed further. In such cases, only a single row per study was retained, with a footnote indicating this decision. This does not represent a deviation from the protocol but rather reflects standard practice in applying ROBINS-E; it was simply not made explicit in the original protocol.

Appendix H: Estimating the Relative Index of Inequality (RII)

Overview

To synthesise findings across studies using grouped childhood socioeconomic condition (SEC) data, we derived the Relative Index of Inequality (RII) for each exposure. The RII expresses the relative risk of multimorbidity across the socioeconomic hierarchy, comparing the most disadvantaged group to the most advantaged while accounting for population distribution across all intermediate groups.

RII Calculation Methods

We employed three statistical methods with a hierarchy of preference to ensure robust estimates in our primary analysis while maximising data use in sensitivity analysis.

Method 1: Binomial Generalised Linear Model (GLM)

Preferred method for analysis

Where group-level case counts and population sizes were available, we fitted logistic regression models to estimate RII from ridit scores:

```
glm(cbind(MM_cases, Group_N - MM_cases) ~ Ridit_Score, family = binomial())
```

Requirements:

- Complete case count and population size data for all groups
- Minimum of 3 socioeconomic groups for primary analysis (to establish meaningful gradient)

Interpretation: The exponentiated coefficient for Ridit_Score represents the RII.

Method 2: Weighted Linear Regression on log(OR)

Alternative method for primary analysis

Where multiple odds ratios (ORs) and standard errors were reported, we regressed log(OR) on ridit scores using inverse-variance weights:

```
lm(log(OR) ~ Ridit_Score, weights = 1/SE²)
```

Standard approach (≥3 data points):

- Conventional weighted least squares regression
- Standard error estimation from model residuals

Requirements:

- At least 2 non-reference groups with reported ORs and standard errors

- Inverse-variance weighting to account for precision differences

Method 3: Single OR Extrapolation

Used in sensitivity analysis only

When only one OR was available relative to a reference group, we approximated RII using:

RII = exp(log(OR) / |ridit_difference|)

Limitations:

- Assumes linear gradient across socioeconomic hierarchy
- Based on strongest assumption of linearity
- Excluded from primary analysis
-

Analysis strategy

Primary analysis

Objective: Generate RII estimates using only most reliable methods

Inclusion criteria:

- Studies with ≥3 socioeconomic groups AND complete case/population data (Method 1), OR
- Studies with ≥2 non-reference ORs with standard errors (Method 2)
- Exclusion of single OR extrapolation

Sensitivity analysis

Objective: Maximise data use by including all calculable RII estimates

Inclusion criteria:

- All studies meeting primary analysis criteria
- Additional studies using single OR extrapolation when robust methods unavailable
- Studies with only 2 socioeconomic groups (using appropriate methods)

Method hierarchy:

1. Binomial GLM (any number of groups with complete data)

2. WLS on log(OR)
3. Single OR extrapolation (as fallback method)

Rationale: This approach tests whether conclusions change when including studies with weaker methodological foundations.

Implementation

Data processing

1. Data validation: Conversion to numeric format
2. Group enumeration: Automatic counting of socioeconomic categories per study
3. Method selection: Hierarchical algorithm selecting optimal approach based on data availability
4. Quality checks: Validation of model convergence and parameter estimation
5. Results compilation

Software implementation

All analyses conducted in R using:

- Base GLM functions for binomial regression
- Weighted least squares via lm() with inverse-variance weights

Strengths

1. Hierarchical approach ensures optimal method selection based on data quality
2. Transparency in method selection
3. Reproducibility through standardised procedures

Limitations

1. Between-study heterogeneity in socioeconomic group definitions and reference categories
2. Risk of bias uniformly high across included studies
3. Population differences may affect generalisability of pooled estimates

4. Linear assumption in single OR extrapolation may not reflect true dose-response Quality Assurance

Appendix I: RII estimates and analysis

Primary analysis results

The primary analysis included 8 effect estimates from 5 studies with either ≥3 socioeconomic groups for binomial GLM or ≥2 non-reference odds ratios for weighted linear regression.

Primary analysis RII estimates

Study	Method	RII	95% CI
Ballasteros (Perceived econ circ)	Binomial GLM	1.48	1.32–1.66
Pati (Brasil)	Binomial GLM	1.54	1.26–1.87
Pati (India)	Binomial GLM	0.46	0.42–0.49
Pavela (Father's education)	WLS on log(OR)	1.36	0.74–2.53
Schramm	Binomial GLM	2.06	1.93–2.20
Zhao (Father's education)	Binomial GLM	0.86	0.71–1.03
Zhao (Mother's education)	Binomial GLM	1.02	0.70–1.48
Zhao (Perceived econ circ)	Binomial GLM	1.49	1.25–1.78

Appendix figure 3: Forest plot of relative index of inequality - Primary analysis. Association between childhood socioeconomic circumstances and adult multimorbidity. (≥3 groups for binomial GLM, ≥2 ORs for weighted regression)

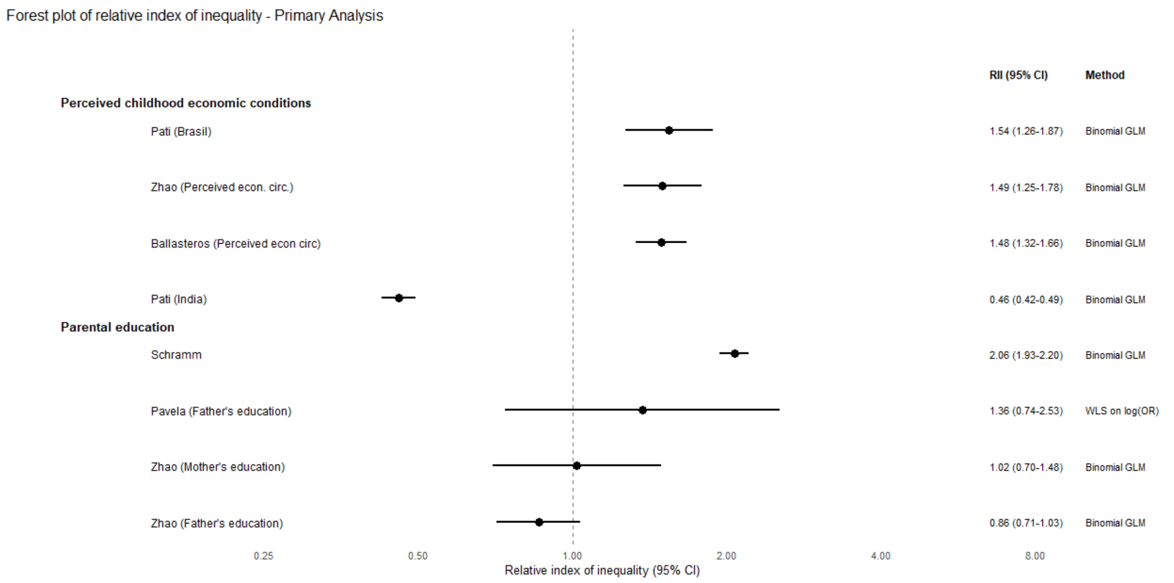

Primary analysis interpretation

The primary analysis demonstrates evidence for socioeconomic gradients in adult multimorbidity associated with childhood circumstances across diverse populations and exposure types:

Magnitude of associations:

- Moderate positive effects (RII 1.4-1.6): Ballasteros perceived economic circumstances (RII 1.48), Pati Brasil (RII 1.54), and Zhao perceived economic circumstances (RII 1.49) show consistent moderate gradients
- Strong positive effects (RII >2.0): Schramm parental education demonstrates substantial inequalities (RII 2.06, 95% CI 1.93–2.20)
- Modest/null effects (RII 0.8-1.4): Pavela father's education (RII 1.36, wide CI), Zhao mother's education (RII 1.02), and Zhao father's education (RII 0.86) show weaker or non-significant associations
- Protective effect: Pati India shows strong inverse association (RII 0.46, 95% CI 0.42–0.49)

Exposure-specific patterns:

- Perceived childhood economic conditions: Consistent positive gradients across studies (Ballasteros: 1.48; Zhao: 1.49)
- Parental education: Mixed patterns with strong effect in one population (Schramm: 2.06) but weaker/null effects in others (Zhao studies, Pavela)
- Geographic variation: Notable between Brazilian (1.54) and Indian (0.46) Pati study populations

Precision and uncertainty:

- Six of eight effect estimates demonstrate relatively precise estimates with narrow confidence intervals excluding the null
- Zhao father's and mother's education show wider confidence intervals encompassing the null value, indicating greater uncertainty
- Consistent direction (except Pati India) across most estimates supports genuine gradient patterns despite varying precision

2. Sensitivity analysis results

The sensitivity analysis expanded coverage to 14 effect estimates from 8 studies by including effect estimates using single OR extrapolation and those with only two socioeconomic groups

Sensitivity analysis RII estimates

Study	Method	RII	95% CI
Primary analysis studies:			
Ballasteros (Perceived econ circ)	Binomial GLM	1.48	1.32–1.66
Pati (Brasil)	Binomial GLM	1.54	1.26–1.87
Pati (India)	Binomial GLM	0.46	0.42–0.49
Pavela (Father's education)	WLS on log(OR)	1.36	0.74–2.53
Schramm	Binomial GLM	2.06	1.93–2.20
Zhao (Father's education)	Binomial GLM	0.86	0.71–1.03
Zhao (Mother's education)	Binomial GLM	1.02	0.70–1.48
Zhao (Perceived econ circ)	Binomial GLM	1.49	1.25–1.78
**Additional studies in sensitivity analysis:**			
Dekhtyar	Binomial GLM	1.22	0.88–1.68
Henchoz	Binomial GLM	1.49	1.02–2.16
Pavela (Father's employment status)	Single OR extrapolation	1.23	0.97–1.57
Pavela (Mother's education)	Single OR extrapolation	1.48	0.96–2.27
Pavela (Perceived econ circ)	Single OR extrapolation	1.10	0.90–1.35
Putnam	Binomial GLM	4.41	3.23–6.00

Appendix Figure 4: Forest plot of relative index of inequality - Sensitivity analysis. Association between childhood socioeconomic circumstances and adult multimorbidity. All available methods (includes 2-group studies and single OR extrapolation)

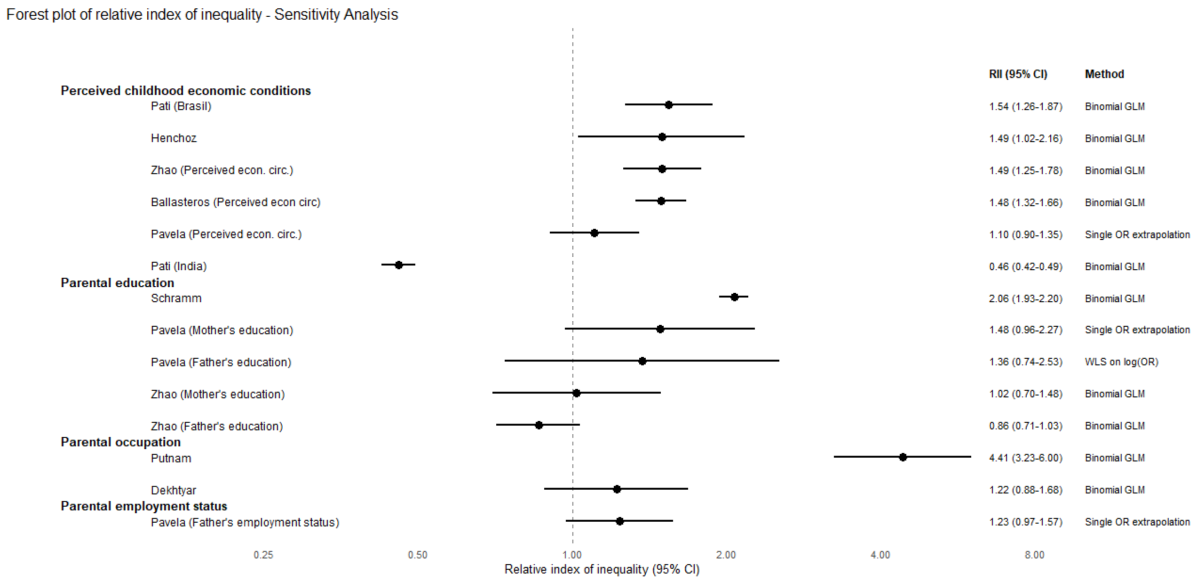

Sensitivity analysis interpretation

Consistency with primary findings:

- Additional studies show predominantly positive associations with childhood disadvantage
- No major contradictions emerge when including methodologically weaker studies

Additional insights:

- Putnam study shows exceptionally strong gradient (RII 4.41, 95% CI 3.23–6.00), representing the most extreme effect observed and suggesting marked inequalities in some populations
- Pavela studies demonstrate consistent modest positive associations across multiple SEC indicators (RII range 1.10-1.48), supporting findings

Methodological validation:

- Two-group studies using binomial GLM yield plausible estimates consistent with multi-group studies
- Single OR extrapolation produces reasonable estimates that align with more robust methods
- Method choice does not systematically bias results in unexpected directions
- Linear assumptions appear reasonable based on concordance between methods

Effect size distribution:

- Range: 0.46 (Pati India - protective) to 4.41 (Putnam - very strong risk)
- Typical positive range: 1.10-1.54 for most risk associations
- Precision of estimates: 10 of 14 effect estimates demonstrate confidence intervals that exclude the null value, indicating relatively precise effect estimation

- Consistency: 13 of 14 studies show positive direction (only Pati India reversed)

Overall synthesis

Strength of evidence

Primary analysis provides evidence for socioeconomic gradients in adult multimorbidity associated with childhood SEC, based on:

- Eight effect estimates from five independent populations across diverse geographic settings
- Multiple socioeconomic exposures (perceived conditions, parental education, occupation)
- Consistent directions of association with few exceptions

Sensitivity analysis reinforces these conclusions while demonstrating:

- Robustness to methodological choices and inclusion criteria
- Consistency across different analytical approaches
- Range of effect sizes from modest to substantial

Clinical and Public Health Implications

Persistent health effects:

- Childhood socioeconomic circumstances have lasting impacts on adult multimorbidity risk
- Graded relationships suggest dose-response patterns
- Multiple pathways (economic, educational, occupational) contribute to this effect

Population impact:

- Effect sizes (typically RII 1.4-2.1) represent clinically meaningful increases in multimorbidity risk
- Extreme effects (Putnam RII 4.41) suggest marked inequalities in some populations
- Protective patterns (Pati India) may indicate context dependent effects of exposure to socioeconomic adversity in childhood

Limitations and Considerations

Study quality limitations:

- Universal high risk of bias across included studies limits causal inference
- Cross-sectional designs in most studies prevent assessment of temporality
- Recall bias potential for retrospective SEC assessment

Methodological considerations:

- Between-study heterogeneity in SEC measurement and reference categories
- Single OR extrapolation relies on strong linearity assumptions
- Two-point slope method assumes independence of effect estimates
- Population specificity may limit generalisability of pooled estimates

Interpretation caveats:

- Pati India reversal requires careful interpretation and may reflect unique cultural factors
- Wide confidence intervals for some estimates (particularly Pavela studies) indicate uncertainty
- Extreme effects (Putnam) may reflect population-specific factors

Appendix J: Leave-one-out sensitivity analysis

Methods

Leave-one-out (LOO) sensitivity analysis was performed to assess the robustness of pooled estimates and identify studies with disproportionate influence on the meta-analysis results. For each analysis, we systematically excluded one study at a time and recalculated the pooled odds ratio using random-effects meta-analysis (REML method). This process was repeated for all included studies, with results compared to the full model estimate.

Results: Perceived Childhood Economic Circumstances

LOO analysis was conducted on 7 studies examining perceived childhood economic circumstances and multimorbidity risk. Results are presented in Table F1.

Leave-One-Out Sensitivity Analysis Results

Excluded Study	OR	95% CI	I^2
Pati (India)	1.18	1.05-1.32	77.2%
Pati (Brasil)	1.08	0.83-1.40	95.1%
Pavela (USA)	1.08	0.83-1.40	95.3%
None (Full model)	1.07	0.87-1.33	94.7%
Henchoz (Switzerland)	1.05	0.82-1.35	96.0%
Zhao (China)	1.04	0.81-1.32	95.7%
Ballesteros (Colombia)	1.03	0.81-1.30	93.9%

Summary: Range of ORs: 1.03-1.18; Range of I^2: 77.2%-96.0%

The analysis revealed that excluding the Pati (India) study resulted in the largest change in pooled estimates, with the OR increasing from 1.07 to 1.18 and heterogeneity decreasing substantially from 94.7% to 77.2%.

Leave-One-Out Analysis

Leave-one-out sensitivity analysis revealed important insights about study influence on pooled estimates for perceived childhood economic circumstances. When systematically excluding individual studies, pooled odds ratios ranged from 1.03 to 1.18, indicating moderate sensitivity to individual study inclusion.

The most notable finding was the substantial impact of excluding the Pati (India) study, which resulted in:

- Increased effect size: OR increased from 1.07 (95% CI: 0.87-1.33) to 1.18 (95% CI: 1.05-1.32)
- Improved precision: The confidence interval shifted from non-significant to significant
- Reduced heterogeneity: I^2 decreased markedly from 94.7% to 77.2%

This pattern suggests the Pati (India) study may be acting as an outlier that is diluting the overall effect estimate and inflating heterogeneity. Several factors may explain this:

1. Population differences: The Indian population may have different baseline health risks or socioeconomic gradients compared to high-income countries
2. Measurement differences: Cultural variations in how economic circumstances are perceived or reported
3. Healthcare system differences: Differential access to diagnosis and treatment affecting multimorbidity ascertainment

Clinical and Public Health Implications

The sensitivity analysis suggests that the association between poor childhood economic circumstances and multimorbidity may be stronger and more consistent than the full model indicates. Excluding the potentially outlying Indian study reveals:

- A statistically significant 18% increased odds of multimorbidity (OR 1.18, 95% CI: 1.05-1.32)
- Much lower heterogeneity (I^2 = 77.2%), suggesting more consistent effects across the remaining studies
- Greater confidence in the pooled estimate's reliability

This finding supports the biological plausibility of early-life socioeconomic disadvantage programming long-term health risks through established pathways including chronic inflammation, stress response dysregulation, and accelerated cellular aging.

Limitations

While this sensitivity analysis provides valuable insights, we acknowledge that excluding studies post-hoc based on influence should be interpreted cautiously. The decision to exclude studies should ideally be based on a priori methodological concerns rather than statistical influence alone.

Appendix K: Subgroup analysis

Overview

This appendix presents detailed results from pre-specified subgroup analyses conducted to explore potential sources of heterogeneity in the association between childhood socioeconomic disadvantage and adult multimorbidity. Subgroup analyses were performed by geographic region, study design, multimorbidity definition, and exposure type to investigate whether observed heterogeneity could be explained by these study characteristics.

Methods

Subgroup analyses were conducted using random-effects meta-analysis within each subgroup, with between-subgroup heterogeneity assessed using Q-tests. Heterogeneity within subgroups was quantified using I^2 statistics. All analyses used the same random-effects model specifications as the main meta-analyses (REML estimator). Subgroup analyses were only performed when at least two studies contributed to each subgroup.

Results by Subgroup

Geographic Region Analysis

Appendix Figure 5: *Forest plot of odds ratios for the association between perceived childhood socioeconomic adversity and adult multimorbidity, showing individual study estimates and pooled random-effects meta-analysis. Black diamond represents the pooled odds ratio and 95% confidence interval. Squares represent study-specific odds ratios, with the size of each square proportional to the study's weight in the random-effects meta-analysis. Horizontal lines represent 95% confidence intervals for individual studies. Separated by geographical region*

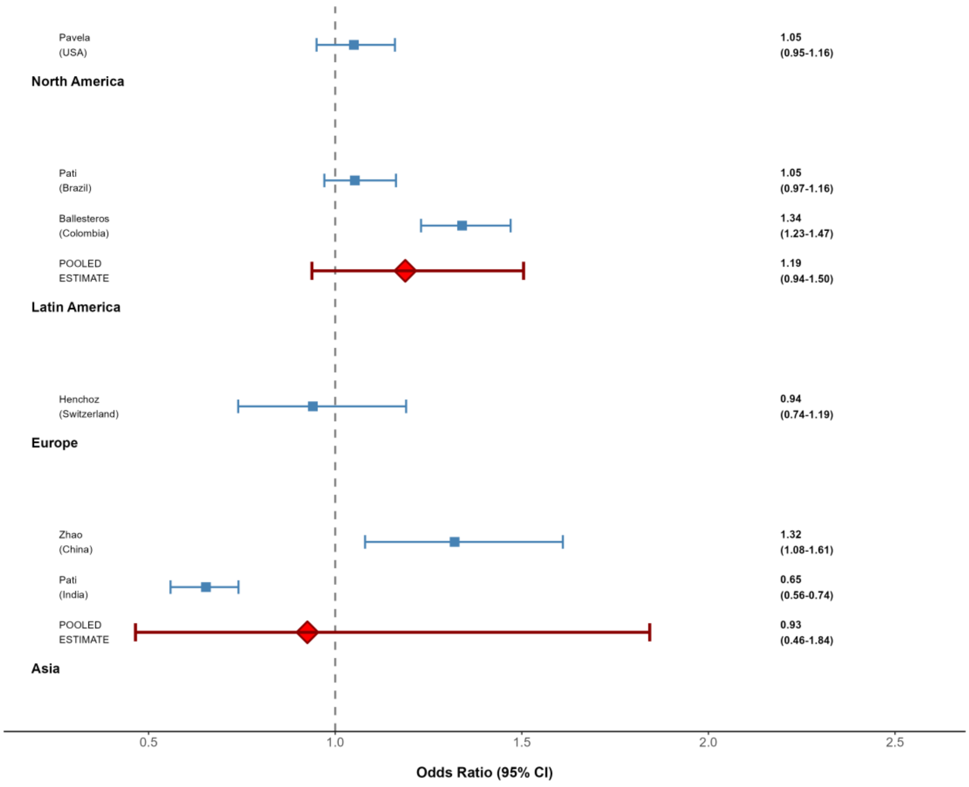

Perceived Childhood Economic Adversity by Geographic Region

Asia (n=2 studies)

- Pooled OR: 0.93 (95% CI: 0.46-1.84)
- I^2: 96.9% (very high heterogeneity)
- Studies: Pati et al. (India): OR 0.65 (0.56-0.74); Zhao et al. (China): OR 1.32 (1.08-1.61)
- Interpretation: Substantial heterogeneity between Asian studies, with opposing effect directions. The Indian study suggests a protective association while the Chinese study indicates increased risk.

Latin America (n=2 studies)

- Pooled OR: 1.19 (95% CI: 0.94-1.50)
- I^2: 92.8% (very high heterogeneity)
- Studies: Ballesteros et al. (Colombia): OR 1.34 (1.23-1.47); Pati et al. (Brazil): OR 1.05 (0.97-1.16)
- Interpretation: Both studies suggest harmful associations, but with considerable variation in effect size. Colombian study shows stronger association than Brazilian study.

Europe (n=1 study)

- OR: 0.94 (95% CI: 0.74-1.19)
- Study: Henchoz et al. (Switzerland)
- Interpretation: Single study suggests null association with wide confidence interval.

North America (n=1 study)

- OR: 1.05 (95% CI: 0.95-1.16)
- Study: Pavela et al. (USA)
- Interpretation: Single longitudinal study suggests null association with relatively precise estimate.

Between-subgroup analysis: The variation across geographic regions was substantial, but formal tests of subgroup differences were not conducted due to the small number of studies per region and substantial within-subgroup heterogeneity.

Study Design Analysis

Appendix Figure 6: *Forest plot of odds ratios for the association between perceived childhood socioeconomic adversity and adult multimorbidity, showing individual study estimates and pooled random-effects meta-analysis. Black diamond represents the pooled odds ratio and 95% confidence interval. Squares represent study-specific odds ratios, with the size of each square proportional to the study's weight in the random-effects meta-analysis. Horizontal lines represent 95% confidence intervals for individual studies. Separated by study design*

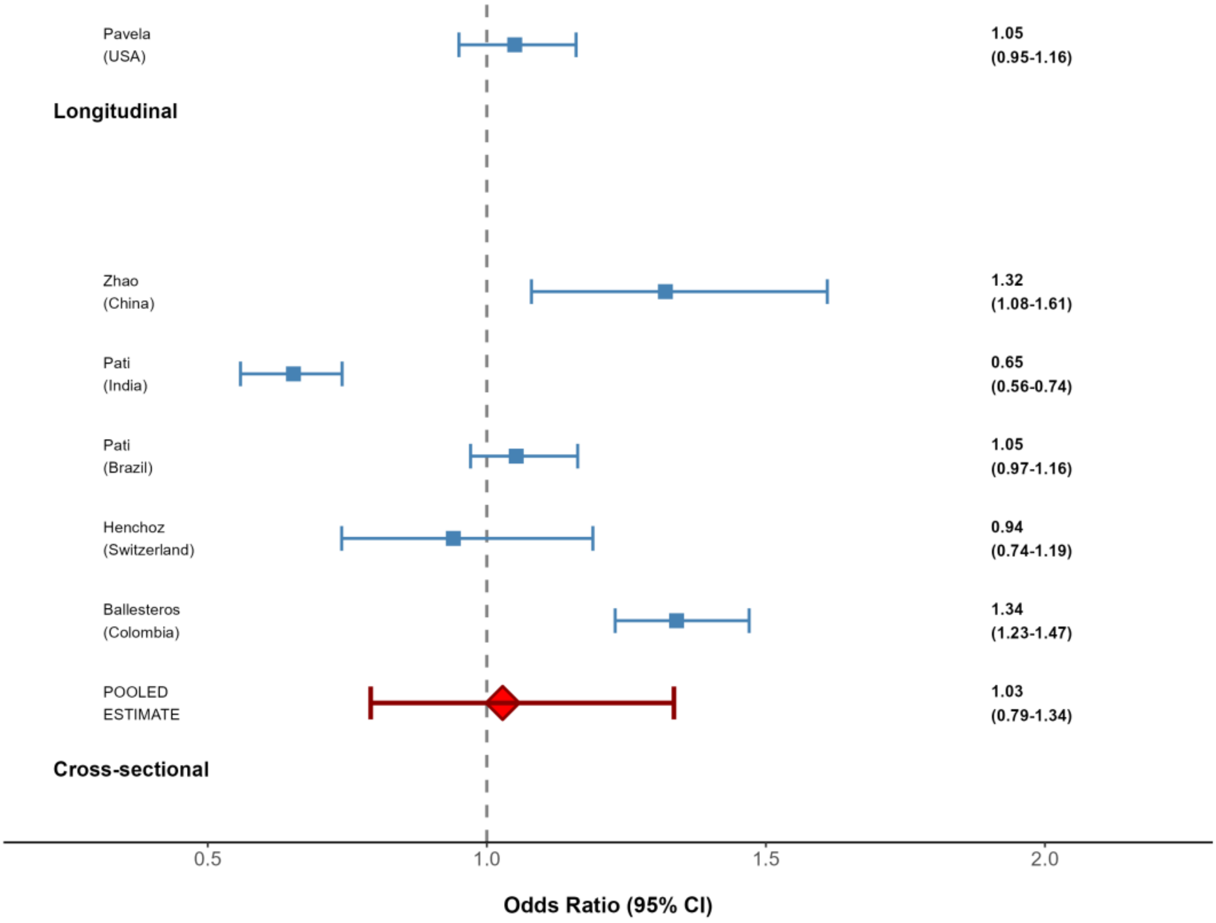

Perceived Childhood Economic Adversity by Study Design

Cross-sectional studies (n=5 studies)

- Pooled OR: 1.03 (95% CI: 0.79-1.34)
- I^2: 94.8% (very high heterogeneity)
- Studies: Ballesteros, Henchoz, Pati (India), Pati (Brazil), Zhao
- Interpretation: No clear association with substantial heterogeneity. Effect estimates varied considerably across cross-sectional studies from different contexts.

Longitudinal studies (n=1 study)

- OR: 1.05 (95% CI: 0.95-1.16)
- Study: Pavela et al. (USA)
- Interpretation: Single longitudinal study shows null association. The similarity between cross-sectional pooled estimate and the longitudinal study suggests study design may not be a major source of heterogeneity, though this comparison is limited by having only one longitudinal study.

Multimorbidity Definition Analysis

Perceived Childhood Economic Adversity by Multimorbidity Definition

Standard definition (≥2 chronic conditions) (n=5 studies)

- Pooled OR: 1.03 (95% CI: 0.79-1.34)
- I^2: 94.8% (very high heterogeneity)
- Studies: Ballesteros, Henchoz, Pati (India), Pati (Brazil), Zhao
- Interpretation: No clear association despite using standardized outcome definition. High heterogeneity persists even with consistent outcome measurement.

Alternative definition (continuous measure) (n=1 study)

- OR: 1.05 (95% CI: 0.95-1.16)
- Study: Pavela et al. (USA) - used number of chronic conditions as continuous outcome
- Interpretation: Single study using continuous outcome shows similar null finding to studies using binary definition.

Exposure Type Comparison

Appendix figure 7: Forest plot of odds ratios for the association between childhood socioeconomic adversity and adult multimorbidity, stratified by exposure type. Diamonds represent pooled random-effects meta-analysis estimates with 95% confidence intervals (CIs) for each exposure category (e.g. perceived economic circumstances, parental education, parental occupation). Horizontal lines indicate 95% CIs for each pooled estimate. All exposures were coded such that higher adversity reflects lower socioeconomic conditions in childhood. The size of each diamond reflects the number of studies contributing to that exposure category.

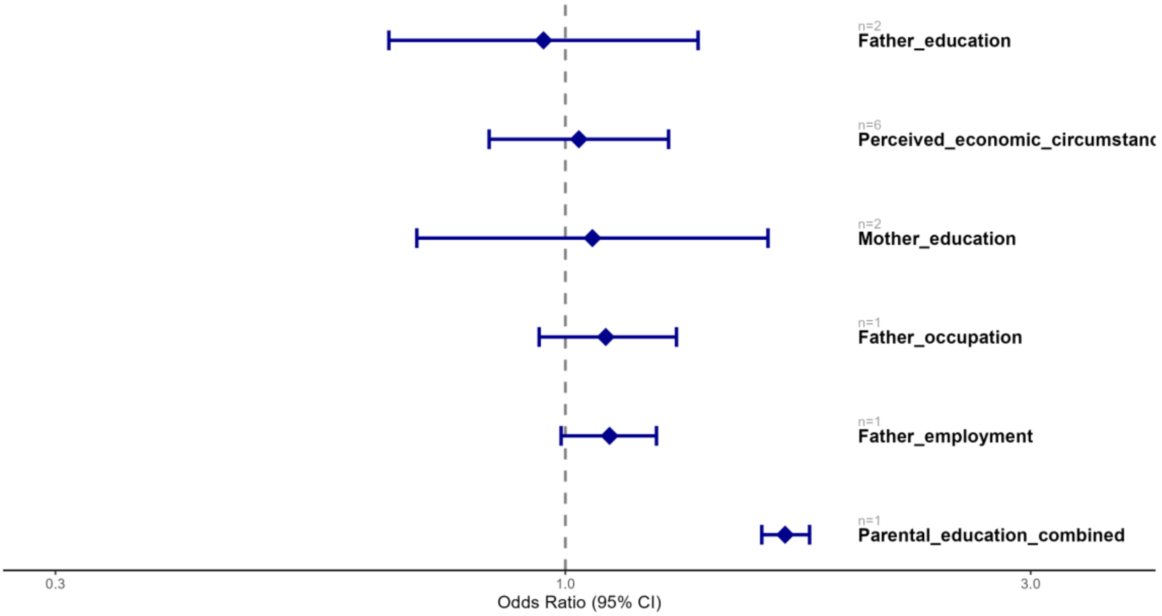

All Exposure Types

Perceived childhood economic circumstances (n=6 studies)

- Pooled OR: 1.03 (95% CI: 0.84-1.28)
- I^2: 93.5% (very high heterogeneity)
- Interpretation: Most extensively studied exposure type. No clear association with very high heterogeneity across diverse geographic and methodological contexts.

Father's education (n=2 studies)

- Pooled OR: 0.95 (95% CI: 0.66-1.37)
- I^2: 66.8% (moderate-high heterogeneity)
- Studies: Zhao et al. (China), Pavela et al. (USA)
- Interpretation: Suggests potential protective association but with substantial uncertainty. Moderate heterogeneity between studies from different contexts.

Mother's education (n=2 studies)

- Pooled OR: 1.07 (95% CI: 0.70-1.61)
- I^2: 36.9% (moderate heterogeneity)
- Studies: Zhao et al. (China), Pavela et al. (USA)
- Interpretation: Null association with lower heterogeneity than father's education, though still imprecise.

Combined parental education (n=1 study)

- OR: 1.68 (95% CI: 1.59-1.78)
- Study: Schramm et al. (Denmark)
- Interpretation: Strongest individual association observed across all analyses. Large, precise effect estimate from high-quality Danish registry data.

Father's employment status (n=1 study)

- OR: 1.11 (95% CI: 0.99-1.24)
- Study: Pavela et al. (USA)
- Interpretation: Suggests possible harmful association but confidence interval includes null.

Sensitivity Analyses

Leave-One-Out Analysis for perceived childhlood economic circumsatnces

The leave-one-out sensitivity analysis for perceived childhood economic circumstances revealed:

- Excluding Ballesteros et al. (Colombia): OR 0.97 (95% CI: 0.78-1.21), I^2 = 94.1%
- Excluding Henchoz et al. (Switzerland): OR 1.06 (95% CI: 0.84-1.34), I^2 = 94.7%
- Excluding Pati et al. (India): OR 1.14 (95% CI: 1.00-1.29), I^2 = 81.6%
- Excluding Pati et al. (Brazil): OR 1.02 (95% CI: 0.80-1.31), I^2 = 95.3%
- Excluding Pavela et al. (USA): OR 1.02 (95% CI: 0.79-1.33), I^2 = 95.4%
- Excluding Zhao et al. (China): OR 0.98 (95% CI: 0.81-1.19), I^2 = 93.8%

Key findings:

- No single study exerted disproportionate influence on the overall null association
- Excluding Pati et al. (India) had the largest impact, shifting the pooled estimate upward and notably reducing heterogeneity
- The Indian study appears to be a key contributor to both the null finding and the observed heterogeneity

Summary of Subgroup Findings

Key Observations

1. **Geographic heterogeneity**: Substantial variation across regions, with opposing effects observed within Asia (protective in India, harmful in China) and consistent harmful trends in Latin America.
2. **Study design consistency**: Limited evidence suggests study design may not be a major source of heterogeneity, though this is based on comparison with only one longitudinal study.
3. **Multimorbidity definition**: Standardizing outcome definitions did not reduce heterogeneity, suggesting that variation in multimorbidity measurement is not the primary driver of inconsistent findings.
4. **Exposure type variation**: Different exposure types showed varying patterns, with perceived economic circumstances showing the highest heterogeneity and combined parental education showing the strongest individual association.
5. **Persistent heterogeneity**: High heterogeneity persisted across most subgroups, indicating that the pre-specified subgroup variables do not fully explain the observed variation between studies.

Implications

The subgroup analyses reveal that heterogeneity in this evidence base is not readily explained by the study characteristics examined. The persistence of high I^2 values across subgroups suggests that other factors, potentially including unmeasured contextual variables, differences in population characteristics, varying social policies, or methodological heterogeneity not captured by our subgroup variables, may be driving the observed inconsistency.

The substantial geographic variation, particularly the opposing effects observed within Asia, highlights the importance of context in understanding these associations. This suggests that the relationship between childhood socioeconomic disadvantage and adult multimorbidity may vary significantly across different social, economic, and healthcare contexts.

The finding that the Danish registry study (Schramm et al.) showed the strongest association, combined with its high methodological quality, suggests that when measured accurately with appropriate follow-up, associations between childhood disadvantage and adult multimorbidity may be more consistent and substantial than suggested by the predominantly cross-sectional literature relying on retrospective exposure measurement.

Limitations of Subgroup Analyses

Several limitations must be acknowledged:

1. Small sample sizes: Most subgroups contained only 1-2 studies, limiting statistical power and interpretability of subgroup comparisons.

2. Overlapping characteristics: Studies differed across multiple dimensions simultaneously, making it difficult to isolate the effect of any single characteristic.

3. Residual confounding: Unmeasured study characteristics may explain more variation than the characteristics examined.

4. I^2 instability: With few studies per subgroup, I^2 statistics may be unstable and should be interpreted cautiously.

5. Lack of individual participant data: Aggregate-level subgroup analyses may miss important patient-level effect modifiers.

These limitations reinforce the tentative nature of the subgroup findings and the need for larger, more methodologically homogeneous studies to clarify sources of heterogeneity in this field.

Appendix L: Defining effect size thresholds for certainty judgements using the GRADE Evidence to Decision Framework

Context: This document outlines predefined thresholds for interpreting the magnitude of effect sizes on the relative scale (odds ratios), applied in the context of a systematic review examining the relationship between childhood socioeconomic conditions (SEC) and adult multimorbidity.

Purpose: To ensure consistency and transparency in GRADE certainty assessments for the domain of imprecision, by applying a structured threshold approach derived from the GRADE Evidence to Decision (EtD) framework.

1. Decision Framework Assumptions:

- The outcome of interest (adult multimorbidity) is binary and socially and clinically important.
- The review reports pooled odds ratios (ORs) as the effect measure.
- Judgements are intended to inform policy and public health priorities related to early-life disadvantage and long-term health outcomes.

2. Odds Ratio Thresholds (Relative Effect Scale):

These thresholds are adapted from GRADE EtD guidance and contextualised to reflect relative differences in the odds of multimorbidity:

Effect Size Category	Odds Ratio (OR) Range	Interpretation
No or trivial effect	0.95 to 1.05	Unlikely to be meaningful for policy
Small effect	0.90–0.94 or 1.06–1.10	May influence specific decisions
Moderate effect	0.80–0.89 or 1.11–1.25	Likely to influence decisions
Large effect	<0.80 or >1.25	Highly likely to influence decisions

Note: OR <1 implies a protective effect; OR >1 implies harm. Thresholds apply symmetrically.

3. Threshold Use in Certainty Judgements (Imprecision Domain):

- 0 thresholds crossed: Do not rate down.
- 1 threshold crossed: Rate down by 1 level (serious imprecision).
- 2 thresholds crossed: Rate down by 2 levels (very serious).
- 3+ thresholds crossed: Rate down by 3 levels (extremely serious).

4. Justification of Threshold Choice:

The chosen OR thresholds reflect:

- The epidemiological importance of adult multimorbidity.
- The expected range of plausible relative effects in population studies.
- The need to distinguish between statistically detectable effects and those likely to matter to stakeholders (patients, practitioners, policymakers).

They are consistent with GRADE's guidance and adapted to the public health domain.

5. Application Note:

When applying these thresholds:

- Ensure consistent use of odds ratios across all exposure domains (e.g., parental education, perceived economic conditions).
- Convert CIs from absolute to relative effects only when necessary, using consistent baseline prevalence if needed for auxiliary interpretation.
- Document any deviation from the thresholds with explicit rationale.

Appendix M – Absolute Risk Difference Calculations for GRADE

Following GRADE guidance, we calculated absolute risk differences to support interpretability of findings and inform decision-making. Because odds ratios (ORs) can overestimate risk—especially when outcomes are common—we first converted pooled ORs to approximate risk ratios (RRs) using the formula:

$$RR = OR / ((1 - P0) + (P0 \times OR))$$

Where P0 is the assumed baseline risk of multimorbidity in the unexposed (reference) group. This baseline risk was derived from external prevalence estimates (Chowdhury et al., 2023). The resulting RR was then applied to the baseline risk to estimate the absolute risk in the exposed group, allowing calculation of the risk difference.

This method ensures that certainty judgements under GRADE for imprecision are tied to meaningful, population-relevant effect sizes on both relative and absolute scales.

Appendix N - R code used to generate plots

Forest plot code

```
# Load required libraries
library(readxl)
library(dplyr)
library(tidyr)
library(stringr)
library(ggplot2)
library(metafor)

# === 1. Read and preprocess data ===
data <- read_excel("your_data.xlsx")
names(data) <- make.names(names(data))

if (!"Adjustment" %in% names(data)) {
  data$Adjustment <- case_when(
    str_detect(data$Study, "Ballesteros") ~ "Underadjusted",
    str_detect(data$Study, "Dekhtyar")   ~ "Underadjusted",
    str_detect(data$Study, "Haas")       ~ "Appropriately adjusted",
    str_detect(data$Study, "Henchoz")    ~ "Appropriately adjusted",
    str_detect(data$Study, "Keetile")    ~ "Overadjusted",
    str_detect(data$Study, "Pati")       ~ "Underadjusted",
    str_detect(data$Study, "Pavela")     ~ "Appropriately adjusted",
    str_detect(data$Study, "Putnam")     ~ "Underadjusted",
    str_detect(data$Study, "Schramm")    ~ "Underadjusted",
    str_detect(data$Study, "Zhao")       ~ "Underadjusted",
    TRUE                                 ~ "Unknown"
  )
}

data <- data %>%
  mutate(
    OR                       = as.numeric(OR),
    CI_lower                 = as.numeric(Lower_95._CI_Limit),
    CI_upper                 = as.numeric(Upper_95._CI_Limit),
    Exposure_Characterisation = trimws(Exposure.characterisation),
    adjustment_superscript    = case_when(
      Adjustment == "Underadjusted" ~ "ᵃ",
      Adjustment == "Overadjusted"  ~ "ᵇ",
      TRUE                          ~ ""
    ),
    log_OR   = log(OR),
    log_low  = log(CI_lower),
    log_high = log(CI_upper),
    SE_calc  = (log_high - log_low) / (2 * 1.96)
  ) %>%
  mutate(
    outcomegroup = str_to_lower(trimws(outcomegroup)),
    Subgroup = case_when(
```

```
    outcomegroup == "father's education"             ~ "Father's education",
    outcomegroup == "mother's education"             ~ "Mother's education",
    outcomegroup == "parental education"             ~ "Parental education",
    outcomegroup == "perceived economic circumstances" ~ "Perceived economic circumstances",
    TRUE                                   ~ NA_character_
  )
 ) %>%
 filter(!is.na(Subgroup), !is.na(OR), !is.na(CI_lower), !is.na(CI_upper))

# === 2. Meta-analysis helper ===
pool_meta <- function(df, subgroup_name) {
 res <- rma(yi = log_OR, sei = SE_calc, data = df, method = "REML")
 weights_df <- data.frame(
  Study_Label = df$Study,
  Weight     = weights(res) / sum(weights(res)) * 100,
  Subgroup   = subgroup_name
 )
 pooled_row <- data.frame(
  Study_Label           = paste0("Pooled estimate: ", subgroup_name),
  Subgroup              = subgroup_name,
  OR                    = exp(res$b),
  CI_lower              = exp(res$ci.lb),
  CI_upper              = exp(res$ci.ub),
  SE_calc               = NA,
  RoB                   = NA,
  Exposure_Characterisation = "Summary estimate",
  adjustment_superscript   = "",
  pooled                = TRUE,
  Weight                = NA
 )
 list(model = res, weights = weights_df, pooled = pooled_row)
}

# === 3. Split and run meta-analyses ===
father_df   <- data %>% filter(Subgroup == "Father's education") %>% distinct(Study, .keep_all =
TRUE)
mother_df   <- data %>% filter(Subgroup == "Mother's education") %>% distinct(Study, .keep_all =
TRUE)
parental_df <- data %>% filter(Subgroup == "Parental education") %>% distinct(Study, .keep_all =
TRUE)
perc_df     <- data %>% filter(outcomegroup == "perceived economic circumstances") %>%
distinct(Study, .keep_all = TRUE)

father_meta   <- if (nrow(father_df)   > 1) pool_meta(father_df,   "Father's education")            else
NULL
mother_meta   <- if (nrow(mother_df)   > 1) pool_meta(mother_df,   "Mother's education")
else NULL
parental_meta <- if (nrow(parental_df) > 1) pool_meta(parental_df, "Parental education")
else NULL
perc_meta     <- if (nrow(perc_df)     > 1) pool_meta(perc_df,     "Perceived economic circumstances")
else NULL
```

```
# === 4. Combine weights and pooled rows ===
data_weighted <- bind_rows(
  if (!is.null(father_meta))   left_join(father_df,   father_meta$weights, by = c("Study" =
"Study_Label", "Subgroup")) else father_df   %>% mutate(Weight = NA),
  if (!is.null(mother_meta))   left_join(mother_df,   mother_meta$weights, by = c("Study" =
"Study_Label", "Subgroup")) else mother_df   %>% mutate(Weight = NA),
  if (!is.null(perc_meta))     left_join(perc_df,     perc_meta$weights,   by = c("Study" = "Study_Label",
"Subgroup")) else perc_df     %>% mutate(Weight = NA),
  parental_df %>% mutate(Weight = NA)
)

pooled_rows <- bind_rows(
  if (!is.null(father_meta)) father_meta$pooled else NULL,
  if (!is.null(mother_meta)) mother_meta$pooled else NULL,
  if (!is.null(perc_meta))   perc_meta$pooled   else NULL
)

plot_data_all <- data_weighted %>%
  mutate(Study_Label = Study, pooled = FALSE) %>%
  select(Study_Label, Subgroup, OR, CI_lower, CI_upper, SE_calc, RoB,
         Exposure_Characterisation, adjustment_superscript, pooled, Weight) %>%
  bind_rows(if (nrow(pooled_rows) > 0) pooled_rows else NULL) %>%
  mutate(
    OR_CI_Label  = sprintf("%.2f (%.2f–%.2f)", OR, CI_lower, CI_upper),
    RoB_Label    = case_when(
      is.na(pooled) | pooled ~ "",
      RoB == "High"          ~ "High",
      RoB == "Very High"     ~ "Very High",
      TRUE                   ~ as.character(RoB)
    ),
    RoB_Label    = paste0(RoB_Label, adjustment_superscript),
    Weight_Label = ifelse(is.na(Weight) | Weight == 0, "", sprintf("%.1f%%", Weight)),
    is_pooled    = grepl("^Pooled", Study_Label)
  )

plot_data_edu  <- filter(plot_data_all, Subgroup %in% c("Father's education", "Mother's education",
"Parental education"))
plot_data_perc <- filter(plot_data_all, Subgroup == "Perceived economic circumstances")

# === 5. Forest-plot function ===
plot_forest <- function(df, title_text, use_facet = TRUE, show_footnote = TRUE) {
  maxw <- max(df$CI_upper, na.rm = TRUE)
  if (use_facet) {
    x1 <- maxw * 1.1; x2 <- maxw * 1.7; x3 <- maxw * 2.3; x4 <- maxw * 3.0
  } else {
    x1 <- maxw * 1.3; x2 <- maxw * 1.8; x3 <- maxw * 2.2; x4 <- maxw * 2.8
  }

  df2 <- df %>%
    mutate(is_pooled = grepl("^Pooled", Study_Label)) %>%
```

```
  group_by(Subgroup) %>%
  arrange(Subgroup, is_pooled, Study_Label, .by_group = TRUE) %>%
  mutate(y_pos = rev(row_number())) %>%
  ungroup()

hasW <- any(!is.na(df2$Weight) & df2$Weight != "")

hdr <- if (hasW) {
  data.frame(x = c(x1, x2, x3, x4), y = max(df2$y_pos) + 1,
             label = c("OR (95% CI)", "RoB", "Weight", "Exposure"),
             Subgroup = df2$Subgroup[1])
} else {
  data.frame(x = c(x1, x2, x4), y = max(df2$y_pos) + 1,
             label = c("OR (95% CI)", "RoB", "Exposure"),
             Subgroup = df2$Subgroup[1])
}

p <- ggplot(df2, aes(x = OR, y = y_pos)) +
  geom_vline(xintercept = 1, linetype = "dashed", colour = "grey60", size = 0.7) +
  geom_point(data = subset(df2, !is_pooled), shape = 15, size = 2.5, colour = "black") +
  geom_errorbarh(data = subset(df2, !is_pooled),
                 aes(xmin = CI_lower, xmax = CI_upper),
                 height = 0.08, colour = "black") +
  geom_point(data = subset(df2, is_pooled), shape = 23, size = 4, fill = "black", colour = "black") +
  geom_errorbarh(data = subset(df2, is_pooled),
                 aes(xmin = CI_lower, xmax = CI_upper),
                 height = 0.08, colour = "black") +
  geom_text(aes(label = OR_CI_Label, x = x1), hjust = 0, size = 3.2) +
  geom_text(aes(label = RoB_Label, x = x2), hjust = 0, size = 3.2) +
  geom_text(aes(label = Exposure_Characterisation, x = x4), hjust = 0, size = 3.2)

if (hasW) {
  p <- p + geom_text(aes(label = Weight_Label, x = x3), hjust = 0, size = 3.2)
}

if (use_facet) {
  p <- p + facet_grid(Subgroup ~ ., switch = "y", scales = "free_y", space = "fixed") +
    geom_text(data = hdr, aes(x = x, y = y, label = label),
              inherit.aes = FALSE, hjust = 0, vjust = 0.5,
              fontface = "bold", size = 3.8)
} else {
  p <- p + geom_text(data = hdr, aes(x = x, y = y, label = label),
                     inherit.aes = FALSE, hjust = 0, vjust = 0.5,
                     fontface = "bold", size = 3.8)
}

p + theme_bw() +
  theme(
    panel.grid.major.y = element_blank(),
    panel.grid.minor   = element_blank(),
    panel.border       = element_rect(color = "grey80"),
```

```
    strip.placement.y  = "outside",
    strip.text.y.right = element_text(size = 11, face = "bold", angle = 0, margin = margin(l = 10, r = 
15)),
    strip.background   = element_rect(fill = "grey95"),
    axis.text.y        = element_text(size = 9, hjust = 1, margin = margin(r = 18)),
    axis.text.x        = element_text(size = 9),
    axis.title.x       = element_text(size = 11, face = "bold", margin = margin(t = 10)),
    plot.title         = element_text(size = 12, face = "bold", hjust = 0.5),
    legend.position    = "none",
    panel.spacing      = unit(1.5, "lines"),
    plot.margin        = margin(35, 40, 10, 110)
  ) +
  labs(
    x      = "Odds Ratio",
    y      = NULL,
    title   = title_text,
    caption = "Black diamond represents the pooled odds ratio and 95% confidence interval. Squares 
represent study-specific odds ratios, with the size of each square proportional to study weight. 
Horizontal lines represent 95% confidence intervals. RoB: Risk of Bias."
  ) +
  scale_x_continuous(breaks = scales::pretty_breaks(n = 6), expand = expansion(mult = c(0.05, 1)))
}

# === 6. Generate and save plots ===
education_plot <- plot_forest(plot_data_edu, "Odds of Multimorbidity by Parental Education", 
use_facet = TRUE)
perceived_plot <- plot_forest(plot_data_perc, "Odds of Multimorbidity by Perceived Childhood 
Economic Circumstances", use_facet = FALSE)

edu_height  <- max(6, 2.5 + 0.4 * nrow(plot_data_edu))
perc_height <- max(4, 2.5 + 0.4 * nrow(plot_data_perc))

ggsave("education_forest_plot.png", education_plot, width = 16, height = edu_height, dpi = 300)
ggsave("perceived_forest_plot.png", perceived_plot, width = 16, height = perc_height, dpi = 300)

print(education_plot)
print(perceived_plot)
```

Leave one out plot

```
# Load required packages
library(metafor)
library(dplyr)
library(ggplot2)
library(forcats)

# === 1. Full model (no studies excluded) ===

full_model <- rma(yi = log_OR, sei = SE_calc, data = perc_df, method = "REML")
full_row <- data.frame(
  Excluded_Study = "None (Full model)",
```

```
  OR = exp(full_model$b),
  CI_lower = exp(full_model$ci.lb),
  CI_upper = exp(full_model$ci.ub),
  I2 = full_model$I2
)

# === 2. Perform leave-one-out analyses ===

leave_one_out_results <- list()
perc_studies <- unique(perc_df$Study)

for (excluded_study in perc_studies) {
  temp_df <- perc_df %>% filter(Study != excluded_study)
  meta_res <- rma(yi = log_OR, sei = SE_calc, data = temp_df, method = "REML")
  pooled_row <- data.frame(
    Excluded_Study = excluded_study,
    OR = exp(meta_res$b),
    CI_lower = exp(meta_res$ci.lb),
    CI_upper = exp(meta_res$ci.ub),
    I2 = meta_res$I2
  )
  leave_one_out_results[[excluded_study]] <- list(
    model = meta_res,
    pooled = pooled_row
  )
}

# === 3. Combine results and prepare table ===

loo_table <- do.call(rbind, lapply(leave_one_out_results, function(x) x$pooled))
loo_table <- rbind(full_row, loo_table)
loo_table <- loo_table %>% mutate(across(where(is.numeric), round, 3)) %>%
  mutate(
    Label = paste0("Excluded: ", Excluded_Study, "\nI² = ", sprintf("%.1f%%", I2)),
    OR_CI_Label = sprintf("%.2f (%.2f–%.2f)", OR, CI_lower, CI_upper)
  )

print(loo_table)

# === 4. Create forest plot ===

loo_table <- loo_table %>%
  arrange(desc(OR)) %>%
  mutate(Label = fct_inorder(Label))

loo_plot <- ggplot(loo_table, aes(x = OR, y = Label)) +
  geom_vline(xintercept = 1, linetype = "dashed", colour = "grey60") +
  geom_point(shape = 23, size = 4, fill = "black") +
  geom_errorbarh(aes(xmin = CI_lower, xmax = CI_upper),
               height = 0.3, linewidth = 1, color = "black") +
  geom_text(aes(label = OR_CI_Label), x = 1.5, hjust = 0, size = 3.5, color = "black") +
```

```
  theme_bw() +
  labs(
    title = "Leave-One-Out Meta-Analysis: Perceived Childhood Economic Circumstances",
    x = "Pooled Odds Ratio",
    y = NULL,
    caption = "Each point represents the pooled estimate excluding the indicated study (top row: full
model)."
  ) +
  scale_x_continuous(limits = c(min(loo_table$CI_lower), 1.75)) +
  theme(
    axis.text.y = element_text(size = 9),
    axis.title.x = element_text(size = 11, face = "bold", margin = margin(t = 10)),
    plot.title = element_text(size = 12, face = "bold", hjust = 0.5),
    plot.caption = element_text(hjust = 0, size = 8, face = "italic", color = "grey30"),
    plot.margin = margin(30, 120, 10, 5)
  )

# === 5. Save and display ===

ggsave("leave_one_out_plot.png",
       loo_plot,
       width = 13.5,
       height = 0.5 * nrow(loo_table) + 2,
       dpi = 300)

loo_plot

# === 6. Output table for reporting ===

loo_print_table <- loo_table %>%
  select(Excluded_Study, OR, CI_lower, CI_upper, I2) %>%
  mutate(
    `95% CI` = sprintf("%.2f–%.2f", CI_lower, CI_upper),
    OR = sprintf("%.2f", OR),
    `I²` = sprintf("%.1f%%", I2)
  ) %>%
  select(`Excluded Study` = Excluded_Study, `OR` = OR, `95% CI`, `I²`)

print(loo_print_table, row.names = FALSE)

# === 7. Summary statistics ===

cat("\n=== SUMMARY ===\n")
cat(sprintf("Range of ORs: %.2f – %.2f\n",
            min(loo_table$OR), max(loo_table$OR)))
cat(sprintf("Range of I²: %.1f%% – %.1f%%\n",
            min(loo_table$I2), max(loo_table$I2)))
cat(sprintf("Number of studies: %d\n", length(perc_studies)))
```

RII Code

```
# Load required packages
library(dplyr)
library(readxl)
library(broom)
library(ggplot2)
library(knitr)
library(kableExtra)

# === 1. Load and clean data ===
data <- read_excel("rii_input.xlsx")

data <- data %>%
  mutate(
    `Lower 95% CI Limit` = as.numeric(gsub("[^0-9\\.]", "", `Lower 95% CI Limit`)),
    `Upper 95%CI Limit`  = as.numeric(gsub("[^0-9\\.]", "", `Upper 95%CI Limit`)),
    OR        = as.numeric(OR),
    SE        = as.numeric(SE),
    MM_cases   = as.numeric(MM_cases),
    Group_N    = as.numeric(Group_N),
    Ridit_Score = as.numeric(Ridit_Score),
    SE = ifelse(!is.na(OR) & !is.na(`Upper 95%CI Limit`) & !is.na(`Lower 95% CI Limit`) & is.na(SE),
            (log(`Upper 95%CI Limit`) - log(`Lower 95% CI Limit`)) / (2 * 1.96),
            SE),
    Study_Exposure = Study
  )

# === 2. Helper: two-point slope calculation ===
calculate_two_point_rii <- function(study_data, study_name) {
  model_data <- study_data %>%
    filter(!is.na(OR) & !is.na(SE) & OR != 1) %>%
    arrange(Ridit_Score)
  if (nrow(model_data) != 2) return(NULL)

  point1 <- model_data[1,]; point2 <- model_data[2,]
  log_or1 <- log(point1$OR); log_or2 <- log(point2$OR)
  ridit1 <- point1$Ridit_Score; ridit2 <- point2$Ridit_Score
  se1 <- point1$SE; se2 <- point2$SE

  slope <- (log_or2 - log_or1) / (ridit2 - ridit1)
  var_slope <- (se1^2 + se2^2) / (ridit2 - ridit1)^2
  se_slope <- sqrt(var_slope)

  rii <- exp(slope)
  rii_ci <- exp(c(slope - 1.96 * se_slope, slope + 1.96 * se_slope))

  data.frame(
    Study = study_name,
    Method = "WLS on log(OR)",
    RII = round(rii, 3),
    CI_lower = round(rii_ci[1], 3),
```

```
    CI_upper = round(rii_ci[2], 3),
    N_groups = nrow(study_data)
  )
}

# === 3. Main function for RII calculation ===
calculate_rii <- function(study_name, data, primary_analysis = TRUE) {
  study_data <- data %>% filter(Study_Exposure == study_name)
  if (nrow(study_data) == 0) return(NULL)

  n_groups <- nrow(study_data)
  non_ref_or_count <- sum(!is.na(study_data$OR) & study_data$OR != 1)
  non_ref_or_se_count <- sum(!is.na(study_data$OR) & !is.na(study_data$SE) & study_data$OR !=
1)
  complete_binomial_data <- sum(!is.na(study_data$MM_cases) & !is.na(study_data$Group_N))

  # === Primary analysis (robust methods only) ===
  if (primary_analysis) {
    # Method 1: Binomial GLM
    if (complete_binomial_data == n_groups && n_groups >= 3) {
      model <- glm(cbind(MM_cases, Group_N - MM_cases) ~ Ridit_Score,
                   data = study_data, family = binomial())
      est <- coef(model)[["Ridit_Score"]]
      ci <- tryCatch(confint(model, parm = "Ridit_Score", level = 0.95),
                     error = function(e) {
                       se_est <- summary(model)$coefficients["Ridit_Score", "Std. Error"]
                       c(est - 1.96 * se_est, est + 1.96 * se_est)
                     })
      rii <- exp(est); rii_ci <- exp(ci)
      return(data.frame(
        Study = study_name, Method = "Binomial GLM",
        RII = round(rii, 3), CI_lower = round(rii_ci[1], 3),
        CI_upper = round(rii_ci[2], 3), N_groups = n_groups))
    }
    # Method 2: WLS on log(OR)
    if (non_ref_or_se_count >= 2) {
      model_data <- study_data %>%
        filter(!is.na(OR) & !is.na(SE) & OR != 1 & OR > 0 & SE > 0) %>%
        mutate(LogOR = log(OR), weight = 1 / (SE^2))
      if (nrow(model_data) == 2) {
        result <- calculate_two_point_rii(study_data, study_name)
        if (!is.null(result)) return(result)
      } else if (nrow(model_data) > 2 && var(model_data$Ridit_Score) > 1e-10) {
        model <- lm(LogOR ~ Ridit_Score, data = model_data, weights = weight)
        estimate <- coef(model)["Ridit_Score"]
        se_est <- summary(model)$coefficients["Ridit_Score", "Std. Error"]
        rii <- exp(estimate)
        rii_ci <- exp(c(estimate - 1.96 * se_est, estimate + 1.96 * se_est))
        return(data.frame(
          Study = study_name, Method = "WLS on log(OR)",
          RII = round(rii, 3), CI_lower = round(rii_ci[1], 3),
```

```
      CI_upper = round(rii_ci[2], 3), N_groups = n_groups))
    }
  }
  return(NULL)
}

# === Sensitivity analysis (all methods) ===
if (!primary_analysis) {
  # Method 1: Binomial GLM
  if (complete_binomial_data == n_groups) {
    model <- glm(cbind(MM_cases, Group_N - MM_cases) ~ Ridit_Score,
                 data = study_data, family = binomial())
    est <- coef(model)[["Ridit_Score"]]
    ci <- tryCatch(confint(model, parm = "Ridit_Score", level = 0.95),
                   error = function(e) {
                     se_est <- summary(model)$coefficients["Ridit_Score", "Std. Error"]
                     c(est - 1.96 * se_est, est + 1.96 * se_est)
                   })
    rii <- exp(est); rii_ci <- exp(ci)
    return(data.frame(
      Study = study_name, Method = "Binomial GLM",
      RII = round(rii, 3), CI_lower = round(rii_ci[1], 3),
      CI_upper = round(rii_ci[2], 3), N_groups = n_groups))
  }
  # Method 2: WLS on log(OR)
  if (non_ref_or_se_count >= 2) {
    model_data <- study_data %>%
      filter(!is.na(OR) & !is.na(SE) & OR != 1 & OR > 0 & SE > 0) %>%
      mutate(LogOR = log(OR), weight = 1 / (SE^2))
    if (nrow(model_data) == 2) {
      result <- calculate_two_point_rii(study_data, study_name)
      if (!is.null(result)) return(result)
    } else if (nrow(model_data) > 2 && var(model_data$Ridit_Score) > 1e-10) {
      model <- lm(LogOR ~ Ridit_Score, data = model_data, weights = weight)
      estimate <- coef(model)["Ridit_Score"]
      se_est <- summary(model)$coefficients["Ridit_Score", "Std. Error"]
      rii <- exp(estimate)
      rii_ci <- exp(c(estimate - 1.96 * se_est, estimate + 1.96 * se_est))
      return(data.frame(
        Study = study_name, Method = "WLS on log(OR)",
        RII = round(rii, 3), CI_lower = round(rii_ci[1], 3),
        CI_upper = round(rii_ci[2], 3), N_groups = n_groups))
    }
  }
  # Method 3: Single OR extrapolation
  if (non_ref_or_count == 1) {
    ref_row <- study_data %>% filter(OR == 1 | is.na(OR)) %>% slice(1)
    non_ref_row <- study_data %>% filter(!is.na(OR) & OR != 1) %>% slice(1)
    ridit_diff <- abs(non_ref_row$Ridit_Score - ref_row$Ridit_Score)
    if (!is.na(ridit_diff) && ridit_diff > 0 && !is.na(non_ref_row$OR) && non_ref_row$OR > 0) {
      log_rii <- log(non_ref_row$OR) / ridit_diff
```

```
      rii <- exp(log_rii)
      rii_ci <- if (!is.na(non_ref_row$SE) && non_ref_row$SE > 0) {
        c(exp(log_rii - 1.96 * (non_ref_row$SE / ridit_diff)),
          exp(log_rii + 1.96 * (non_ref_row$SE / ridit_diff)))
      } else c(NA, NA)
      return(data.frame(
        Study = study_name, Method = "Single OR extrapolation",
        RII = round(rii, 3), CI_lower = round(rii_ci[1], 3),
        CI_upper = round(rii_ci[2], 3), N_groups = n_groups))
    }
  }
 }
 return(NULL)
}

# === 4. Run analyses ===
study_exposures <- unique(data$Study_Exposure)
rii_results_primary <- do.call(rbind, lapply(study_exposures, calculate_rii, data = data,
primary_analysis = TRUE))
rii_results_sensitivity <- do.call(rbind, lapply(study_exposures, calculate_rii, data = data,
primary_analysis = FALSE))

# === 5. Plot results ===
create_aligned_forest_plot <- function(data, base_size = 10, title_suffix = "") {
  plot_data <- data %>%
    mutate(
      FinalGroup = case_when(
        grepl("Ballasteros", Study) ~ "Perceived childhood economic conditions",
        grepl("Henchoz", Study) ~ "Perceived childhood economic conditions",
        grepl("Pati", Study) ~ "Perceived childhood economic conditions",
        grepl("Putnam", Study) ~ "Parental occupation",
        grepl("Schramm", Study) ~ "Parental education",
        grepl("Dekhtyar", Study) ~ "Parental occupation",
        TRUE ~ "Other"
      ),
      RII_text = sprintf("%.2f (%.2f–%.2f)", RII, CI_lower, CI_upper)
    )
  ggplot(plot_data, aes(y = reorder(Study, RII))) +
    geom_vline(xintercept = 1, linetype = "dashed", color = "gray60") +
    geom_errorbarh(aes(xmin = CI_lower, xmax = CI_upper), height = 0.3, color = "black") +
    geom_point(aes(x = RII), color = "black", size = 3) +
    geom_text(aes(x = 6.5, label = RII_text), hjust = 0, size = 3) +
    facet_grid(FinalGroup ~ ., scales = "free_y", space = "free_y") +
    scale_x_continuous(trans = "log10", breaks = c(0.25, 0.5, 1, 2, 4, 8),
                       limits = c(0.1, 13),
                       name = "Relative Index of Inequality (95% CI)") +
    theme_minimal(base_size = base_size) +
    theme(panel.grid = element_blank()) +
    labs(title = paste0("Forest plot of Relative Index of Inequality", title_suffix))
}
```

```
if (nrow(rii_results_primary) > 0)
  ggsave("rii_primary_final.png",
         plot = create_aligned_forest_plot(rii_results_primary, title_suffix = " - Primary Analysis"),
         width = 12, height = 8, dpi = 300)

if (nrow(rii_results_sensitivity) > 0)
  ggsave("rii_sensitivity_final.png",
         plot = create_aligned_forest_plot(rii_results_sensitivity, title_suffix = " - Sensitivity Analysis"),
         width = 12, height = 8, dpi = 300)
```

Effect direction and Risk of Bias plot

```
library(dplyr)
library(gt)

# === 1. Create data frame ===
robins_e_data <- data.frame(
  study = c("Ballesteros (2021)", "Dekhtyar (2019)", "Haas (2008)",
            "Henchoz (2019)", "Keetile (2023)", "Pati & Sinha (Brazil)",
            "Pati & Sinha (India)", "Pavela (2016) - SES",
            "Pavela (2016) - Mother's edu", "Pavela (2016) - Father's edu",
            "Pavela (2016) - Father's emp", "Putnam (2013)", "Schramm",
            "Zhao - Econ", "Zhao - Father's edu", "Zhao - Mother's edu"),

  exposure_type = c("Perceived economic adversity", "Parental Occupation", "Composite",
                    "Perceived economic adversity", "Composite", "Perceived economic adversity",
                    "Perceived economic adversity", "Low childhood SES", "Mother's education <HS",
                    "Father's education <HS", "Father employment status", "Composite",
                    "Parental education", "Perceived economic adversity + Parental education",
                    "Father's education", "Mother's education"),

  d1 = c("NA", "NA", "High risk", "High risk", "Very high", "High", "High",
         "High", "High", "High", "High", "NA", "Very High", "High", "High", "High"),
  d2 = c("NA", "NA", "Some risk", "High risk", "High", "Some concerns", "Some concerns",
         "High", "High", "High", "High", "NA", "Some concerns", "Some concerns",
         "Some concerns", "Some concerns"),
  d3 = c("NA", "NA", "High risk", "Very high risk", "High", "High", "High",
         "Some concerns", "Some concerns", "Some concerns", "Some concerns", "NA",
         "Some concerns", "Some concerns", "Some concerns", "Some concerns"),
  d4 = c("NA", "NA", "Low risk", "Low risk", "Low", "Low", "Low", "Low", "Low",
         "Low", "Low", "NA", "Low", "Low", "Low", "Low"),
  d5 = c("NA", "NA", "High risk", "Very high risk", "High", "High", "High",
         "Some concerns", "Some concerns", "Some concerns", "Some concerns", "NA",
         "Some concerns", "Some concerns", "Some concerns", "Some concerns"),
  d6 = c("NA", "NA", "Low risk", "Low risk", "Low", "Low", "Low", "Some concerns",
         "Some concerns", "Some concerns", "Some concerns", "NA", "Some concerns",
         "Some concerns", "Some concerns", "Some concerns"),
  d7 = c("NA", "NA", "Low risk", "Low risk", "Some concerns", "High", "High",
         "Low", "Low", "Low", "Low", "NA", "Low", "Some concerns",
         "Some concerns", "Some concerns"),

  overall = c("Very high risk of bias", "Very high risk of bias", "High risk",
```

```
            "Very high risk of bias", "Very High", "High", "High", "High", "High",
            "High", "High", "Very high risk of bias", "Very high", "High",
            "Very High", "Very High"),

  or = c(1.34, 1.10, 1.025, 1.23, 1.78, 1.05, 0.65, 1.05, 1.19, 1.1, 1.11, 2.1,
       1.68, 1.3, 1.17, 1.01),
  lower_ci = c(1.23, 0.94, 0.91, 1.02, 1.11, 0.97, 0.56, 0.95, 1.0, 0.93, 0.99,
            1.8, 1.59, 1.2, 0.85, 0.84),
  upper_ci = c(1.47, 1.29, 1.05, 1.48, 2.6, 1.16, 0.76, 1.16, 1.41, 1.3, 1.24,
            2.45, 1.78, 1.4, 1.03, 1.22),
  effect_direction = c("Harm", "Equivocal", "Equivocal", "Harm", "Harm", "Equivocal",
                  "Benefit", "Equivocal", "Harm", "Equivocal", "Equivocal",
                  "Harm", "Harm", "Harm", "Equivocal", "Equivocal"),
  group = c("Perceived_economic_conditions", "Parental_occupation", "Composite",
          "Perceived_economic_conditions", "Composite", "Perceived_economic_conditions",
          "Perceived_economic_conditions", "Perceived_economic_conditions",
          "Parental_education", "Parental_education", "Parental_employment_status",
          "Composite", "Parental_education", "Perceived_economic_conditions",
          "Parental_education", "Parental_education")
)

# === 2. Colour mapping functions ===
color_cells <- function(x) {
  case_when(
    x %in% c("Low", "Low risk") ~ "#90EE90",
    x %in% c("Some concerns", "Some risk") ~ "#FFD700",
    x %in% c("High", "High risk") ~ "#FFA07A",
    x %in% c("Very high", "Very High", "Very high risk", "Very high risk of bias") ~ "#FF6347",
    x == "NA" ~ "#D3D3D3",
    TRUE ~ "white"
  )
}

format_direction_only <- function(direction) {
  case_when(
    direction == "Harm" ~ "↑",
    direction == "Benefit" ~ "↓",
    direction == "Equivocal" ~ "↔",
    TRUE ~ ""
  )
}

# === 3. Prepare table ===
robins_e_data$effect_direction_symbol <- sapply(robins_e_data$effect_direction,
format_direction_only)

robins_table <- robins_e_data %>%
  select(study, exposure_type, d1, d2, d3, d4, d5, d6, d7, overall, effect_direction_symbol) %>%
  gt() %>%
  cols_label(
    study = "Study",
```

```
    exposure_type = "Exposure Type",
    d1 = "D1", d2 = "D2", d3 = "D3", d4 = "D4", d5 = "D5", d6 = "D6", d7 = "D7",
    overall = "Overall", effect_direction_symbol = "Effect Direction"
  ) %>%
  tab_spanner(
    label = "ROBINS-E Risk of Bias Domains",
    columns = c(d1, d2, d3, d4, d5, d6, d7)
  ) %>%
  data_color(columns = d1:overall, colors = color_cells) %>%
  text_transform(
    locations = cells_body(columns = effect_direction_symbol),
    fn = function(x) {
      sapply(x, function(symbol) {
        if (symbol == "↑") {
          return("<span style='color:red; font-weight:bold; font-size:22px;'>↑</span>")
        } else if (symbol == "↓") {
          return("<span style='color:blue; font-weight:bold; font-size:22px;'>↓</span>")
        } else if (symbol == "↔") {
          return("<span style='color:gray; font-weight:bold; font-size:22px;'>↔</span>")
        }
        return(symbol)
      })
    }
  ) %>%
  tab_header(title = "Risk of Bias Assessment and Effect Direction") %>%
  tab_footnote(
    footnote = md("**Key to ROBINS-E domains:** D1: Confounding, D2: Exposure Measurement, D3: Participant Selection, D4: Post-Exposure Interventions, D5: Missing Data, D6: Outcome Measurement, D7: Selection of Reported Result."),
    locations = cells_column_spanners(spanners = "ROBINS-E Risk of Bias Domains")
  ) %>%
  tab_footnote(
    footnote = md("**Effect direction symbols:** ↑ = Harmful association, ↓ = Beneficial association, ↔ = Equivocal or null association."),
    locations = cells_column_labels(columns = effect_direction_symbol)
  ) %>%
  tab_source_note(
    source_note = html(
      "<strong>Legend:</strong>
      <div style='margin-top:6px'>
      <div style='display:inline-block;width:15px;height:15px;background-color:#90EE90;margin-right:5px;'></div>Low risk
      <div style='display:inline-block;width:15px;height:15px;background-color:#FFD700;margin:0 10px 0 10px;'></div>Some concerns
      <div style='display:inline-block;width:15px;height:15px;background-color:#FFA07A;margin-right:10px;'></div>High risk
      <div style='display:inline-block;width:15px;height:15px;background-color:#FF6347;margin-right:10px;'></div>Very high risk
      <div style='display:inline-block;width:15px;height:15px;background-color:#D3D3D3;margin-right:10px;'></div>Not assessed
      </div>"
```

```
    )
  ) %>%
  tab_source_note(
    source_note = md("**Note:** Studies rated 'Not fully assessed' were unadjusted or excluded at an early ROBINS-E screening stage and are classified as 'Very high risk of bias' overall.")
  ) %>%
  tab_options(table.width = pct(100), column_labels.font.weight = "bold")

# === 4. Save outputs ===
gt::gtsave(robins_table, "risk_of_bias_table.png", vwidth = 1000, vheight = 800)
```

Subgroup analysis

```
# === Load required libraries ===
library(readxl)
library(dplyr)
library(stringr)
library(metafor)
library(ggplot2)
library(cowplot)
library(knitr)
library(kableExtra)
library(purrr)

# === Load and prepare data ===
# The dataset should include columns:
# Study_ID, First_author, Country, Exposure_type, OR, Lower_95_CI_Limit, Upper_95_CI_Limit,
# Sample_size, Geographic_region, Study_design, MM_definition, etc.

data <- read_excel("sganalysis.xlsx")

# Calculate effect size measures
data <- data %>%
  mutate(
    OR = as.numeric(OR),
    Lower_95_CI_Limit = as.numeric(Lower_95_CI_Limit),
    Upper_95_CI_Limit = as.numeric(Upper_95_CI_Limit),
    Sample_size = as.numeric(Sample_size),
    log_OR = log(OR),
    log_lower = log(Lower_95_CI_Limit),
    log_upper = log(Upper_95_CI_Limit),
    SE_calc = (log_upper - log_lower) / (2 * 1.96),
    Sample_size_cat = case_when(
      Sample_size < 5000 ~ "Small (<5,000)",
      Sample_size >= 5000 & Sample_size < 20000 ~ "Medium (5,000–19,999)",
      Sample_size >= 20000 ~ "Large (≥20,000)",
      TRUE ~ "Unknown"
    )
  ) %>%
  filter(!is.na(OR), !is.na(Lower_95_CI_Limit), !is.na(Upper_95_CI_Limit))
```

```
# === Subgroup meta-analysis function ===
conduct_subgroup_meta <- function(data, subgroup_var, exposure_filter = NULL) {
  if (!is.null(exposure_filter)) data <- data %>% filter(Exposure_type == exposure_filter)
  subgroups <- unique(data[[subgroup_var]])[!is.na(unique(data[[subgroup_var]]))]
  results_list <- list()

  for (sg in subgroups) {
    subgroup_data <- data %>% filter(.data[[subgroup_var]] == sg)
    if (nrow(subgroup_data) >= 2) {
      tryCatch({
        meta_result <- rma(yi = log_OR, sei = SE_calc, data = subgroup_data, method = "REML")
        results_list[[sg]] <- list(
          subgroup = sg,
          n_studies = nrow(subgroup_data),
          pooled_OR = exp(meta_result$b[1]),
          ci_lower = exp(meta_result$ci.lb),
          ci_upper = exp(meta_result$ci.ub),
          i_squared = max(0, (meta_result$QE - meta_result$k + 1) / meta_result$QE * 100),
          p_value = meta_result$pval,
          tau_squared = meta_result$tau2,
          studies = paste(subgroup_data$Study_ID, collapse = ", "),
          meta_object = meta_result,
          data = subgroup_data
        )
      }, error = function(e) {
        results_list[[sg]] <<- list(subgroup = sg, n_studies = nrow(subgroup_data), error = e$message)
      })
    } else {
      results_list[[sg]] <- list(
        subgroup = sg,
        n_studies = nrow(subgroup_data),
        pooled_OR = subgroup_data$OR[1],
        ci_lower = subgroup_data$Lower_95_CI_Limit[1],
        ci_upper = subgroup_data$Upper_95_CI_Limit[1],
        studies = paste(subgroup_data$Study_ID, collapse = ", "),
        note = "Single study – no pooling",
        data = subgroup_data
      )
    }
  }
  return(results_list)
}

# === Run subgroup analyses ===
geo_perceived  <- conduct_subgroup_meta(data, "Geographic_region",
"Perceived_economic_circumstances")
design_perceived <- conduct_subgroup_meta(data, "Study_design",
"Perceived_economic_circumstances")
mm_perceived   <- conduct_subgroup_meta(data, "MM_definition",
"Perceived_economic_circumstances")
```

```
size_perceived <- conduct_subgroup_meta(data, "Sample_size_cat",
"Perceived_economic_circumstances")
exposure_overall <- conduct_subgroup_meta(data, "Exposure_type")

# === Subgroup table creation function ===
create_subgroup_table <- function(results_list, subgroup_name, exposure_name = "All exposures") {
  if (length(results_list) == 0) return(NULL)
  map_dfr(results_list, function(x) {
    data.frame(
      Subgroup = x$subgroup,
      N_studies = x$n_studies,
      Studies = str_trunc(x$studies, 50),
      Pooled_OR = ifelse(is.na(x$pooled_OR), "—", sprintf("%.2f", x$pooled_OR)),
      CI_95 = ifelse(is.na(x$ci_lower) | is.na(x$ci_upper), "—", sprintf("%.2f–%.2f", x$ci_lower,
x$ci_upper)),
      I_squared = ifelse(is.na(x$i_squared), "—", sprintf("%.1f%%", x$i_squared)),
      P_value = ifelse(is.na(x$p_value), "—", ifelse(x$p_value < 0.001, "<0.001", sprintf("%.3f",
x$p_value))),
      Notes = x$note %||% ""
    )
  })
}

# === Forest plot creation function ===
create_clean_forest_plot <- function(results_list, title, show_pooled = TRUE) {
  if (length(results_list) == 0) return(NULL)

  plot_data <- map_dfr(results_list, function(x) {
    study_data <- x$data %>%
      mutate(Study_Label = paste0(First_author, " (", Country, ")"),
             Type = "Individual",
             Subgroup_label = x$subgroup) %>%
      rename(CI_lower = Lower_95_CI_Limit, CI_upper = Upper_95_CI_Limit) %>%
      select(Study_Label, OR, CI_lower, CI_upper, Type, Subgroup_label)

    if (show_pooled && !is.na(x$pooled_OR) && x$n_studies >= 2) {
      pooled_data <- data.frame(
        Study_Label = "POOLED ESTIMATE",
        OR = x$pooled_OR, CI_lower = x$ci_lower, CI_upper = x$ci_upper,
        Type = "Pooled", Subgroup_label = x$subgroup
      )
      study_data <- bind_rows(study_data, pooled_data)
    }
    return(study_data)
  })

  plot_data <- plot_data %>%
    mutate(OR_CI_Label = sprintf("%.2f (%.2f–%.2f)", OR, CI_lower, CI_upper),
           is_pooled = Type == "Pooled") %>%
    group_by(Subgroup_label) %>%
    arrange(Subgroup_label, is_pooled, Study_Label) %>%
```

```
    mutate(y_pos = rev(row_number())) %>%
    ungroup()

  x_max <- max(plot_data$CI_upper, na.rm = TRUE)
  x_min <- min(plot_data$CI_lower, na.rm = TRUE)
  x_range <- x_max - x_min
  x_plot_min <- max(0.1, x_min - x_range * 0.1)
  x_plot_max <- x_max + x_range * 0.8
  text_position <- x_plot_max * 0.75

  ggplot(plot_data, aes(x = OR, y = y_pos)) +
    geom_vline(xintercept = 1, linetype = "dashed", colour = "grey50", size = 0.8) +
    geom_point(data = subset(plot_data, !is_pooled), shape = 15, size = 3, color = "darkblue") +
    geom_errorbarh(data = subset(plot_data, !is_pooled),
                   aes(xmin = CI_lower, xmax = CI_upper),
                   height = 0.2, size = 0.8, color = "darkblue") +
    geom_point(data = subset(plot_data, is_pooled),
               shape = 23, size = 5, fill = "red", color = "darkred", stroke = 1.5) +
    geom_errorbarh(data = subset(plot_data, is_pooled),
                   aes(xmin = CI_lower, xmax = CI_upper),
                   height = 0.3, size = 1.2, color = "darkred") +
    geom_text(aes(label = OR_CI_Label, x = text_position),
              hjust = 0, size = 3.5, color = "black", fontface = "bold") +
    facet_grid(Subgroup_label ~ ., scales = "free_y", space = "free") +
    scale_y_continuous(breaks = plot_data$y_pos, labels = plot_data$Study_Label) +
    scale_x_continuous(limits = c(x_plot_min, x_plot_max),
                       breaks = scales::pretty_breaks(n = 6)) +
    theme_bw() +
    theme(
      panel.grid.major.y = element_blank(),
      panel.grid.minor = element_blank(),
      panel.grid.major.x = element_line(color = "grey90", size = 0.5),
      strip.text = element_text(size = 12, face = "bold"),
      strip.background = element_rect(fill = "lightgrey", color = "black"),
      axis.text.y = element_text(size = 10, hjust = 1),
      axis.text.x = element_text(size = 10),
      axis.title.x = element_text(size = 12, face = "bold", margin = margin(t = 15)),
      plot.title = element_text(size = 14, face = "bold", hjust = 0.5),
      panel.spacing = unit(1, "lines"),
      plot.margin = margin(20, 100, 20, 20)
    ) +
    labs(x = "Odds Ratio (95% CI)", title = title)
}

# === Generate and save key subgroup plots ===
geo_plot_clean <- create_clean_forest_plot(geo_perceived,
                                          "Perceived Economic Circumstances by Geographic Region")
design_plot_clean <- create_clean_forest_plot(design_perceived,
                                             "Perceived Economic Circumstances by Study Design")
exposure_plot_clean <- create_clean_forest_plot(exposure_overall,
                                               "Multimorbidity Associations by Exposure Type")
```

```r
# Save plots (examples)
ggsave("subgroup_geographic_clean.png", geo_plot_clean, width = 14, height = 8, dpi = 300, bg =
"white")
ggsave("subgroup_design_clean.png", design_plot_clean, width = 14, height = 6, dpi = 300, bg =
"white")
ggsave("subgroup_exposure_clean.png", exposure_plot_clean, width = 14, height = 8, dpi = 300, bg
= "white")
```

Appendix O – Full text screening outcomes and exclusion reasons

Author	Year	Title	I or E	If E reason
Ballesteros	2021	Socioeconomic variation of multimorbidity in Colombian older adults.	I	Included
Dekhtyar	2019	Association Between Speed of Multimorbidity Accumulation in Old Age and Life Experiences: A Cohort Study	I	Included
Haas	2008	The developmental origins of health and disease in international perspective	I	Included
Henchoz	2019	Childhood adversity: A gateway to multimorbidity in older age?.	I	Included
Keetile	2023	The influence of childhood socioeconomic status on non-communicable disease risk factor clustering and multimorbidity among adults in Botswana: a life course perspective.	I	Included
Pati	2023	Childhood health and educational disadvantage are associated with adult multimorbidity in the global south: findings from a cross-sectional analysis of nationally representative surveys in India and Brazil	I	Included
Pavela	2016	Childhood Conditions and Multimorbidity Among Older Adults.	I	Included
Putnam	2013	Synergistic childhood adversities and complex adult psychopathology.	I	Included
Schramm	2021	Effects of individual and parental educational levels on multimorbidity classes: a register-based longitudinal study in a Danish population.	I	Included
Zhao	2023	Early-Life Factors and Multimorbidity Risk Later in Older Age: Evidence Based on CHARLS.	I	Included
Leigh	1993	Multidisciplinary findings on socioeconomic status and health.	E	Wrong Study Design
Nemececk	1999	Unequal Health	E	Wrong Study Design
Adjei	2024	Impact of Parental Mental Health and Poverty on the Health of the Next Generation: A Multi-Trajectory Analysis Using the UK Millennium Cohort Study.	E	Wrong Outcome
Agahi	2014	Social and economic conditions in childhood and the progression of functional health problems from midlife into old age.	E	Wrong Outcome
Aguiar	2024	Multimorbidity patterns and associated factors in a megacity: a cross-sectional study.	E	Wrong exposure
Ahrens	2014	Health Outcomes in Young Adults From Foster Care and Economically Diverse Backgrounds	E	Wrong Outcome
Akinosi	2002	Children and poverty	E	Wrong Study Design

Akre	1998	Social inequality and health--genetic causes?	E	Wrong Study Design
Alonso	2004	12-Month comorbidity patterns and associated factors in Europe: results from the European Study of the Epidemiology of Mental Disorders (ESEMeD) project.	E	Wrong Outcome
Alvarez	1985	Scale for measurement of socioeconomic level, in the health area	E	Wrong Population
Andersson	2016	Chronic Disease at Midlife: Do Parent-child Bonds Modify the Effect of Childhood SES?.	E	Wrong Outcome
Angell	1993	Privilege and health--what is the connection	E	Wrong Study Design
Anonymous/AJPH	2001	Resolution to improve the social conditions that contribute to health.	E	Wrong Study Design
Arpino	2018	Early-life conditions and health at older ages: The mediating role of educational attainment, family and employment trajectories.	E	Wrong Outcome
Arshadipour	2022	Impact of prenatal and childhood adversity effects around World War II on multimorbidity: results from the KORA-Age study.	E	Wrong Exposure
Atkinson	2023	Social engagement and allostatic load mediate between adverse childhood experiences and multimorbidity in mid to late adulthood: the Canadian Longitudinal Study on Aging.	E	Wrong Outcome
Baltzan	1999	Access to health care, socioeconomic status, and health.	E	Wrong Study Design
Bann	2017	Does an elite education benefit health? Findings from the 1970 British Cohort Study.	E	Wrong Outcome
Belsky	2015	Early adversity, elevated stress physiology, accelerated sexual maturation, and poor health in females.	E	Wrong Outcome
Benjet	2010	Chronic childhood adversity and onset of psychopathology during three life stages: childhood, adolescence and adulthood.	E	Wrong Exposure
Bensken	2024	Variation in multimorbidity by sociodemographics and social drivers of health among patients seen at community-based health centers.	E	Wrong exposure
Bishop	2022	Cohort Trends in the Burden of Multiple Chronic Conditions Among Aging U.S. Adults.	E	Wrong Outcome
Bodenmann	2009	Vulernability and health: Why a new rubric	E	Wrong Study Design

Borrel	2008	Health inequalities in Catalonia (Spain): are they actually in the political agenda?	E	Wrong Study Design
Bourassa	2023	Childhood Adversity and Midlife Health: Shining a Light on the Black Box of Psychosocial Mechanisms	E	Wrong Outcome
Brady	2024	The long term relationship between childhood Medicaid expansions and severe chronic conditions in adulthood.	E	Wrong Outcome
Breuer	2020	Caught in a web of trauma: Network analysis of childhood adversity and adult mental ill-health	E	Wrong Outcome
Bronnum-Hansen	2007	Social inequality in the burden of disease	E	Wrong Outcome
Burdett	2021	Military and demographic predictors of mental ill-health and socioeconomic hardship among UK veterans.	E	Wrong Outcome
CabiesesValdes	2012	Education and its relationship with income and health: a reflection on inequality in Chile	E	Wrong Study Design
Carney	2014	Wisconsin Longitudinal study	E	Wrong Study Design
Case	2005	The lasting impact of childhood health and circumstance	E	Wrong Outcome
Case	2010	Causes and consequences of early life health	E	Wrong Outcome
Castro	1991	Life below the threshold	E	Wrong Study Design
Chandrasekar	2023	Adverse childhood experiences and the development of multimorbidity across adulthood-a national 70-year cohort study.	E	Wrong Exposure
Chartier	2009	Separate and cumulative effects of adverse childhood experiences in predicting adult health and health care utilisation.	E	Wrong Exposure
Cohen	2010	Childhood socioeconomic status and adult health.	E	Wrong Study Design
Conroy	2010	Poverty Grown Up: How Childhood Socioeconomic Status Impacts Adult Health	E	Wrong Study Design
Consolazio	2024	Social inequalities in health within the City of Milan (Lombardy Region, Northern Italy): An ecological assessment.	E	Wrong Exposure

Craig	2023	Socioeconomic position, perceived weight, lifestyle risk, and multimorbidity in young adults aged 18 to 35 years: a Multi-country Study.	E	Wrong Exposure
Cundiff	2017	Moving up matters: Socioeconomic mobility prospectively predicts better physical health.	E	Wrong Outcome
Cunningham	1996	Plight of children of street	E	Wrong Study Design
Da Silveira	2023	Estimated multimorbity among young Brazilians: results of the 2019 National Health Survey	E	Wrong exposure
Dahl	1997	Health inequalities in later life in a social democratic welfare state.	E	Wrong Outcome
DalGrande	2015	Effect of social mobility in family financial situation and housing tenure on mental health conditions among South Australian adults: results from a population health surveillance system, 2009 to 2011	E	Wrong Outcome
Davey-Smith	1993	Socioeconomic differentials in wealth and health.	E	Wrong Study Design
deAlmeida	2021	Risk factors for common mental disorders in health care workers in the city of Diamantina, state of Minas erais	E	Wrong Outcome
Deng	2024	Associations of schooling type, qualification type and subsequent health in mid-adulthood: evidence from the 1970 British Cohort Study.	E	Wrong Outcome
Diderichsen	1992	Effects of economic change on male morbidity in neighbouring industrial and rural municipalities in northern Sweden.	E	Wrong Outcome
Drakopolous	2011	Childhood socioeconomic deprivation and later adulthood health	E	Wrong Outcome
Du	2021	Direct and Indirect Associations between Family Residential Mobility, Parent Functioning, and Adolescent Behavioural Health.	E	Wrong Outcome
Duncan	2010	Early-childhood poverty and adult attainment, behaviour, and health.	E	Wrong Outcome
Elstad	2005	Childhood adversities and health variations among middle-aged men: a retrospective lifecourse study	E	Wrong Outcome
Emerson	2009	Relative child poverty, income inequality, wealth, and health.	E	Wrong Study Design
Etherington	2016	Heterogeneity in chronic disease outcomes among women and men in midlife: examining the role of	E	Wrong

		stability and change in childhood economic hardship.		Outcome
Falbo	2009	Alternate models of sibling status effects on health in later life.	E	Wrong Outcome
Filigrana	2024	LifeCourse Socioeconomic Position and Ideal Cardiovascular Health in Hispanic/Latino Adults of the Hispanic Community Health Study/Study of Latinos.	E	Wrong Outcome
Flaskerud	2012	Social determinants of health status	E	Wrong Study Design
Foley	2000	Poor beginnings, poor health: A 30-year exploration of socioeconomic position and health	E	Wrong Study Design
Font	2016	Pathways from childhood abuse and other adversities to adult health risks	E	Wrong Outcome
Fujiwara	2011	Association of childhood adversities with the first onset of mental disorders in Japan: Results from the World Mental Health Japan, 2002-2004	E	Wrong Outcome
Fuller-Rowell	2021	A Changing Landscape of Health Opportunity in the United States: Increases in the Strength of Association Between Childhood Socioeconomic Disadvantage and Adult Health Between the 1990s and the 2010s.	E	Wrong Outcome
Gasik	2024	The Impact of Childhood Adversity on Life Course Alcohol Use Patterns and Health Status Among People Living with HIV	E	Wrong Population
Gaska	2018	Patterns of Adverse Experiences and Health Outcomes Among Women Veterans	E	Wrong Outcome
Giger	2006	Re-defining the term health disparities	E	Wrong Study Design
Gilman	2002	Childhood socioeconomic status, life course pathways and adult mental health.	E	Wrong Study Design
Golba	2015	Socioeconomic status and cardiovascular health in the changing world	E	Wrong Study Design
Goosby	2013	Early Life Course Pathways of Adult Depression and Chronic Pain	E	Duplicate data
Gopalan	1983	Qualitative dimension of the population problem	E	Wrong Study Design
Grundy	2003	Health inequalities in the older population: the role of personal capital, social resources and socio-economic circumstances.	E	Wrong Exposure
Guralnik	1997	Race, ethnicity, and health outcomes--unraveling the mediating role of socioeconomic status.	E	Wrong Study

				Design
Gustaffson	2017	What role does adolescent neighborhood play for adult health? A cross-classified multilevel analysis of life course models in Northern Sweden.	E	Wrong Outcome
Haapanen	2024	Early growth, stress, and socioeconomic factors as predictors of the rate of multimorbidity accumulation across the life course: a longitudinal birth cohort study.	E	Wrong Outcome
Haas	2008	Trajectories of functional health: The 'long arm' of childhood health and socioeconomic factors	E	Wrong Outcome
Hammarstrom	2011	Mechanisms for the social gradient in health: results from a 14-year follow-up of the Northern Swedish Cohort.	E	Wrong Outcome
Hank	2013	Changes in Older Europeans' Health Across Two Waves of SHARE: Life-Course and Societal Determinants	E	Wrong Outcome
Hansen	2022	Educational attainment in young adulthood and self-rated health in midlife – Does allostatic load mediate the association?	E	Wrong Outcome
Harber-Aschan	2020	Beyond the social gradient: the role of lifelong socioeconomic status in older adults' health trajectories.	E	Wrong Outcome
Hazell	2022	Socio-economic inequalities in adolescent mental health in the UK: multiple socio-economic indicators and reporter effects	E	Wrong Outcome
HendersonLeShae	2024	Lifetimes of Vulnerability: Childhood Adversity, Poor Adult Health, and the Criminal Legal System	E	Wrong Outcome
Herrenkohl	2010	Effects of childhood conduct problems and family adversity on health, health behaviours, and service use in early adulthood: tests of developmental pathways involving adolescent risk taking and depression.	E	Wrong Outcome
Homan	2020	Longitudinal Health Consequences of Childhood Adversity: The Mediating Role of Purpose in Life	E	Wrong Outcome
House	2005	Continuity and change in the social stratification of aging and health over the life course: evidence from a nationally representative longitudinal study from 1986 to 2001/2002 (Americans' Changing Lives Study).	E	Wrong Outcome
Huang	2011	Do Early-Life Conditions Predict Functional Health Status in Adulthood? The Case of Mexico	E	Wrong Outcome
Huber	1994	Explaining the connection between privelege and health	E	Wrong Study Design
Hussain	1999	Poverty, income and health	E	Wrong Study

				Design
Iniguez	2016	Adverse Childhood Experiences and Health in Adulthood in a Rural Population-Based Sample.	E	Wrong Outcome
Iversen	2007	Influence of childhood adversity on health among male UK military personnel.	E	Wrong Outcome
Jackson	2015	Body mass index and socioeconomic position are associated with 9-year trajectories of multimorbidity: A population-based study.	E	Wrong Exposure
Jackson	2016	Lifestyle and Socioeconomic Determinants of Multimorbidity Patterns among Mid-Aged Women: A Longitudinal Study.	E	Wrong Exposure
Jayasinghe	2005	Poverty, inequality and health: issues relevant to South Asia.	E	Wrong Study Design
Jennings	2024	Exposure to Adversity and its Impact on Later Life Cognitive, Mental, and Physical Health.	E	Wrong Outcome
Jiang	2024	Long-term impacts of socioeconomic status and childhood trauma on depression in older adults: social work strategies.	E	Wrong Outcome
Jin	2023	Childhood socioeconomic disadvantage and risk of physical multimorbidity in later life: The mediating role of depression.	E	Duplicate data
Joensuu	2016	Clustering of adversity in young adults on disability pension due to mental disorders: a latent class analysis	E	Wrong Exposure
Johnson	2011	Psychological and physical health at age 70 in the Lothian Birth Cohort 1936: links with early life IQ, SES, and current cognitive function and neighborhood environment	E	Wrong Outcome
Johnson	2011	Early-life origins of adult disease: national longitudinal population-based study of the United States.	E	Wrong Outcome
Jolly	1991	The impact of poverty and disadvantage on child health	E	Wrong Study Design
Jungo	2022	Life course socioeconomic conditions, multimorbidity and polypharmacy in older adults	E	Duplicate data
Kaplan	1997	Whither studies on the socioeconomic foundations of population health?	E	Wrong Study Design
Katikireddi	2017	The contribution of risk factors to socioeconomic inequalities in multimorbidity across the lifecourse: a longitudinal analysis of the Twenty-07 cohort.	E	Wrong Exposure
Kessler	2010	Childhood adversities and adult psychopathology in the WHO World Mental Health Surveys	E	Wrong Outcome

Khanolkar	2021	Socioeconomic inequalities in prevalence and development of multimorbidity across adulthood: A longitudinal analysis of the MRC 1946 National Survey of Health and Development in the UK.	E	Wrong Outcome
Kinoulty	2005	Monitor	E	Wrong Study Design
Kohler	2007	Why do children feel worse? Importance of relative poverty, social capital and status for children's health	E	Wrong Study Design
Kuehn	2017	Neighborhoods are key for heart health	E	Wrong Study Design
Kuehn	2019	Childhood Hardships Contribute to Poor Adult Health: The Journal of the American Medical Association	E	Wrong Study Design
Kwon	2023	Early-Life Socioeconomic Disadvantage and Health in Late Middle-Age: Importance of Heterogeneous Income Trajectories.	E	Duplicate data
Larson	2007	The effects of childhood SES and living circumstances on adult health and longevity	E	Wrong Study Design
Lee	2021	Associations Between Adverse Childhood Experiences and Adult Health Outcomes: Exploring Gender Differences	E	Wrong Outcome
Lee	2017	Childhood Adversities Associated with Poor Adult Mental Health Outcomes in Older Homeless Adults: Results From the HOPE HOME Study.	E	Wrong Outcome
Lehman	2005	Relation of childhood socioeconomic status and family environment to adult metabolic functioning in the CARDIA study.	E	Wrong Outcome
Lennartson	2018	Social class and infirmity. The role of social class over the life-course.	E	Wrong Outcome
Li	2020	Effects of multi-dimensional social capital on mental health of children in poverty: An empirical study in Mainland China	E	Wrong Outcome
Li	2024	The impact of neighborhood deprivation on mental health and quality of life in children and adolescents during the COVID-19 pandemic: Findings from the COPSY Hamburg study	E	Wrong Outcome
Lin	2021	Adverse Childhood Experiences and Subsequent Chronic Diseases Among Middle-aged or Older Adults in China and Associations With Demographic and Socioeconomic Characteristics.	E	Wrong Exposure
Lipowicz	2007	Socioeconomic status during childhood and health status in adulthood: the Wroclaw growth study.	E	Wrong Outcome
Liu	2022	The cumulative effects of lifestyle on different patterns of multimorbidity among children and adolescents aged 9-18 in Tianjin City]	E	Wrong Outcome

Lonsdale	1994	Explaining the connection between privelege and health	E	Wrong Study Design
Loxton	2021	The impact of adverse childhood experiences on the health and health behaviours of young Australian women	E	Wrong Outcome
Luo	2005	The impact of childhood and adult SES on physical, mental, and cognitive well-being in later life.	E	Wrong exposure
Lurie	2007	Health disparities and access to health.	E	Wrong Study Design
Lynch	1997	Why do poor people behave poorly? Variation in adult health behaviours and psychosocial...	E	Wrong Outcome
Mackay	2016	Good start vital for kiuds	E	Wrong Study Design
Mackenzie	2014	Prevalence and predictors of persistent versus remitting mood, anxiety, and substance disorders in a national sample of older adults.	E	Wrong Outcome
Macleod	2005	Is subjective social status a more important determinant of health than objective social status? Evidence from a prospective observational study of Scottish men	E	Wrong Outcome
Macrae	1994	Socioeconomic deprivation and health and the ecological fallacy.	E	Wrong Study Design
Madero-Cabib	2019	Advantages and disadvantages across the life course and health status in old age among women in Chile.	E	Wrong Outcome
Manor	2012	Effects of socio Economic Position in Childhood and Adulthood on Cardiometabolic Risk Factors: The Jerusalem Perinatal Family Follow-Up Study	E	Wrong Outcome
Mattingly	2013	Childhood Poverty, Race and Young Adult Health Outcomes.	E	Wrong Outcome
McCauley	2015	Adverse Childhood Experiences and Adult Health Outcomes Among Veteran and Non-Veteran Women.	E	Wrong Outcome
McEniry	2019	Displacement due to armed conflict and violence in childhood and adulthood and its effects on older adult health: The case of the middle-income country of Colombia.	E	Wrong Outcome
McLaughlin	2011	Childhood socio-economic status and the onset, persistence, and severity of DSM-IV mental disorders in a US national sample	E	Wrong Outcome
McLaughlin	2010	Childhood adversities and adult psychopathology in the National Comorbidity Survey Replication (NCS-R) III: Associations with functional impairment related to DSM-IV disorders	E	Wrong Outcome

Melchior	2007	Why Do Children from Socioeconomically Disadvantaged Families Suffer from Poor Health When They Reach Adulthood? A Life-Course Study	E	Wrong Outcome
Merrick	2019	Vital Signs: Estimated Proportion of Adult Health Problems Attributable to Adverse Childhood Experiences and Implications for Prevention - 25 States, 2015–2017	E	Wrong Outcome
Miquillin	2015	Demographic, socioeconomic, and health profile of working and non-working Brazilian children and adolescents: an analysis of inequalities	E	Wrong Outcome
Montgomery	2013	Relationship Among Adverse Childhood Experiences, History of Active Military Service, and Adult Outcomes: Homelessness, Mental Health, and Physical Health	E	Wrong Outcome
Morris	1996	The influence of socioeconomic position on health--and vice versa.	E	Wrong Study Design
Morrow	1982	The fundamental influence of political, social and economic factors on health and health care.	E	Wrong Study Design
Muller	1994	Explaining the connection between privelege and health	E	Wrong Study Design
Munroe	1984	Health and wealth in four societies	E	Wrong Study Design
Neeleman	2001	The distribution of psychiatric and somatic III health: associations with personality and socioeconomic status.	E	Wrong Exposure
Nelson	2020	Adversity in childhood is linked to mental and physical health throughout life.	E	Wrong Study Design
Nilsson	2008	Win an Oscar--live longer. Psychosocial conditions affect mortality, morbidity and health	E	Wrong Study Design
Non	2020	Optimism and Social Support Predict Healthier Adult Behaviours Despite Socially Disadvantaged Childhoods.	E	Wrong Outcome
O'Brien	2012	Healthy, wealthy, wise? Psychosocial factors influencing the socioeconomic status-health gradient.	E	Wrong Outcome
Oldehinkel	2015	A longitudinal perspective on childhood adversities and onset risk of various psychiatric disorders	E	Wrong Outcome
Owara	2023	Modeling the Natural Course of Atopic Multimorbidity: Correlates of Early-Life States and Exposures.	E	Wrong Study Design
Palloni	2005	The influence of early conditions on health status among elderly Puerto Ricans.	E	Wrong Outcome

Pappas	1994	Elucidating the relationships between race, socioeconomic status, and health.	E	Wrong Study Design
Patterson	2012	Symptoms of illness in late adulthood are related to childhood social deprivation and misfortune in men but not in women.	E	Wrong Outcome
Patterson	2014	Setting the stage for chronic health problems: cumulative childhood adversity among homeless adults with mental illness in Vancouver, British Columbia	E	Wrong Exposure
Peck	1992	Childhood environment, intergenerational mobility, and adult health--evidence from Swedish data.	E	Wrong Outcome
Peikes	2000	Psychosocial determinants of midlife health: A person -centered approach	E	Wrong Outcome
Pengpid	2024	Socioeconomic position and physical and mental health among middle-aged and older adults: Cross-sectional and longitudinal results from a national community sample in Thailand.	E	Wrong Outcome
Perez	2024	Cardiovascular Health Among Young Men and Women in Puerto Rico as Assessed by the Life's Essential 8 Metrics.	E	Wrong Outcome
Ploubidis	2014	Lifelong Socio Economic Position and biomarkers of later life health: testing the contribution of competing hypotheses.	E	Wrong Outcome
Poulton	2002	Association between children's experience of socioeconomic disadvantage and adult health: a life-course study	E	Wrong Outcome
Power	2007	Life-course influences on health in British adults: effects of socio-economic position in childhood and adulthood	E	Wrong Outcome
Power	2002	Childhood adversity still matters for adult health outcomes	E	Wrong Study Design
Rahkonen	1997	Past or present? Childhood living conditions and current socioeconomic status as determinants of adult health.	E	Wrong Outcome
Ramos	2007	Impact of socioeconomic status on brazilian elderly health	E	Wrong Outcome
Reading	2008	Why do children from socioeconomically disadvantaged families suffer from poor health when they reach adulthood? A life-course study.	E	Wrong Outcome
Reuben	2016	Lest we forget: comparing retrospective and prospective assessments of adverse childhood experiences in the prediction of adult health.	E	Wrong Outcome
Reyes-Ortiz	2023	Racial Discrimination and Multimorbidity Among Older Adults in Colombia: A National Data Analysis.	E	Wrong Exposure

Riem	2019	Childhood Adversity and Adult Health: The Role of Developmental Timing and Associations With Accelerated Aging.	E	Wrong Outcome
Ritchie	2014	Why should we all focus on health inequalities in the foetus and early childhood?	E	Wrong Study Design
Roberg	1994	Explaining the connection between privelege and health	E	Wrong Study Design
Robert	1996	SES differentials in health by age and alternative indicators of SES.	E	Wrong Outcome
Robson	2020	The Relationship of Early-Life Adversity With Adulthood Weight and Cardiometabolic Health Status in the 1946 National Survey of Health and Development	E	Wrong Outcome
Roos	2013	Relationship between adverse childhood experiences and homelessness and the impact of axis I and II disorders.	E	Wrong Outcome
Salinas Rodrigues	2019	Socioeconomic inequalities in health and nutrition among older adults in Mexico.	E	Wrong Outcome
Samari	2019	Socioeconomic Status and the Physical and Mental Health of Arab and Chaldean Americans in Michigan.	E	Wrong Exposure
Santoro	2021	Childhood adversity and physical health among Asian Indian emerging adults in the United State	E	Wrong exposure
Santoro	2018	Childhood adversity, health and quality of life in adults with intellectual and developmental disabilities.	E	Wrong Exposure
Satterthwaite	1993	The impact on health of urban environments.	E	Wrong Study Design
Saxton	2020	Early life adversity increases the salience of later life stress: an investigation of interactive effects in the PSID.	E	Wrong Exposure
Schilling	2008	The impact of cumulative childhood adversity on young adult mental health: Measures, models, and interpretations	E	Wrong Outcome
Sederer	2016	The social determinants of mental health	E	Wrong Study Design
Seo	2010	Analysis for the impact of adulthood and childhood socioeconomic positions and intergenerational social mobility on adulthood health	E	Wrong Outcome
Sheffler	2024	Sleep Quality as a Critical Pathway Between Adverse Childhood Experiences and Multimorbidity and the Impact of Lifestyle	E	Wrong Exposure

Sheffler	2025	Sleep Quality as a Critical Pathway Between Adverse Childhood Experiences and Multimorbidity and the Impact of Lifestyle.	E	Wrong exposure
Sheikh	2018	Exposure is hildhood adversity measured with ACE Scale (abuse, neglect, household dysfunction), summed into a score (range 0–6); lacks explicit childhood SEC metrics. Outcome – Number of chronic conditions (e.g., diabetes, asthma, depression) summed into a total score via a health history questionnaire, representing a count of conditions (relevant for multimorbidity). Methods – Regression analyses examine associations between ACEs and chronic condition count, mediated by anger/stress. While outcome is relevant, exposure fails to meet inclusion criteria	E	Wrong exposure
Shen	2014	Direct and indirect effects of childhood conditions on survival and health among male and female elderly in China.	E	Wrong Outcome
Shevlin	2019	Social, Familial and Psychological Risk Factors for Endocrine, Nutritional and Metabolic Disorders in Childhood and Early Adulthood: a Birth Cohort Study Using the Danish Registry System.	E	Wrong Outcome
Siegel	2009	Inequalities in income and health related to child poverty.	E	Wrong Study Design
Siegrist	2002	Social capital and health	E	Wrong Study Design
Slack	2017	The Complex Interplay of Adverse Childhood Experiences, Race, and Income.	E	Wrong Outcome
Smith	1997	Socio-economic differentials in health: the role of nutrition	E	Wrong Outcome
Sosnowski	2022	Adverse childhood experiences and comorbidity in a cohort of people who have injected drugs	E	Wrong Population
Sreedhar	2019	Multimorbidity and multiple social disadvantage in a New Zealand high-needs free primary healthcare clinic population: a cross-sectional study	E	Wrong Exposure
Stannard	2024	Mapping domains of early life determinants of future multimorbidity across three UK longitudinal cohort studies.	E	Wrong Outcome
Starfield	1992	Effects of poverty on health status	E	Wrong Study Design
Starfield	1982	Child health and socioeconomic status	E	Wrong Study Design
Stewart-Brown	2005	Parent-child relationships and health problems in adulthood in three UK national birth cohort studies	E	Wrong Exposure

Stillerman	2018	Childhood adversity and lifelong health: From research to action.	E	Wrong Study Design
Subramanyam	2011	Research on social inequalities in health in India.	E	Wrong Study Design
Tabatabei	2024	The Role of Childhood Circumstances on Social Conditions and Health of Middle-Aged and Older Adults: Ardakan Cohort Study on Aging (ACSA).	E	Wrong Outcome
Taillieu	2020	Clinical Epidemiology of Alcohol Use Disorders in Military Personnel versus the General Population in Canada	E	Wrong Outcome
Takahashi	2016	A novel housing-based socioeconomic measure predicts hospitalisation and multiple chronic conditions in a community population.	E	Wrong Exposure
Tampubolon	2015	Growing Up in Poverty, Growing Old in Infirmity	E	Wrong Outcome
Tao	2021	The Effects of Childhood Circumstances on Health in Middle and Later Life: Evidence From China.	E	Wrong Outcome
Teixeira	2024	Trajectory of Multiple Chronic Conditions and Associated Factors Among Noninstitutionalized Adults Aged 60 Years or Older in Southern Brazil	E	Wrong Exposure
Thoma	2021	Health, stress, and well-being in Swiss adult survivors of child welfare practices and child labor: Investigating the mediating role of socio-economic factors.	E	Wrong Outcome
Tonmyr	2005	The relationship between childhood adverse experiences and disability due to physical health problems in a community sample of women.	E	Wrong Outcome
Torres	2018	Lifetime Socioeconomic Status and Late-life Health Trajectories: Longitudinal Results From the Mexican Health and Aging Study.	E	Wrong Outcome
Traub	2017	Modifiable Resilience Factors to Childhood Adversity for Clinical Pediatric Practice	E	Wrong Study Design
Trevisan	2025	Prevalence of depressive symptoms and associated factors in Brazilian older adults: 2019 Brazilian National Health Survey.	E	Wrong Outcome
Trossman	2021	How do adverse childhood experiences impact health? Exploring the mediating role of executive functions.	E	Wrong Outcome
Tucker Seeley	2011	Lifecourse socioeconomic circumstances and multimorbidity among older adults.	E	Duplicate data
Turner	2016	Childhood adversity and adult health: Evaluating intervening mechanisms	E	Wrong Outcome
Umberson	2014	Race, Gender, and Chains of Disadvantage: Childhood Adversity, Social Relationships, and Health	E	Wrong

				Outcome
Vable	2018	Do the health benefits of education vary by sociodemographic subgroup? Differential returns to education and implications for health inequities.	E	Wrong Outcome
Van de Mheen	1997	The contribution of childhood environment to the explanation of socio-economic inequalities in health in adult life: a retrospective study.	E	Wrong Outcome
Vasquez	2019	Association Between Adverse Childhood Events and Multimorbidity in a Racial and Ethnic Diverse Sample of Middle-Aged and Older Adults.	E	Wrong Exposure
Villadsen	2023	Clustering of adverse health and educational outcomes in adolescence following early childhood disadvantage: population-based retrospective UK cohort study.	E	Wrong Outcome
Viner	2005	Adult Health and Social Outcomes of Children Who Have Been in Public Care: Population-Based Study	E	Wrong Outcome
Waaler	2006	Is Norway the best country to live in?	E	Wrong Study Design
Wade	2016	Household and community-level Adverse Childhood Experiences and adult health outcomes in a diverse urban population.	E	Wrong Outcome
Waikato times	2012	Poverty condemns children to poor health	E	Wrong Study Design
Walker	2005	Socio-economic status: an insufficiently appreciated component of US health.	E	Wrong Study Design
Walker	1986	Health--ill health in the rich and the poor.	E	Wrong Study Design
Wang	2019	Childhood socioeconomic circumstances, social status, and health in older age: Are they related in China?.	E	Wrong Outcome
Ward	2018	Investigating equalisation of health inequalities during adolescence in four low-income and middle-income countries: an analysis of the Young Lives cohort study.	E	Wrong Outcome
Weitoft	2008	Health and social outcomes among children in low-income families and families receiving social assistance--a Swedish national cohort study.	E	Wrong Outcome
Wen	2011	The Effects of Childhood, Adult, and Community Socioeconomic	E	Wrong Outcome
Whitaker	2014	Adverse childhood experiences, dispositional mindfulness, and adult health.	E	Wrong exposure
Wickrama	2013	Heterogeneity in multidimensional health trajectories of late old years	E	Wrong

		and socioeconomic stratification: a latent trajectory class analysis.		Outcome
Wickrama	2005	Early adversity and later health: the intergenerational transmission of adversity through mental disorder and physical illness.	E	Wrong Outcome
Willson	2016	Life Course Pathways of Economic Hardship and Mobility and Midlife Trajectories of Health.	E	Wrong Outcome
Wolitsky-Taylor	2017	The Effects of Childhood and Adolescent Adversity on Substance Use Disorders and Poor Health in Early Adulthood	E	Wrong Outcome
Yang	2020	Childhood adversity and trajectories of multimorbidity in mid-late life: China health and longitudinal retirement study.	E	Wrong Outcome
Yang	2017	Early-Life Socioeconomic Status and Adult Physiological Functioning: A Life Course Examination of Biosocial Mechanisms.	E	Wrong Outcome
Yang	2021	Childhood adversity and trajectories of multimorbidity in mid-late life: China health and longitudinal retirement study	E	Wrong Exposure
Yaqiang	2015	Does childhood nutrition predict health outcomes in adulthood	E	Wrong Exposure
Yilmaz	2018	Effects of early life factors on the health and quality of life of older adults.	E	Wrong Outcome
Yu Cheng	2024	The relationship between childhood SES and health in middle and old age: evidence from China.	E	Wrong Outcome
Zhang	2022	Adverse childhood experiences in relation to comorbid cardiovascular diseases and diabetes among middle-aged and old adults in China.	E	Wrong Outcome
Zheng	2023	Understanding the health decline of Americans in boomers to millennials.	E	Wrong Outcome
Zheng	2022	Associations between childhood maltreatment and educational, health and economic outcomes among middle-aged Chinese: The moderating role of relative poverty.	E	Wrong Outcome
Zimmer	2016	Childhood socioeconomic status, adult socioeconomic status, and old-age health trajectories: Connecting early, middle, and late life	E	Wrong Outcome
Ziol-Guest	2012	Early childhood poverty, immune-mediated disease processes, and adult productivity.	E	Wrong Outcome
ZunZunegui	2009	Explaining health differences between men and women in later life: A cross-city comparison in Latin America and the Caribbean	E	Wrong Outcome

References

1 Barnett, K. *et al.* Epidemiology of multimorbidity and implications for health care, research, and medical education: a cross-sectional study. *The Lancet* **380**, 37-43 (2012).

2 Ho, I. S. *et al.* Measuring multimorbidity in research: Delphi consensus study. *BMJ medicine* **1** (2022).
